# Supplementary material for: Effectiveness–implementation hybrid type 2 study evaluating an intervention to support ‘information work’ in dementia care: an implementation study protocol
Source: BMJ Open. 2020 Dec 8;10(12):e038397. doi: 10.1136/bmjopen-2020-038397 (PMC7725094; doi:10.1136/bmjopen-2020-038397)
Supplement: Supplementary data [file bmjopen-2020-038397supp001.pdf]

Supplementary material

Interview guide – People living with dementia (interview 1)

Preamble	I'd like to ask you to tell me more about your experience of using this leaflet that I believe you recently received…
Receiving the leaflet	1. Do you remember receiving this leaflet? When? Where? From whom?
	2. When you were given the leaflet, did the supplier explained what information were available and how to use it?
Information seeking process before the leaflet was offered or alternative to the leaflet	3. [If not recently diagnosed] – Before you were given the leaflet, how did you go about accessing information on dementia or local support available for yourself? How did you find this process?
	4. Are you aware of similar sources of information? How have you found out about them?
	5. What do you think of these other information materials available?
	6. If you are aware of similar resources, have you used them? If not, why?
Expected or initial use	7. What are your impressions of the new leaflet in terms of its usefulness?
	8. Have you used it already? If yes, when did you use it? If not, do you think you will use it in the future?
	9. If you used it, what particular information were you looking for? Could you find the information you were looking for?
	10. Have you acted upon the information you located – e.g. have you already contacted/accessed a service?
	11. Were there any particular aspects you think may be useful to you over the coming months?
Satisfaction	12. Is there anything in particular you like or do not like about the leaflet?
	13. Are there aspects of the leaflet you might want to change in order to make it better for your purposes?
Conclusions	14. Is there anything else that you'd like to add?
Agreeing follow up interview	
Thank you	

Interview guide – People living with dementia (follow up)

Preamble	I'd like to ask you to tell me more about your experience of using this leaflet that you received some months ago…
Use	1. Since you have been given a copy by your CPN, have you used the leaflet?
	2. If not, why? Do you plan to use it in the future?
	3. If yes, when did you use it? What information were you looking for? Could you find what you were looking for?
	4. Have you acted upon the information you found, e.g. have you contacted/accessed a service?
Barriers and facilitators	5. What helped you to use the leaflet e.g. you received a printed copy or a trusted person gave it to you?
	6. What prevented you from using the leaflet, e.g. format or content?
Information seeking process before the leaflet was offered or alternative to the leaflet	7. Since we last spoke, have you found out about other information resources for people living with dementia? How have you found them?
	8. If you are aware of similar resources, have you used them? If you haven't used them, why not?
	9. What do you think of these other information resources?
Impact	10. Has the leaflet changed the way you look for information about services available locally? If yes, in what way? If not, why?
	11. Has the leaflet changed the care and support you receive? If yes, in what way? If not, why?
	12. Since receiving the leaflet, are you in touch with organizations you previously did not know?
Satisfaction	13. What do you like most/ least about the leaflet?
	14. Is there anything you would change?
Future use	15. Would you expect to receive a new copy of the leaflet if it is updated in the future? How often? From whom?
	16. Would you keep using the leaflet in the future?
	17. Is there anything else that you'd like to add?
Thank you	

Interview guide – Family carers (interview 1)

Preamble	I'd like to ask you to tell me more about your experience of using this leaflet that I believe you recently received…
Receiving the leaflet	1. Do you remember receiving this leaflet? When? Where? From whom?
	2. When you were given the leaflet, did the supplier explained what information were available and how to use it?
Information seeking process before the leaflet was offered or alternative to the leaflet	3. [If person with dementia not recently diagnosed] – Before you were given the leaflet, how did you go about accessing information on dementia or local support available for yourself or for <name of the person living with dementia>? How did you find this process?
	4. Are you aware of similar sources of information for yourself or for <name of person living with dementia>? How have you found out about them?
	5. What do you think of these other information materials available?
	6. If you are aware of similar resources, have you used them? If not, why?
Expected or initial use	7. What are your impressions of the new leaflet in terms of its usefulness?
	8. Have you used it already? If yes, when did you use it? If not, do you think you will use it in the future?
	9. If you used it, what particular information were you looking for? Could you find the information you were looking for?
	10. Have you acted upon the information you located – e.g. have you already contacted/accessed a service for yourself or for <name of the person living with dementia>?
	11. Were there any particular aspects you think may be useful to you over the coming months?
Satisfaction	12. Is there anything in particular that you like or do not like about the leaflet?
	13. Are there aspects of the leaflet you might want to change in order to make it better for your purposes?
Conclusions	14. Is there anything else that you'd like to add?
Agreeing follow up interview	
Thank you	

BMJ Publishing Group Limited (BMJ) disclaims all liability and responsibility arising from any reliance placed on this supplemental material which has been supplied by the author(s)

Interview guide – Family carers (follow up)

Preamble	I'd like to ask you to tell me more about your experience of using the leaflet that you received some months ago…
Use	1. Since you have been given a copy by your CPN, have you used the leaflet?
	2. If not, why? Do you plan to use it in the future?
	3. If yes, when did you use it? What information were you looking for? Could you find what you were looking for?
	4. Have you acted upon the information you found, e.g. have you contacted/accessed a service for yourself or for <name of the person living with dementia>?
Barriers and facilitators	5. What helped you to use the leaflet e.g. you received a printed copy or a trusted person gave it to you?
	6. What prevented you from using the leaflet, e.g. format or content?
Information seeking process before the leaflet was offered or alternative to the leaflet	7. Since we last spoke, have you found out about other information resources for carers of somebody living with dementia? How have you found them?
	8. If you are aware of similar resources, have you used them? If you haven't used them, why not?
	9. What do you think of these other information resources?
Impact	10. Has the leaflet changed the way you look for information about local services for yourself or for <name of the person living with dementia>? If yes, in what way? If not, why?
	11. Has the leaflet changed the care and support you or <name of the person living with dementia> receive? If yes, in what way? If not, why?
	12. Since receiving the leaflet, are you in touch with organizations you previously did not know?
Satisfaction	13. What do you like most/least about the leaflet?
	14. Is there anything you would change?
Future use	15. Would you expect to receive a new copy of the leaflet if it is updated in the future? How often? From whom?
	16. Would you keep using the leaflet in the future?
	17. Is there anything else that you'd like to add?
Thank you	

Interview guide – CPNs from Community Mental Health Services for Older People

Preamble	I'd like to ask you about your experience of using the leaflet in clinical practice…
Implementation strategy	1. How were you made aware that the leaflet would have been available to you to use in their clinical work?
	2. Were you given any guidance about when and how to hand the leaflet out to patients?
	3. Were you made aware of the type of patients who should have been given the leaflet?
	4. How was the leaflet made available to you? E.g. was it stored somewhere on your IT system and you had to print it out?
	5. When have you started using it? For how long will you use it in your clinical practice?
Actual use	6. How are you using the leaflet with your patients, e.g. for signposting patients or families to local services?
	7. Once you started using the leaflet in clinical practice, have you changed something in how you are using it? If yes, what have you changed? Why did you change it?
	8. Do you think you reached the expected group(s) of users?
	9. What are the factors that influenced the actual use of the leaflet in your clinical practice?
Impact	10. Has the leaflet changed how your appointments with the patients and families take place? If yes, what has changed? Why has it changed?
	11. Do you think that the leaflet has changed patients' and family carers' perceptions about local care and support available to them?
	12. Do you think that the leaflet has changed how people with dementia and family carers access and use local services?
Future use	13. Do you plan to keep using it? Do you think it should be updated regularly?
	14. Do you think we should change anything about the leaflet (e.g. thinking about its content or its design) or about how it is used in clinical practice?
Other strategies	15. Are you aware of the current strategies or priorities in the area of dementia (e.g. these could be national, local or identified by the organization you work for)? Do you think the leaflet fits with the current strategies or priorities?
Co-creation – Process	16. Are you aware that the leaflet was designed and developed with inputs from both people living with dementia, their family carers, and a wide range of local organizations providing or commissioning care and support for them? If yes, did you contribute to the design and development of the leaflet? How?

Co-creation – Impact	17. In your opinion did this approach influence the use of the leaflet in clinical practice? In what ways?
Conclusion	18. Do you have any final comment on the leaflet? Or on your experience of using it with your patients and their families? Or on your experience of using it in clinical practice?
	19. Is there anything else that you'd like to add?
Thank you	

Interview guide - Team and service managers from Community Mental Health Services for Older People

Preamble	I'd like to ask you about your experience of using the leaflet in your organization…
Implementation strategy	1. Can you please describe how you organized the dissemination of the leaflet in practice among your staff? How did you inform the staff that the leaflet would have been made available to them to use in their clinical work? How you given your staff any guidance about when and how to hand the leaflet out to patients?
	2. How you identified the type of patients who should have been given the leaflet? Was this made clear to your staff?
	3. How was the leaflet embedded in the clinical practice of CPNs?
	4. How is it made available to them e.g. was it stored somewhere on your IT system and CPNs can print it out?
	5. When have you started using it? For how long have you planned to use it in practice?
	6. Once you started using the leaflet in clinical practice, have you changed how it was originally planned to be used? If yes, what have you changed and why?
Actual use	7. Are you aware of how the CPNs have been using the leaflet with their patients, e.g. for signposting users to local services available?
	8. Do you think you reached the group(s) of users that you were expecting?
	9. Are you aware of any specific factors that influenced the actual use of the leaflet by the CPNs in clinical practice?
Barriers and facilitators	10. In your opinion, what facilitated and hindered the use of the leaflet in clinical practice?
Impact of the leaflet	11. Has the leaflet changed how the appointments of the CPNs with their patients and families take place? Is this what you were expecting from the dissemination of the leaflet?
	12. Do you think that the leaflet has changed patients' and family carers' perceptions about local care and support available?
	13. Do you think that the leaflet has changed how people with dementia and family carers access and use local services?
Future use	14. Do you plan to keep using the leaflet within your organization? Do you think it should be updated regularly?
	15. Do you think we should change anything about the leaflet (e.g. thinking about its content or its design) or about how it is used in clinical practice?
Other strategies	16. Are you aware of the current strategies or priorities in the area of dementia (e.g. these could be national, local or identified by the organization you work for)? Do you think the leaflet fits the current strategies or priorities?

Co-creation – Process	17. Are you aware that the leaflet was developed with inputs from people living with dementia, their family carers, and several local organizations providing/commissioning dementia care? Did you contribute to the design and development of the leaflet? How?
Co-creation – Impact	18. In your opinion did this approach influence the use of the leaflet in clinical practice? In what way?
Conclusion	19. Do you have any final comment on the leaflet? Or on your experience of using it with your team/service?
	20. Is there anything else that you'd like to add?
Thank you	

Interview guide – Managers from local service providers

Preamble	I'd like to review with you the data you have collected and I will need your help to make sense of them…
Review the activity log	1. Can we have a look at the data covering the period in which the leaflet was used? How do these compare with the previous year?
Pattern of referrals	2. Have you seen a change in the referrals that you received since the dissemination of the leaflet, e.g. in terms of<br>- who the referrers are (e.g. self referral or professionals)<br>- for what services the referrals are made<br>- for whom the referrals are made (i.e. person with dementia or family carers)<br>- when a referral is made (e.g. at a crisis point)
	3. Do you think that the leaflet has changed the perceptions of people with dementia and family carers' about local care and support available?
	4. Do you think that the leaflet has changed how people with dementia and family carers access and use local services?
Future use	5. Do you think we should keep offering the leaflet to people with dementia and family carers in the future?
	6. Do you think we should change anything in how/when/to whom the leaflet is offered?
Other strategies	7. Are you aware of current strategies or priorities in the area of dementia (e.g. these could be national, local or identified by the organization you work for)? Do you think the leaflet meets the priorities of current strategies or priorities?
Co-creation – Process	8. Are you aware that the leaflet was developed with input from both people living with dementia, family carers, and a wide range of local organizations providing or commissioning care and support? If yes, did you contribute to the development of the leaflet? How?
Co-creation – Impact	9. In your opinion did this approach influence whether and how people with dementia and family carers used the leaflet? In what ways?
Conclusion	10. Do you have any final comment on the leaflet? Or on your organisation's experience of using it with people with dementia and their families? Or on your organisation's experience of using it in clinical practice?
	11. Is there anything else that you'd like to add?
Thank you	

De Poli C, *et al. BMJ Open* 2020; 10:e038397. doi: 10.1136/bmjopen-2020-038397

Focus group guide – Leaflet working group

Preamble	I'd like to share with you the feedback about the leaflet that we collected from different perspectives (users, implementers, local providers) and invite you to discuss them …
Feedback from people living with dementia and family carers	1. These are some extracts from interviews carried out with a group of people living with dementia and family carers….
Feedback from implementers (CPNs, team and service managers)	2. These are some extracts from interviews carried out with a group of CPNs and with the team and service managers of the CMHSOP…
Feedback from third sector organizations	3. These are some data about the trends in referrals into these organizations…
	4. These are some extracts from the interviews carried out with the local providers…
Co-creation – Impact	5. As you are aware, the leaflet was developed collaboratively, with input from different stakeholders. In your opinion did this approach influence whether and how people with dementia and family carers used the leaflet? In what ways?
	6. Similarly, do you think that this approach has had an impact on the use of the leaflet by CPNs?
Updating the leaflet	7. This is the feedback we collected about the leaflet, with some suggestions for improvement…
	8. Do you have any other suggestions for improvement?
	9. Do you have any suggestions for improving the process of dissemination of the leaflet?
	10. Do you intend to update the leaflet and make it available to people living with dementia and family carers? How do you plan to go about this?
	11. Is there anything else you would like to add?
Thank you	

De Poli C, *et al. BMJ Open* 2020; 10:e038397. doi: 10.1136/bmjopen-2020-038397
