# Supplementary material for: Effectiveness–implementation hybrid type 2 study evaluating an intervention to support ‘information work’ in dementia care: an implementation study protocol
Source: BMJ Open. 2020 Dec 8;10(12):e038397. doi: 10.1136/bmjopen-2020-038397 (PMC7725094; doi:10.1136/bmjopen-2020-038397)
Supplement: Author's manuscript [file bmjopen-2020-038397.draft_revisions.pdf]

BMJ Open

BMJ Open is committed to open peer review. As part of this commitment we make the peer review history of every article we publish publicly available.

When an article is published we post the peer reviewers' comments and the authors' responses online. We also post the versions of the paper that were used during peer review. These are the versions that the peer review comments apply to.

The versions of the paper that follow are the versions that were submitted during the peer review process. They are not the versions of record or the final published versions. They should not be cited or distributed as the published version of this manuscript.

BMJ Open is an open access journal and the full, final, typeset and author-corrected version of record of the manuscript is available on our site with no access controls, subscription charges or pay-per-view fees (http://bmjopen.bmj.com).

If you have any questions on BMJ Open's open peer review process please email info.bmjopen@bmj.com

BMJ Open

# Effectiveness-implementation hybrid type 2 study evaluating an intervention to support 'information work' in dementia care: implementation study protocol

| | |
|---|---|
| Journal: | *BMJ Open* |
| Manuscript ID | bmjopen-2020-038397 |
| Article Type: | Protocol |
| Date Submitted by the Author: | 09-Mar-2020 |
| Complete List of Authors: | De Poli, Chiara; London School of Economics and Political Science, Management<br>Oyebode, Jan; University of Bradford, School of Dementia Studies<br>Binns, Christopher; Tees, Esk and Wear Valleys NHS Trust<br>Glover, Richard; NHS North of England Commissioning Support Unit<br>Airoldi, Mara; University of Oxford, Blavatnik School of Government |
| Keywords: | Dementia < NEUROLOGY, Quality in health care < HEALTH SERVICES ADMINISTRATION & MANAGEMENT, QUALITATIVE RESEARCH |
| | |

SCHOLARONE™
Manuscripts

*I, the Submitting Author has the right to grant and does grant on behalf of all authors of the Work (as defined in the below author licence), an exclusive licence and/or a non-exclusive licence for contributions from authors who are: i) UK Crown employees; ii) where BMJ has agreed a CC-BY licence shall apply, and/or iii) in accordance with the terms applicable for US Federal Government officers or employees acting as part of their official duties; on a worldwide, perpetual, irrevocable, royalty-free basis to BMJ Publishing Group Ltd ("BMJ") its licensees and where the relevant Journal is co-owned by BMJ to the co-owners of the Journal, to publish the Work in this journal and any other BMJ products and to exploit all rights, as set out in our licence.*

*The Submitting Author accepts and understands that any supply made under these terms is made by BMJ to the Submitting Author unless you are acting as an employee on behalf of your employer or a postgraduate student of an affiliated institution which is paying any applicable article publishing charge ("APC") for Open Access articles. Where the Submitting Author wishes to make the Work available on an Open Access basis (and intends to pay the relevant APC), the terms of reuse of such Open Access shall be governed by a Creative Commons licence – details of these licences and which Creative Commons licence will apply to this Work are set out in our licence referred to above.*

*Other than as permitted in any relevant BMJ Author's Self Archiving Policies, I confirm this Work has not been accepted for publication elsewhere, is not being considered for publication elsewhere and does not duplicate material already published. I confirm all authors consent to publication of this Work and authorise the granting of this licence.*

For peer review only

# Effectiveness-implementation hybrid type 2 study evaluating an intervention to support 'information work' in dementia care: implementation study protocol

Chiara De Poli [a]
Jan Oyebode [b]
Chris Binns [c]
Richard Glover [d]
Mara Airoldi [e]

[a] Corresponding author. Department of Management, London School of Economics and Political Science, Houghton Street, WC2A 2A London, UK, c.de-poli@lse.ac.uk, phone +44 (0)2079556749

[b] Centre for Applied Dementia Studies, Faculty of Health Studies, University of Bradford, Bradford, UK

[c] Tees, Esk and Wear Valleys NHS Trust, Durham, UK

[d] NHS North of England Commissioning Support, Durham, UK

[e] Blavatnik School of Government, University of Oxford, Oxford, UK

Word count: 3,703

For peer review only



For peer review only - http://bmjopen.bmj.com/site/about/guidelines.xhtml

## ABSTRACT

### Introduction

Patients with long-term conditions consistently report a lack of information around services and support available to them. This unmet need for information is significant among people with dementia and family carers. A quality improvement intervention is being carried out to tackle this issue as part of a co-creation initiative in the North East of England (UK). The intervention consists of the dissemination (via the local community mental health services for older people) of a leaflet about services available to people with dementia and their family carers in the study site. This protocol is reported in accordance with the Standards for Reporting Implementation Studies (StaRI).

### Methods and analysis

This effectiveness-implementation hybrid type 2 study aims at understanding (1) the unfolding and outcomes of the implementation strategy, (2) perceived outcomes of the intervention (for people with dementia and family carers, staff implementing the intervention, local service providers), (3) the contribution of co-creation to the design and implementation of the intervention and its outcomes. The prospective theory of change of the intervention articulated by local stakeholders is used as a reference framework against which to assess the implementation and outcomes of the intervention. Evaluation data will be collected through in-depth interviews with people with dementia and family carers receiving the intervention, staff implementing the intervention, managers from local service providers. Activity data from local service providers will be collected to triangulate the interview data. A focus group with key stakeholders will support the sense-making of findings. The realist configuration of mechanism – context – outcome is adopted to analyse and interpret the data.

### Ethics and dissemination

Ethical and research governance approvals have been obtained from the West Midlands - South Birmingham Research Ethics Committee. The results of the study will be submitted for publication in peer-reviewed journals and disseminated through conferences.


For peer review only



For peer review only - http://bmjopen.bmj.com/site/about/guidelines.xhtml

**Strengths and limitations of this study**

- The study is reported in accordance with the Standards for Reporting Implementation Studies (StaRI) framework complemented by additional relevant reporting standards, such as the Template for intervention description and replication (TIDieR) checklist and Proctor et al.'s framework for specifying and reporting implementation strategies
- The evaluation framework integrates the evaluation of the implementation strategy and of the implemented intervention both in terms of process and of outcomes
- The mixed-method evaluation uses data collected through in-depth interviews with three sets of stakeholders, services' activity data and a focus group
- In-depth, longitudinal interviews with people with dementia are planned to explore their experiences of receiving and using the leaflet, however recruitment can be difficult and dropout rate can be high
- The evaluation will provide insights regarding the process and impact of delivering and receiving the intervention but will have no comparison condition

For peer review only

## INTRODUCTION

Patient activation, self-management, ageing in place, enablement and shared-decision making are recurrent themes in policy discourses across health systems addressing ageing populations, chronic diseases, multi-morbidity and complex needs.

Underpinning, albeit often implicit, assumptions of such policy discourses is the idea that by providing information, patients and family carers are supported to make better choices, have more agency on managing their own health (or the health of the person they care for), may feel empowered and ultimately may report better outcomes and satisfaction with the care and support they receive.

However, such a view fails to acknowledge the 'information work', i.e. the process of seeking, using, evaluating and sharing of information[1] that is required from patients and family carers in order for them to be able to make better choices or to play a more active role in their own or others' care.[2] Also, it assumes patients and family carers are physically and cognitively able to carry out information work, and it overlooks the emotional processes that influence adaptation to long-term conditions, which may influence whether information work is successfully undertaken.

Extant research consistently conveys unmet needs for information as expressed by patients and family carers.[3–5] This issue has been recognised as highly significant among people with dementia and family carers,[6–10] whose information needs span beyond the topics usually addressed by information materials available, leaving them with some information gaps.[11,12] Moreover, people living with dementia identify a preference to receive information directly from health and social care professionals rather than over the internet, which is the primary source of information to which they are often signposted.[13] They highlight that the timeliness of their information needs, which vary along the dementia trajectory,[14] is seldom taken into account by information providers and resources. They also expect up-to-date information to be provided in a culturally acceptable way and in easy to understand language.[15–17]

Building on this research, an implementation study is being carried out as part of a co-creation initiative in dementia care taking place in the North East of England (UK). The study aims at designing, implementing and evaluating a leaflet about local services providing care and support to people with dementia and their family carers living in the study site.

This article reports the protocol for the evaluation of the implementation study in accordance with the Standards for Reporting Implementation Studies (StaRI) framework and adopts the recommended dual strands for reporting, one about the intervention being implemented and the other about its implementation strategy (i.e. the 'bundle' of techniques used to enhance the adoption, integration into routine practice and sustainability of the intervention).[18,19]

The evaluation has three aims. First, it intends to review the unfolding and outcomes of the implementation strategy. Secondly, it aims to assess the outcomes of the intervention as perceived by people living with dementia and their family carers, staff implementing the intervention and local organizations providing care and support to people with dementia and family carers and to explain how and why the intervention contributed to achieve these outcomes. Thirdly, it will reflect on the extent to which, how and why the co-creation initiative has influenced the design, implementation and impact of the intervention.

The article is organised as follows. The next section provides an overview of the process that supported the development of the intervention and describes the intervention itself and its implementation strategy. The subsequent section articulates the evaluation framework, the



For peer review only - http://bmjopen.bmj.com/site/about/guidelines.xhtml

analytical framework, the methods for data collection and analysis and the study samples. Ethical considerations relevant for the study and dissemination activities are covered in the last section of the protocol.

## INTERVENTION DEVELOPMENT

The implementation study herein described has been developed as part of a co-creation initiative in dementia care taking place in the North East of England (UK). The initiative, started in 2015 and with expected completion at the end of 2020, involves a constellation of local stakeholders, including people living with dementia and their family carers, health and care professionals, service managers and commissioners alongside a research team, encompassing university-based researchers and a local research facilitator.

Grounded in the principles of action research, co-creation activities were organised in three broad phases (Figure 1).[20] The diagnostic phase (phase 1, 2015-2018) mapped the local dementia care system and identified its strengths and weaknesses. The improvement phase (phase 2, 2017-2018) articulated and designed three interventions to address the priority issues identified in the diagnostic phase. Each improvement area was addressed via a sub-cycle of action research. Local stakeholders with an investment in the respective improvement areas were invited to work with the research team to identify different available options to address the improvement areas, select a course of action and accordingly design an intervention and define its implementation.

The planned evaluation (phase 3, 2020) will focus on both the improvement interventions and the co-creation initiative. With respect to the first, the evaluation aims to assess the outcomes of the implementation strategy and the outcomes of each intervention. With respect to the latter, the evaluation aims to unpack how the co-creation initiative has unfolded in practice and its impact on local policy-making around care and support for people with dementia.

PLEASE INSERT FIGURE 1 ABOUT HERE

### *The intervention and the implementation strategy*

A fundamental unmet need for information was reported by people with dementia and family carers who took part in the diagnostic phase. Local stakeholders agreed to tackle this issue by providing up-to-date, accessible and timely information about local services available to those living with dementia and family carers. Figure 2 provides a timeline of the process underpinning the development of the agreed intervention.

PLEASE INSERT FIGURE 2 ABOUT HERE

A local working group (including commissioners, statutory service providers, third sector organisations providing support and care to people with dementia and to family carers, a professional designer, alongside the research team) was set up with the remit of developing an information leaflet and planning its implementation. The group produced three prototypes which were shared and discussed in a consultation exercise involving about 20 among people with dementia and family carers. The preferred prototype was iteratively refined using best practice guidance on Patient Information Leaflets[21] and taking into account feedback



For peer review only - http://bmjopen.bmj.com/site/about/guidelines.xhtml

provided by its potential users, commissioners and service providers. In its final version, the leaflet presents services clustered around four main themes (“I want to speak to someone for advice and support”, “I want to live safely and with the right support”, “I want to know what to do in a crisis”, “Where can I go for more information?”) and it can be printed out on a two-sided A4 using a high contrast colour combination.

Subsequently, the working group agreed a phased implementation process: the Community Mental Health Services for Older People (CMHSOP) within the local mental health trust were identified as the first implementation route because of their key role in diagnosing dementia and in the resulting post-diagnostic care planning. Following diagnosis and initial care planning, those whose needs are stable are discharged to primary care while those with more complex needs continue to receive secondary services.

The working group preliminarily explored with the CMHSOP service manager, team managers and representatives of the Community Psychiatric Nurses (CPNs) how the leaflet could be used in clinical practice. In January 2019, following positive and supportive feedback, the leaflet working group members and the CMHSOP service manager, team managers and two CPNs took part in a focus group aimed at articulating the theory of change associated with the intervention itself. This exercise helped making explicit the expected process of implementing the intervention in practice and its anticipated impact. Mobilisation activities (e.g. information sessions within team meetings and regular reminders emailed to team managers and cascaded to CPNs) were planned and carried out to inform, and subsequently to remind, all CPNs about the leaflet and its use in practice. In June 2019, all the CPNs across the four locality teams covering the study site were invited to start using the leaflet in their clinical practice.

CPNs agreed to hand out the leaflet to patients on their caseload who received a diagnosis of any type of dementia and/or to their family carers. CPNs were expected to use their clinical judgement in deciding to whom and when to offer the leaflet, dependent upon the patient’s readiness to receive the leaflet and their individual and family circumstances. They expected to hand it out during their encounter with patients and families, when they felt that the leaflet would support their conversations with them. For these reasons, the use of the leaflet was not embedded at a specific point of the clinical pathway.

A detailed description of the intervention and its implementation strategy are outlined in accordance with the StaRI framework respectively in Table 1 and Table 2 (item 9).



For peer review only - http://bmjopen.bmj.com/site/about/guidelines.xhtml

**Table 1 – Overview of the implementation strategy reported in accordance with the StaRI checklist** [22]

| **IMPLEMENTATION STRATEGY (i.e. how the intervention was implemented)** | | |
|---|---|---|
| **Item** | **Description and reference to methodological frameworks and reporting standards** | |
| 1. Title | | Effectiveness-implementation hybrid type 2 study evaluating an intervention to support 'information work' in dementia care |
| 2. Abstract | | See Abstract of the protocol |
| 3. Introduction | To identify the problem | See Introduction of the protocol |
| 4. Introduction | Rationale for the implementation strategy and the intervention | See Intervention development of the protocol |
| 5. Aims and objectives | | 1. To assess the unfolding of the implementation strategy and its outcomes<br>2. To assess the outcomes of the intervention as perceived by people living with dementia and their family carers, staff implementing the intervention and local organizations and to explain how and why the intervention contributed to achieve these outcomes<br>3. To reflect on the extent to which, how and why the co-creation initiative has influenced the design, implementation and impact of the intervention |
| 6. The design and key features of the evaluation | | The evaluation of the implementation strategy aims at understanding the feasibility of the intervention by investigating how the actual implementation process has unfolded in practice and identifying any discrepancies between the planned and the actual implementation process |
| 7. Context | Context in which the intervention is implemented | Community mental health services for older people (CMHSOP) within a mental health foundation trust in the North East of England (UK)<br>- North East of England is an under-researched area compared to other parts of England<br>- Local health economy with average recorded diagnosed dementia prevalence (for 2018-2019, local recorded prevalence rate 0.9% vs national recorded prevalence rate 0.8%) and average recorded annual review rate (for 2018-2019, local annual review rate 79.2% vs national recorded prevalence rate 77.9%)[23]<br>- Mental health foundation trust, established in 2006 providing a range of mental health, learning disability and eating disorder services for around two million people across a large and diverse geographical area<br>- CMHSOP provide community-based services for older people experiencing cognitive impairment and mental health problems. Organized around four localities, each locality team includes community psychiatric nurses (CPNs), occupational therapists, physiotherapists, psychologists, support workers to provide multi-disciplinary support |



For peer review only - http://bmjopen.bmj.com/site/about/guidelines.xhtml

<table>
<tr><th colspan="3">IMPLEMENTATION STRATEGY (i.e. how the intervention was implemented)</th></tr>
<tr><th>Item</th><th>Description and reference to methodological frameworks and reporting standards</th><th></th></tr>
<tr><td></td><td></td><td>Intervention was developed in the context of a 5-year action research co-creation initiative funded by an English charity, involving researchers from three English universities and a research facilitator from the local commissioning support unit alongside a constellation of local stakeholders</td></tr>
<tr><td>8. Target sites</td><td></td><td>Four locality teams, staffing levels and caseload are reported in the table below
<table>
<tr><th>Team</th><th>Total staff</th><th>CPNs</th><th>Caseload</th></tr>
<tr><td>Team 1</td><td>48</td><td>9</td><td>1,368</td></tr>
<tr><td>Team 2</td><td>43</td><td>8</td><td>1,377</td></tr>
<tr><td>Team 3</td><td>27</td><td>5</td><td>787</td></tr>
<tr><td>Team 4</td><td>26</td><td>5</td><td>791</td></tr>
</table></td></tr>
<tr><td>9. Implementation strategy</td><td>Description of the implementation strategy in accordance with Proctor et al.'s framework for reporting implementation strategies[24]</td><td>Name and definition of the implementation strategy: bundle of professional and material interventions aiming at the development, adoption and use of the dementia leaflet among CPNs (based on framework by [25,26]<br><br>Specification of the implementation strategy<br>- Actors: Community Psychiatric Nurses (CPNs) and team managers and service manager across four CMHSOP teams<br>- Actions: Described in accordance with relevant phase of the Plan-Do-Study-Act (PDSA) framework<br>  - Plan:<br>    Professional interventions<br>    o Established a local working group in charge of overseeing the development the leaflet and its implementation strategy<br>    o Iteratively developed a dementia leaflet, embedding feedback from people with dementia, family carers, professionals from local organisations providing care and support to people with dementia and family carers<br>    o Identified the implementation strategy via CPNs based in CMHSOP, identified local champion (service manager), identified implementation sites</td></tr>
</table>



For peer review only - http://bmjopen.bmj.com/site/about/guidelines.xhtml

| IMPLEMENTATION STRATEGY (i.e. how the intervention was implemented) | | |
|---|---|---|
| **Item** | **Description and reference to methodological frameworks and reporting standards** | |
| | | o Organised mobilisation activities with implementation sites, including information sessions at team meetings and email reminders<br>Material intervention<br>o Printed copies of the leaflet made available to implementers<br>- Do: CPNs offer a copy of the leaflet to people with dementia or their family carers attending an appointment (any type)<br>- *Action targets*: People living with dementia (any type of dementia, diagnosed at any time) and their family carers attending an appointment with their CPNs. CPNs were expected to use their clinical judgment in identifying patients or family members deemed to be ready to receive the leaflet<br>- *Temporality*: The leaflet can be offered at any point during the period in which the patient was under the care of the CMHSOP (at initial assessment, at post diagnosis, at reviews, at discharge)<br>- *Dose:* Depending on the individual readiness to receive the leaflet and individual and family circumstance, CPNs decide whether to offer or not the leaflet as part of their appointment with the patient. The leaflet could be offered more than once at different points in time |
| 10. Sub-groups | Any subgroups recruited for additional research tasks, and/or nested studies | Not applicable to this study |
| **Evaluation** | | |
| 11. Outcomes | Using Proctor et al.'s taxonomy of outcomes for implementation research [27] | - Acceptability of the intervention by CPNs: perception among CPNs that the intervention is agreeable, palatable, or satisfactory<br>- Adoption of the intervention by CPNs: CPNs' intention, initial decision, or action to try the intervention<br>- Appropriateness of the intervention implemented as judged by CPNs: perceived fit, relevance, or compatibility of the intervention for the setting of CMHSOP and perceived fit of the intervention to address the issue<br>- Cost of the intervention for the implementers<br>- Feasibility of the implementation of the intervention: the extent to which the intervention can be successfully used or carried out within the setting of the CMHSOP<br>- Penetration of the intervention: integration of the intervention within the CMHSOP<br>- Sustainability of the intervention: extent to which the intervention is maintained or institutionalised within the CMHSOP |



For peer review only - http://bmjopen.bmj.com/site/about/guidelines.xhtml

| IMPLEMENTATION STRATEGY (i.e. how the intervention was implemented) | | |
|---|---|---|
| **Item** | **Description and reference to methodological frameworks and reporting standards** | |
| 12. Process evaluation objectives and outcomes related to the mechanism(s) through which the strategy is expected to work | Objectives and outcomes related to the mechanism by which the strategy is expected to work, using MRC framework for complex interventions [28] | - Fidelity of the intervention: adherence to the intervention protocol<br>- Adaptations of the intervention<br>- Dose: the quantity of intervention implemented<br>- Reach: whether the intended audience comes into contact with the intervention, and how |
| 13. Data collection methods | | In-depth interviews with CPNs, team managers and service manager of CMHSOP |
| 14. Sample size | | - Up to 12 CPNs from four locality teams<br>- Four team managers<br>- One service manager |
| 15. Methods of analysis | | Thematic content analysis of interview data |



For peer review only - http://bmjopen.bmj.com/site/about/guidelines.xhtml

**Table 2 – Overview of the intervention reported in accordance with the StaRI checklist** [22]

| **INTERVENTION (i.e. the actual intervention being implemented)** | | |
|---|---|---|
| **Item** | **Description and reference to methodological frameworks and reporting standards** | |
| 1. Title | | Effectiveness-implementation hybrid type 2 study evaluating an intervention to support 'information work' in dementia care |
| 2. Abstract | | See Abstract of the protocol |
| 3. Introduction | To identify the problem | See Introduction of the protocol |
| 4. Introduction | Rationale for the implementation strategy and the intervention | See Intervention development of the protocol |
| 5. Aims and objectives | | 1. To assess the unfolding of the implementation strategy and its outcomes<br>2. To assess the outcomes of the intervention as perceived by people living with dementia and their family carers, staff implementing the intervention and local organizations and to explain how and why the intervention contributed to achieve these outcomes<br>3. To reflect on the extent to which, how and why the co-creation initiative has influenced the design, implementation and impact of the intervention |
| 6. The design and key features of the evaluation | | The evaluation of the intervention aims at assessing the outcomes of the intervention with respect to people with dementia and family carers (primary outcomes), to the implementers of the intervention (proximal outcomes) and to local service providers (distal outcomes) |
| 7. Context | Context in which the intervention was implemented | As per implementation strategy described above |
| 8. Target population | Characteristics of the population targeted by the intervention and eligibility criteria | People living with dementia (any type of dementia, diagnosed at any time) and their family carers attending an appointment with their CPNs. CPNs were expected to use their clinical judgment in identifying patients or family members deemed to be ready to receive the leaflet |
| 9. Intervention | Description of the intervention, provided in accordance with the TIDieR checklist [29] | Name: Leaflet about local care and support available to people living with dementia and family carers<br><br>Why: in the diagnostic phase of the co-creation initiative, people living with dementia and family carers consistently reported a lack of timely, up-to-date information provided in a user-friendly format which hinders their 'information work'. The intervention has been designed to provide a short, up-to-date, paper-based source of information that could |



For peer review only - http://bmjopen.bmj.com/site/about/guidelines.xhtml

| INTERVENTION (i.e. the actual intervention being implemented) | | |
|---|---|---|
| **Item** | **Description and reference to methodological frameworks and reporting standards** | |
| | | be handed out and used by CPNs in their encounter with people with dementia and their family carers and support their 'information work'<br><br>What (materials): An A4 three-folded, two-sided leaflet printed in high contrast colour combination. Information are organized in four themes, for which relevant services, a short description and contact details are provided<br><br>What (procedures): CPNs offer the leaflet to patients and their family carers as part of their clinical encounters<br><br>Who: 27 qualified mental health nurses (CPNs) part of the organic cells working across four locality team of the local CMHSOP who assess, diagnose, review medications and offer post-diagnostic support to people with dementia and signpost family carers to services available to them<br><br>How: the leaflet is offered in the context of the appointment attended by the person with dementia and their family carer. The CPN can offer the leaflet in response to a current specific need of the person with dementia or of the family carer (either explicitly reported or latent) or opportunistically, to make them aware of local services which could be of interest either at the present time or in the future<br><br>Where: the intervention takes place in the outpatient clinics of the CMHSOP services of the local mental health foundation trust<br><br>When and how much: CPNs can offer the leaflet at any point in the period when the patient is under the care of the CMHSOP (e.g. at initial assessment, at diagnosis, in the post diagnostic appointment, at review, upon transfer of care back to GP). It could be offered by the CPNs more than once at different points in time<br><br>Tailoring: CPNs are expected to use their clinical judgement in deciding whether, when, how and to whom to offer the leaflet based on<br>- patients or families' readiness to accept the leaflet (e.g. some patients do not temporarily - or will never - accept a diagnosis of dementia)<br>- length of the appointments (e.g. initial assessment can take up to 90 minutes and patients and families may be overloaded with information) |



For peer review only - http://bmjopen.bmj.com/site/about/guidelines.xhtml

| INTERVENTION (i.e. the actual intervention being implemented) | | |
|---|---|---|
| **Item** | **Description and reference to methodological frameworks and reporting standards** | |
| | | - type of appointments (e.g. titration appointments may cause disappointment in patients) |
| 10. Sub-groups | Any subgroups recruited for additional research tasks, and/or nested studies | Not applicable to this study |
| **Evaluation** | | |
| 11. Outcomes | Primary and other outcomes of the intervention | For people with dementia and family carers (primary outcomes)<br>- their satisfaction with the availability of information on local services (e.g. in terms of timing and quality of information)<br>- their awareness of local services<br>- in the longer term, their access and use of local services<br><br>For implementers of the intervention (proximal outcomes)<br>- CPN, team and service managers' awareness of local services and support available to people diagnosed with dementia and to their family carers<br>- CPN, team and service managers' confidence to hold conversations around using services available to those living with dementia and their family carers<br><br>For local service providers (distal outcomes)<br>- volume of referrals into their services from people with dementia and their family carers<br>- appropriateness of the use of services among those living with dementia and family carers |
| 12. Process evaluation objectives and outcomes related to the mechanism(s) through which the strategy is expected to work | Objectives and outcomes related to the mechanism by which the strategy is expected to work, using MRC framework for complex interventions [28] | As per implementation strategy |
| 13. Data collection methods | | People with dementia and family carers<br>- Longitudinal semi-structured in-depth interviews: interview 1 carried out 4-6 weeks after the appointment with the CPN when they receive the leaflet; interview 2 (follow-up) scheduled about 5 months after interview 1<br><br>Implementers of the intervention |



For peer review only - http://bmjopen.bmj.com/site/about/guidelines.xhtml

| INTERVENTION (i.e. the actual intervention being implemented) | | |
|---|---|---|
| **Item** | **Description and reference to methodological frameworks and reporting standards** | |
| | | - Semi-structured in-depth interviews with CPNs, team managers and service manager of CMHSOP<br><br>Local service providers<br>- Activity log recording activity data for 24 months (12 months before/after the implementation of the intervention)<br>- Semi-structured in-depth interviews with managers<br><br>Local working group<br>- focus group with about 12 participants (including commissioners, statutory service providers, staff from CMHSOP, managers from third sector organizations, a person with dementia and a family carer) |
| 14. Sample size | | People living with dementia and family carers<br>- Up to 30 people living with dementia (any type of dementia, diagnosed at any time, willing and able to consent) and family carers (of somebody with any type of dementia, any degree of severity, diagnosed at any time)<br><br>Implementers of the intervention<br>- Up to 12 CPNs from 4 locality teams<br>- Four team managers<br>- Service manager<br><br>Local service providers<br>- managers from up to five local organisations providing care and support to people living with dementia and their family carers<br><br>Local working group<br>- about 12 participants attending the focus group (including commissioners, statutory service providers, staff from CMHSOP, managers from third sector organizations, a person with dementia and a family carer) |
| 15. Methods of analysis | | Thematic content analysis of interview and focus group data<br>Descriptive statistics for activity data for local service providers for triangulation purposes |



For peer review only - http://bmjopen.bmj.com/site/about/guidelines.xhtml

# METHODS AND ANALYSIS

### *Evaluation framework*

The study adopts an effectiveness-implementation hybrid type 2 design which allows simultaneous evaluation of the intervention and its implementation strategy,[30] as reflected in the evaluation framework (Figure 3).

PLEASE INSERT FIGURE 3 ABOUT HERE

The evaluation framework maps the phases of the study (design – implementation – evaluation) on to the key components of the intervention and shows the relations among them. In the design phase, the intervention and the implementation strategy (i.e. the bundle of professional and material interventions intended to sustain the adoption and use of the leaflet among CPNs) were articulated with the theory of change focus group. Following the implementation, the evaluation will

1. assess the unfolding of the implementation strategy and its outcomes
2. assess the outcomes of the intervention as perceived by people with dementia and family carers, the implementers of the intervention, as well as the local services available to people with dementia and their families
3. identify the mechanisms of action, i.e. the "active ingredients" that ignited the implementation strategy and the intervention and hence contributed to the observed outcomes
4. reflect on the contribution of context (e.g. the social, economic, policy and organisational factors surrounding the intervention, including, but not limited to, the co-creation initiative, as described in Table 1, item 7) to the design and implementation of the intervention and its implementation strategy and relative outcomes

Outcomes of the implementation strategy

The implementation strategy is evaluated with respect to both its outcomes and its process dimension, as per the StaRI framework. Following Proctor et al.'s taxonomy[31], the following outcomes will be assessed with respect to the implementation strategy (Table 1, item 11):

- Acceptability of the intervention by CPNs: perception among CPNs that the intervention is agreeable, palatable, or satisfactory
- Adoption of the intervention by CPNs: CPNs' intention, initial decision, or action of trying out the intervention
- Appropriateness of the intervention implemented as judged by CPNs: perceived fit, relevance, or compatibility of the intervention for the setting of the CMHSOP and perceived fit of the intervention to address the issue
- Cost of the intervention for the implementers
- Feasibility of the implementation of the intervention: the extent to which the intervention can be successfully used or carried out within the setting of the CMHSOP



For peer review only - http://bmjopen.bmj.com/site/about/guidelines.xhtml

- Penetration of the intervention: the extent to which the intervention is integrated into the practice of the CMHSOP
- Sustainability of the intervention: extent to which the intervention is maintained or embedded within the CMHSOP

Outcomes of the intervention

The theory of change exercise run in January 2019 highlighted that the intervention was expected to have an impact on (Table 2, item 11):

- Its main target population, i.e. people with dementia and family carers (direct outcomes)
- The implementers of the intervention, i.e. CPNs, team and service managers of the CMHSOP delivering the intervention (proximal outcomes)
- Local providers of care and support to people with dementia and their families, such as local third sector organisations (distal outcomes)

With respect to people with dementia and family carers, the intervention was expected to improve three outcomes directly associated with the intervention: (i) their satisfaction with the availability of information on local services (e.g. in terms of timing and quality of information), (ii) their awareness of local services and (iii) in the longer term, their access and use of local services.

With respect to the implementers, the intervention was expected to improve two proximal outcomes identified for the intervention: (i) CPN, team and service manager's awareness of local services and support available to people diagnosed with dementia and to their family carers and (ii) CPN, team and service manager's confidence to hold conversations around using services available to those living with dementia and their family carers.

With respect to local service providers, the intervention was expected to impact on two distal outcomes: (i) increase in the volume of referrals into their services from people with dementia and their family carers and (ii) improvement in appropriateness of the use of services among those living with dementia and family carers.

Process evaluation

The MRC framework for process evaluation[28] is used to investigate (Table 1 and Table 2, item 12)

- the actual implementation strategy, in terms of implementation fidelity (or, conversely adaptation), reach and dose,
- the mechanisms of action of the implementation strategy and of the intervention which brought about change

The process evaluation will highlight how the implementation process has unfolded in practice and any discrepancies between the actual implementation process and how it was articulated in the theory of change. Moreover, it will help to surface how and why the intervention and its implementation strategy have contributed to achieve these outcomes.



For peer review only - http://bmjopen.bmj.com/site/about/guidelines.xhtml

### *Analytical framework*

The framework underpinning the realist evaluation will be adopted as analytical tool. Realist evaluation acknowledges that interventions work (have successful outcomes) in so far as they introduce appropriate ideas and opportunities (mechanisms) to groups of stakeholders in the appropriate social and cultural conditions (context), as condensed in the following formula [32]

mechanism + context = outcome

According to the realist framework, mechanisms are the underlying processes or hidden causal levers of intervention activities that make them work[33] and are represented by the psychological or social explanations of behaviour of intervention participants and their ideas about how change is achieved.[34] Context refers to the conditions likely to enable or constrain the activation of interventions' mechanisms (e.g. the organisational setting or the broader policy and institutional environment in which an intervention is embedded). Outcome is the anticipated and unanticipated consequence that is brought about by the interaction of different interventions' mechanisms in different contexts.

### *Data collection methods and samples*

The mixed-method evaluation will build on data collected from four sets of stakeholders involved in the intervention (Table 1 and Table 2, items 13 and14):

- people living with dementia and family carers who are at the immediate receiving end of the intervention
- CPNs, team and service managers based in the CMHSOP who are implementing the intervention
- local providers of care and support to people with dementia and their families, as the organisations expected to observe a change in their service access and use as a consequence of the intervention
- local working group, who contributed to the development of the intervention

Figure 4 provides an overview of the data collection timeline.

PLEASE INSERT FIGURE 4 ABOUT HERE

People with dementia and family carers

Semi-structured in-depth interviews with up to 30 people living with dementia (any type of dementia, diagnosed at any time, willing and able to consent) and family carers (of somebody with any type of dementia, any degree of severity, diagnosed at any time) will be carried out at two points in time: interview 1 will be carried out 4-6 weeks after the appointment with the CPN when they receive the leaflet and interview 2 (follow up) will be scheduled about 5 months after interview 1.



For peer review only - http://bmjopen.bmj.com/site/about/guidelines.xhtml

The two timepoints have been established to best capture participants' experiences of receiving (interview 1) and using (follow up interview) the leaflet. Accordingly, interview 1 will aim to understand (i) if, when and how they received the leaflet; (ii) how they anticipate using the leaflet; (iii) perceptions about the leaflet. Interview 2 will aim to understand (i) whether, how, when and why they used (or not) the leaflet; (ii) whether the leaflet has changed their care seeking behaviour; (iii) overall satisfaction with the leaflet.

Longitudinal interviews with people living with dementia and their family carers are a well-established research method.[35–39] The follow up period is appropriate for research purposes. Over a period of 6 months, personal and family circumstances can change and this may prompt those living with dementia and their family carers to seek help and support. Similarly, those recently diagnosed may not feel immediately ready to get in touch with local services but this could change after 6 months.

This purposeful sample will be recruited when they attend their first appointment with the CPN. The size of the sample is expected to allow to capture a wide range of perspectives (varying for roles, i.e. people with dementia vs family carers, types of dementia diagnosis, levels of cognitive impairment, age, gender and living arrangements) while achieving data saturation. The recruitment will be carried out on a rolling basis alongside the implementation of the intervention and will stop when the desired sample size is reached.

Community Mental Health Services for Older People (CMHSOP)

In-depth interviews with CPNs, team and service managers from the implementing CMHSOP will be carried out about 6 months from the start of dissemination of the leaflet. Up to 20 staff members will be recruited across the 4 localities in which the CMHSOP operate.

The interviews aim to understand (i) whether, when and how they used (or not) the leaflet in clinical practice; (ii) whether the leaflet has changed any aspect of their clinical practice; (iii) the impact of adopting the principles of co-creation on the implementation and impact of the intervention.

Local service providers

Activity data from up to five local organisations listed on the leaflet which provide care and support to people living with dementia and their family carers will be collected for a period of 24 months (from 12 months before the implementation, for an implementation period of 12 months). Semi-structured in-depth interviews with service managers will be undertaken (i) to review the activity data and (ii) to understand whether they have observed a change in pattern of referral to services linked to the dissemination of the leaflet.

Local working group

A focus group with the local working group (about 12 participants including commissioners, statutory service providers, staff from CMHSOP, managers from third sector organizations, a person with dementia and a family carer) will be held following preliminary analysis of the evaluation data. This will aim to

- discuss the emerging findings about the evaluation of the implementation strategy



For peer review only - http://bmjopen.bmj.com/site/about/guidelines.xhtml

- discuss the emerging findings around the impact of the intervention on people with dementia and their families, CPNs and mental health services and local third sector organisations
- reflect on the impact of co-creation initiative on the implementation and impact of the intervention

### *Informed consent*

Written and verbal versions of the Participant Information Sheet and Informed Consent Sheet will be presented to participants. The Participant Information Sheet details the nature of the study, what participation will involve, any risks involved. It will be clearly stated that the participant is free to withdraw at any time, for any reason without impact on future care or service access, and with no obligation to give the reason for withdrawal. Participants will be allowed as much time as they wish to consider the information (at least 48 hours), and will have the opportunity to ask questions of the Investigator, their GP and/or CPNs (for people with dementia and family carers), or other independent parties in order to decide whether they will participate in the study. Written informed consent will be obtained.

Maximum efforts will be devoted to recruit people living with dementia into this study. Historically, people with dementia have been excluded from much research in social and health care on the basis that their declining cognitive abilities may affect capacity to consent.[40] However, the ability of many people with dementia to provide informed consent, based on their ability to understand information, evaluate risks and benefits, and communicate decisions effectively has been established.[41,42] Prior to the interview, an informal conversation about the research will allow the researchers to ascertain that the potential participant has capacity to consent. If the researcher believes that the person with dementia has capacity, she will take written informed consent and will start the interview.

### *Data analysis*

The interviews will be transcribed, anonymised and analysed using thematic analysis (performed with NVivo12) (Table 1 and Table 2, item 15).[43] Following the realist approach, the initial codebook will be developed around the themes (codes) of context conditions, underlying mechanisms, observed outcomes matched on to implementation strategy and intervention. These will be then refined into sub-codes, for example to capture specificities of data collected from different interviewee roles or to highlight different dimensions featuring in a main code. For example, for intervention strategy, the sub-codes of "fidelity", "dose", "adaptations", "reach" may be developed. Mechanisms may be clustered around the sub-codes "ideas" and "resources".

### *Patient and Public Involvement*

A person living with dementia and a family carer of somebody living with dementia are members of the advisory board of the co-creation initiative. In this capacity they are overseeing the development of the study, provided feedback in the design phase of the intervention and are expected to review the results.



For peer review only - http://bmjopen.bmj.com/site/about/guidelines.xhtml

## ETHICS AND DISSEMINATION

Ethical and research governance approvals have been obtained from the West Midlands - South Birmingham Research Ethics Committee [REC reference 16/WM/0397].

The study has been designed as part of a 5-year co-creation initiative by the local working group in collaboration with the research team. Regular updates about the implementation of the intervention are regularly shared with the working group and with the wider group of stakeholders involved in the co-creation initiative.

Findings of the work will be disseminated across professionals e.g. at national conferences and events for providers and commissioners of care and support for people with dementia, policy makers in the area of dementia, representatives of services users. Articles stemming from this work will be submitted for publication to peer-reviewed journals and to national and international scientific conferences in the field of applied health research.

For peer review only

## REFERENCES

1 Dalmer NK. 'Add info and stir': an institutional ethnographic scoping review of family care-givers' information work. *Ageing Soc* 2018;:1–27. doi:10.1017/S0144686X18001125

2 Harris R, Wathen N, Wyatt S. Working to be healthy: empowering consumers or citizens? In: Harris R, Wathen N, Wyatt S, eds. *Configuring Health Consumers: Health Work and the Imperative of Personal Responsibility*. New York, NY: : Palgrave Macmillan 2010. 211–24.

3 Washington KT, Meadows SE, Elliott SG, *et al.* Information needs of informal caregivers of older adults with chronic health conditions. *Patient Educ Couns* 2011;**83**:37–44. doi:10.1016/j.pec.2010.04.017

4 Mastel-Smith B, Stanley-Hermanns M. 'It's like we're grasping at anything': Caregivers' education needs and preferred learning methods. *Qual Health Res* 2012;**22**:1007–15. doi:10.1177/1049732312443739

5 Dunbrack J. The information needs of informal caregiversinvolved in providing support to a critically ill loved one. 2005.

6 Vaingankar JA, Subramaniam M, Picco L, *et al.* Perceived unmet needs of informal caregivers of people with dementia in Singapore. *Int Psychogeriatrics* 2013;**25**:1605–19. doi:10.1017/S1041610213001051

7 Prorok J, Horgan S, Seitz D. Health care experiences of people with dementia and their caregivers: a meta-ethnographic analysis of qualitative studies. *CMAJ* 2013;**185**:E669-80.

8 Greenwood N, Habibi R, Smith R, *et al.* Barriers to access and minority ethnic carers' satisfaction with social care services in the community: A systematic review of qualitative and quantitative literature. *Heal Soc Care Community* 2015;**23**:64–78. doi:10.1111/hsc.12116

9 Afram B, Verbeek H, Bleijlevens MHC, *et al.* Needs of informal caregivers during transition from home towards institutional care in dementia: A systematic review of qualitative studies. Int. Psychogeriatrics. 2015;**27**:891–902. doi:10.1017/S1041610214002154



For peer review only - http://bmjopen.bmj.com/site/about/guidelines.xhtml

10 Sutcliffe CL, Roe B, Jasper R, *et al.* People with dementia and carers' experiences of dementia care and services: Outcomes of a focus group study. *Dementia* 2015;**14**:769–87. doi:10.1177/1471301213511957

11 Downs M, Clibbens R, Rae C, *et al.* What Do General Practitioners Tell People with Dementia and their Families about the Condition? *Dementia* 2002;**1**:47–58. doi:10.1177/147130120200100106

12 Van Hout HP, Vernooij-Dassen MJ, Jansen DA, *et al.* Do general practitioners disclose correct information to their patients suspected of dementia and their caregivers? A prospective observational study. *Aging Ment Heal* 2006;**10**:151–5. doi:10.1080/13607860500310468

13 Allen F, Cain R, Meyer C. Seeking relational information sources in the digital age: A study into information source preferences amongst family and friends of those with dementia. Dementia. 2018. doi:10.1177/1471301218786568

14 Wackerbarth SB, Johnson MMS. Essential information and support needs of family caregivers. *Patient Educ Couns* 2002;**47**:95–100. doi:10.1016/S0738-3991(01)00194-X

15 Greenwood N, Dphil RH, Smith Msc R, *et al.* Barriers to access and minority ethnic carers' satisfaction with social care services in the community: a systematic review of qualitative and quantitative literature. Published Online First: 2014. doi:10.1111/hsc.12116

16 Koehn S. Negotiating candidacy: ethnic minority seniors' access to care. *Ageing Soc* 2009;**29**:585–608. doi:10.1017/S0144686X08007952

17 Kelly F, Innes A. Facilitating independence: The benefits of a post-diagnostic support project for people with dementia. *Dementia* 2016;**15**:162–80. doi:10.1177/1471301214520780

18 Pinnock H, Barwick M, Carpenter CR, *et al.* Standards for Reporting Implementation Studies (StaRI) Statement. *BMJ* 2017;**356**:i6795. doi:10.1136/bmj.i6795

19 Pinnock H, Barwick M, Carpenter CR, *et al.* Standards for Reporting Implementation Studies (StaRI): explanation and elaboration document. *BMJ Open* 2017;**7**:e013318. doi:10.1136/bmjopen-2016-013318

20 Susman G, Evered R. An assessment of the scientific merits of action research. *Adm Sci Q* 1978;**23**:582–603.

21 Sustersic M, Gauchet A, Foote A, *et al.* How best to use and evaluate Patient Information Leaflets given during a consultation: a systematic review of literature reviews. *Heal Expect* 2017;**20**:531–42. doi:10.1111/hex.12487

22 Pinnock H, Barwick M, Carpenter CR, *et al.* Standards for Reporting Implementation Studies (StaRI) Statement. *BMJ* 2017;**356**. doi:10.1136/bmj.i6795

23 NHS Digital. QOF 2018/19. https://qof.digital.nhs.uk/ (accessed 6 Mar 2020).

24 Proctor EK, Powell BJ, McMillen JC. Implementation strategies: recommendations for specifying and reporting. *Implement Sci* 2013;**8**:139. doi:10.1186/1748-5908-8-139

25 Mazza D, Bairstow P, Buchan H, *et al.* Refining a taxonomy for guideline implementation: results of an exercise in abstract classification. *Implement Sci* 2013;**8**:32. doi:10.1186/1748-5908-8-32



For peer review only - http://bmjopen.bmj.com/site/about/guidelines.xhtml

26 Powell BJ, McMillen JC, Proctor EK, *et al.* A compilation of strategies for implementing clinical innovations in health and mental health. Med. Care Res. Rev. 2012;**69**:123–57. doi:10.1177/1077558711430690

27 Proctor E, Silmere H, Raghavan R, *et al.* Outcomes for implementation research: conceptual distinctions, measurement challenges, and research agenda. *Adm Policy Ment Health* 2011;**38**:65–76. doi:10.1007/s10488-010-0319-7

28 Moore GF, Audrey S, Barker M, *et al.* Process evaluation of complex interventions: Medical Research Council guidance. *BMJ* 2015;**350**:h1258. doi:10.1136/bmj.h1258

29 Hoffmann TC, Glasziou PP, Boutron I, *et al.* Better reporting of interventions: Template for intervention description and replication (TIDieR) checklist and guide. *BMJ* 2014;**348**. doi:10.1136/bmj.g1687

30 Curran GM, Bauer M, Mittman B, *et al.* Effectiveness-implementation hybrid designs: combining elements of clinical effectiveness and implementation research to enhance public health impact. *Med Care* 2012;**50**:217–26. doi:10.1097/MLR.0b013e3182408812

31 Proctor E, Silmere H, Raghavan R, *et al.* Outcomes for implementation research: Conceptual distinctions, measurement challenges, and research agenda. *Adm Policy Ment Heal Ment Heal Serv Res* 2011;**38**:65–76. doi:10.1007/s10488-010-0319-7

32 Pawson R, Tilley N. *Realistic Evaluation*. Thousand Oaks, CA: : SAGE publications 1997.

33 Astbury B, Leeuw FL. Unpacking Black Boxes: Mechanisms and Theory Building in Evaluation. *Am J Eval* 2010;**31**:363–81. doi:10.1177/1098214010371972

34 Greenhalgh T, Humphrey C, Hughes J, *et al.* How do you modernize a health service? A realist evaluation of whole-scale transformation in london. *Milbank Q* 2009;**87**:391–416. doi:10.1111/j.1468-0009.2009.00562.x

35 Johannessen A, Engedal K, Haugen PK, *et al.* "To be, or not to be": experiencing deterioration among people with young-onset dementia living alone. *Int J Qual Stud Health Well-being* 2018;**13**:1490620. doi:10.1080/17482631.2018.1490620

36 Samsi K, Manthorpe J. Everyday decision-making in dementia: findings from a longitudinal interview study of people with dementia and family carers. *Int Psychogeriatrics* 2013;**25**:949–61. doi:10.1017/S1041610213000306

37 Windle G, Joling KJ, Howson-Griffiths T, *et al.* The impact of a visual arts program on quality of life, communication, and well-being of people living with dementia: a mixed-methods longitudinal investigation. *Int Psychogeriatrics* 2018;**30**:409–23. doi:10.1017/S1041610217002162

38 Keller HH, Martin LS, Dupuis S, *et al.* Strategies to support engagement and continuity of activity during mealtimes for families living with dementia; a qualitative study. *BMC Geriatr* 2015;**15**:119. doi:10.1186/s12877-015-0120-2

39 Barnes M, Henwood F, Smith N. Information and care: A relational approach. *Dementia* 2016;**15**:510–25. doi:10.1177/1471301214527750

40 Moye J, Marson DC. Assessment of decision-making capacity in older adults: an emerging area of practice and research. *J Gerontol B Psychol Sci Soc Sci* 2007;**62**:P3–11.

41 Karel MJ, Gurrera RJ, Hicken B, *et al.* Reasoning in the capacity to make medical




For peer review only - http://bmjopen.bmj.com/site/about/guidelines.xhtml

decisions: the consideration of values. *J Clin Ethics* 2010;**21**:58–71.

42 Karlawish J. Measuring Decision-Making Capacity in Cognitively Impaired Individuals. *Neurosignals* 2008;**16**:91–8. doi:10.1159/000109763

43 QSR International Pty Ltd. NVivo qualitative data analysis software. 2018.

## AUTHORS' CONTRIBUTIONS

CDP was involved in the design of the intervention and of the implementation strategy, conceived the evaluation framework, drafted the first version of the protocol and revised it following discussions with the other Authors. JO and MA oversaw the design of the intervention and of the implementation strategy, contributed to developing the evaluation framework and revised the manuscript. CB was involved in the design of the intervention and of the implementation strategy, supported local engagement and revised the manuscript. RG was involved in the design of the intervention and of the implementation strategy, supported local engagement and revised the manuscript. All authors have read and approved the final manuscript.

## FUNDING STATEMENT

CDP, JO, RG and MA were supported by The Health Foundation, grant number 1274233. CB received no specific grant from any funding agency in the public, commercial or not-for-profit sectors.

## COMPETING INTERESTS

None to declare

For peer review only

Figure 1 - The phases of the co-creation initiative

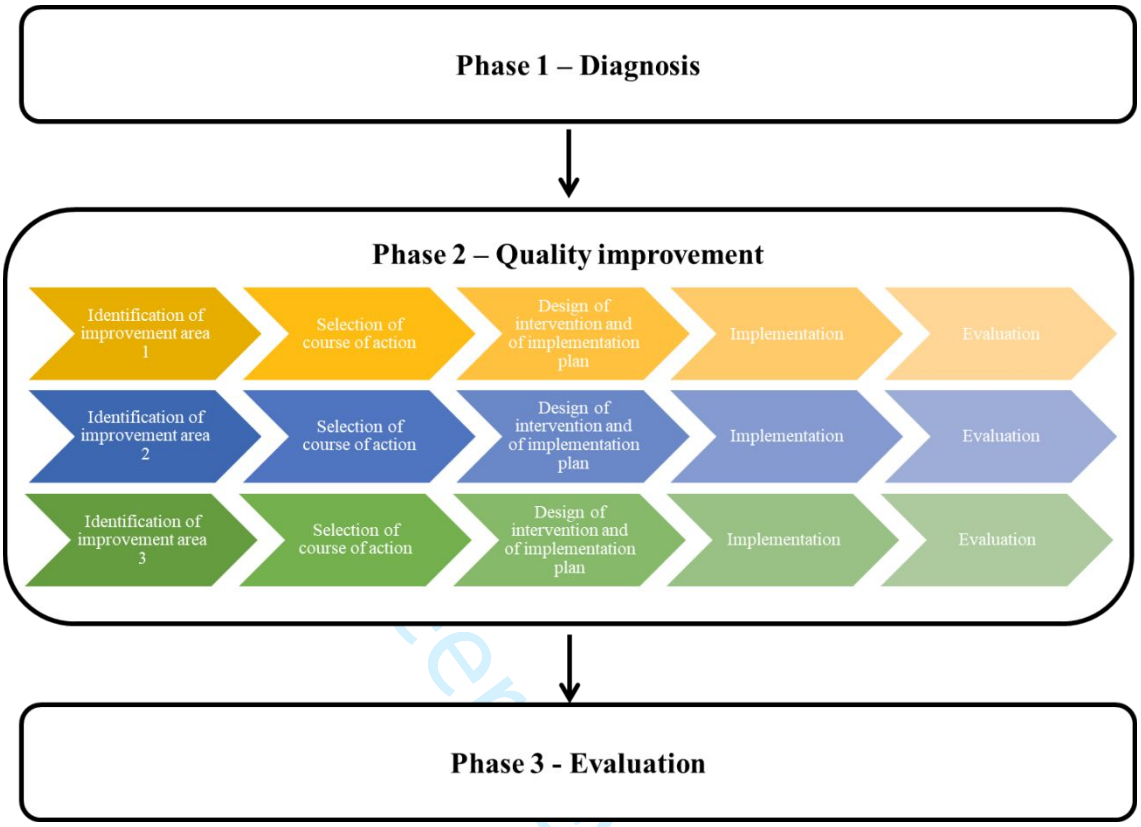

**Figure 2 – Timeline of the development of the intervention**

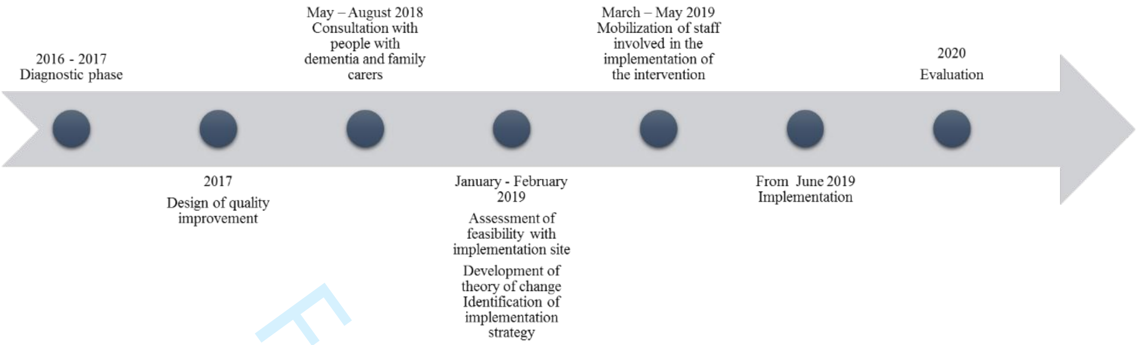


For peer review only

**Figure 3 – The evaluation framework of the intervention**

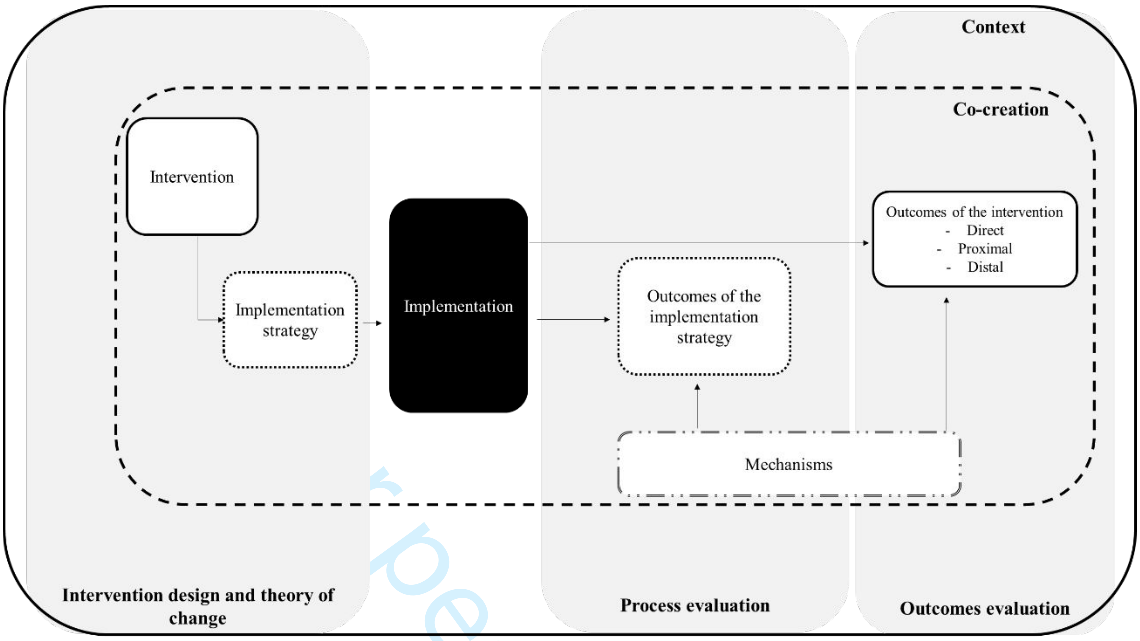


For peer review only

# Figure 4 – Data collection timeline

| | | Month 1 | Month 2 | Month 3 | Month 4 | Month 5 | Month 6 | Month 7 | Month 8 | Month 9 | Month 10 |
|---|---|---|---|---|---|---|---|---|---|---|---|
| | **Implementation** | **Start** | Ongoing implementation | | | | | | | | |
| **People with dementia and family carers** | **Recruitment** | Ongoing recruitment | | | | | | | | | |
| | **Data collection** | | Interview 1 | | | | | | Interview 2 | | |
| **CPNs, team and service managers** | **Data collection** | | | | | | | Interviews | | | |
| **Local service providers** | **Data collection** | Activity log | | | | | | | Interviews | | |
| **Leaflet working group** | **Data collection** | | | | | | | | | | Focus group |

For peer review only

**Table 1 – StaRI checklist (implementation strategy)** [1]

| IMPLEMENTATION STRATEGY (i.e. how the intervention was implemented) | | |
|---|---|---|
| **Item** | **Description and reference to methodological frameworks and reporting standards** | |
| 1. Title | | Effectiveness-implementation hybrid type 2 study evaluating an intervention to support 'information work' in dementia care |
| 2. Abstract | | See Abstract of the protocol |
| 3. Introduction | To identify the problem | See Introduction of the protocol |
| 4. Introduction | Rationale for the implementation strategy and the intervention | See Intervention development of the protocol |
| 5. Aims and objectives | | 1. To assess the unfolding of the implementation strategy and its outcomes<br>2. To assess the outcomes of the intervention as perceived by people living with dementia and their family carers, staff implementing the intervention and local organizations and to explain how and why the intervention contributed to achieve these outcomes<br>3. To reflect on the extent to which, how and why the co-creation initiative has influenced the design, implementation and impact of the intervention |
| 6. The design and key features of the evaluation | | The evaluation of the implementation strategy aims at understanding the feasibility of the intervention by investigating how the actual implementation process has unfolded in practice and identifying any discrepancies between the planned and the actual implementation process |
| 7. Context | Context in which the intervention is implemented | Community mental health services for older people (CMHSOP) within a mental health foundation trust in the North East of England (UK)<br>- North East of England is an under-researched area compared to other parts of England<br>- Local health economy with average recorded diagnosed dementia prevalence (for 2018-2019, local recorded prevalence rate 0.9% vs national recorded prevalence rate 0.8%) and average recorded annual review rate (for 2018-2019, local annual review rate 79.2% vs national recorded prevalence rate 77.9%)[2]<br>- Mental health foundation trust, established in 2006 providing a range of mental health, learning disability and eating disorder services for around two million people across a large and diverse geographical area<br>- CMHSOP provide community based services for older people experiencing cognitive impairment and mental health problems. Organized around four localities, each locality team includes community psychiatric nurses (CPNs), occupational therapists, physiotherapists, psychologists, support workers to provide multi-disciplinary support |



For peer review only - http://bmjopen.bmj.com/site/about/guidelines.xhtml

<table>
<tr><th colspan="3">IMPLEMENTATION STRATEGY (i.e. how the intervention was implemented)</th></tr>
<tr><th>Item</th><th>Description and reference to methodological frameworks and reporting standards</th><th></th></tr>
<tr><td></td><td></td><td>Intervention was developed in the context of a 5 year action research co-creation initiative funded by an English charity, involving researchers from three English universities and a research facilitator from the local commissioning support unit alongside a constellation of local stakeholders</td></tr>
<tr><td>8. Target sites</td><td></td><td>Four locality teams, staffing levels and caseload are reported in the table below<br><br>
<table>
<tr><th>Team</th><th>Total staff</th><th>CPNs</th><th>Caseload</th></tr>
<tr><td>Team 1</td><td>48</td><td>9</td><td>1,368</td></tr>
<tr><td>Team 2</td><td>43</td><td>8</td><td>1,377</td></tr>
<tr><td>Team 3</td><td>27</td><td>5</td><td>787</td></tr>
<tr><td>Team 4</td><td>26</td><td>5</td><td>791</td></tr>
</table></td></tr>
<tr><td>9. Implementation strategy</td><td>Description of the implementation strategy in accordance with Proctor et al.'s framework for reporting implementation strategies[3]</td><td>Name and definition of the implementation strategy: bundle of professional and material interventions aiming at the development, adoption and use of the dementia leaflet among CPNs (based on framework by [4,5]<br><br>Specification of the implementation strategy<br>- Actors: Community Psychiatric Nurses (CPNs) and team managers and service manager across four CMHSOP teams<br>- Actions: Described in accordance with relevant phase of the Plan-Do-Study-Act (PDSA) framework<br>  - Plan:<br>    Professional interventions<br>    ○ Established a local working group in charge of overseeing the development the leaflet and its implementation strategy<br>    ○ Iteratively developed a dementia leaflet, embedding feedback from people with dementia, family carers, professionals from local organisations providing care and support to people with dementia and family carers<br>    ○ Identified the implementation strategy via CPNs based in CMHSOP, identified local champion (service manager), identified implementation sites</td></tr>
</table>



For peer review only

| IMPLEMENTATION STRATEGY (i.e. how the intervention was implemented) | | |
|---|---|---|
| **Item** | **Description and reference to methodological frameworks and reporting standards** | |
| | | o Organised mobilisation activities with implementation sites, including information sessions at team meetings and email reminders<br>Material intervention<br>o Printed copies of the leaflet made available to implementers<br>- Do: CPNs offer a copy of the leaflet to people with dementia or their family carers attending an appointment (any type)<br>- *Action targets*: People living with dementia (any type of dementia, diagnosed at any time) and their family carers attending an appointment with their CPNs. CPNs were expected to use their clinical judgment in identifying patients or family members deemed to be ready to receive the leaflet<br>- *Temporality*: The leaflet can be offered at any point during the period in which the patient was under the care of the CMHSOP (at initial assessment, at post diagnosis, at reviews, at discharge)<br>- *Dose:* Depending on the individual readiness to receive the leaflet and individual and family circumstance, CPNs decide whether to offer or not the leaflet as part of their appointment with the patient. The leaflet could be offered more than once at different points in time |
| 10. Sub-groups | Any subgroups recruited for additional research tasks, and/or nested studies | Not applicable to this study |
| **Evaluation** | | |
| 11. Outcomes | Using Proctor et al.'s taxonomy of outcomes for implementation research [6] | - Acceptability of the intervention by CPNs: perception among CPNs that the intervention is agreeable, palatable, or satisfactory<br>- Adoption of the intervention by CPNs: CPNs' intention, initial decision, or action to try the intervention<br>- Appropriateness of the intervention implemented as judged by CPNs: perceived fit, relevance, or compatibility of the intervention for the setting of CMHSOP and perceived fit of the intervention to address the issue<br>- Cost of the intervention for the implementers<br>- Feasibility of the implementation of the intervention: the extent to which the intervention can be successfully used or carried out within the setting of the CMHSOP<br>- Penetration of the intervention: integration of the intervention within the CMHSOP<br>- Sustainability of the intervention: extent to which the intervention is maintained or institutionalised within the CMHSOP |



For peer review only - http://bmjopen.bmj.com/site/about/guidelines.xhtml

| IMPLEMENTATION STRATEGY (i.e. how the intervention was implemented) | | |
|---|---|---|
| **Item** | **Description and reference to methodological frameworks and reporting standards** | |
| 12. Process evaluation objectives and outcomes related to the mechanism(s) through which the strategy is expected to work | Objectives and outcomes related to the mechanism by which the strategy is expected to work, using MRC framework for complex interventions [7] | - Fidelity of the intervention: adherence to the intervention protocol<br>- Adaptations of the intervention<br>- Dose: the quantity of intervention implemented<br>- Reach: whether the intended audience comes into contact with the intervention, and how |
| 13. Data collection methods | | In-depth interviews with CPNs, team managers and service manager of CMHSOP |
| 14. Sample size | | - Up to 12 CPNs from four locality teams<br>- Four team managers<br>- One service manager |
| 15. Methods of analysis | | Thematic content analysis of interview data |



For peer review only

**Table 2 –StaRI checklist (intervention)** [1]

| INTERVENTION (i.e. the actual intervention being implemented) | | |
|---|---|---|
| **Item** | **Description and reference to methodological frameworks and reporting standards** | |
| 1. Title | | Effectiveness-implementation hybrid type 2 study evaluating an intervention to support 'information work' in dementia care |
| 2. Abstract | | See Abstract of the protocol |
| 3. Introduction | To identify the problem | See Introduction of the protocol |
| 4. Introduction | Rationale for the implementation strategy and the intervention | See Intervention development of the protocol |
| 5. Aims and objectives | | 1. To assess the unfolding of the implementation strategy and its outcomes<br>2. To assess the outcomes of the intervention as perceived by people living with dementia and their family carers, staff implementing the intervention and local organizations and to explain how and why the intervention contributed to achieve these outcomes<br>3. To reflect on the extent to which, how and why the co-creation initiative has influenced the design, implementation and impact of the intervention |
| 6. The design and key features of the evaluation | | The evaluation of the intervention aims at assessing the outcomes of the intervention with respect to people with dementia and family carers (primary outcomes), to the implementers of the intervention (proximal outcomes) and to local service providers (distal outcomes) |
| 7. Context | Context in which the intervention was implemented | As per implementation strategy described above |
| 8. Target population | Characteristics of the population targeted by the intervention and eligibility criteria | People living with dementia (any type of dementia, diagnosed at any time) and their family carers attending an appointment with their CPNs. CPNs were expected to use their clinical judgment in identifying patients or family members deemed to be ready to receive the leaflet |
| 9. Intervention | Description of the intervention, provided in accordance with the TIDieR checklist [8] | Name: Leaflet about local care and support available to people living with dementia and family carers<br><br>Why: in the diagnostic phase of the co-creation initiative, people living with dementia and family carers consistently reported a lack of timely, up-to-date information provided in a user-friendly format which hinders their 'information work'. The intervention has been designed to provide a short, up-to-date, paper-based source of information that could |



For peer review only - http://bmjopen.bmj.com/site/about/guidelines.xhtml

| INTERVENTION (i.e. the actual intervention being implemented) | | |
|---|---|---|
| **Item** | **Description and reference to methodological frameworks and reporting standards** | |
| | | be handed out and used by CPNs in their encounter with people with dementia and their family carers and support their ‘information work’<br><br>What (materials): An A4 three-folded, two-sided leaflet printed in high contrast colour combination. Information are organized in four themes, for which relevant services, a short description and contact details are provided<br><br>What (procedures): CPNs offer the leaflet to patients and their family carers as part of their clinical encounters<br><br>Who: 27 qualified mental health nurses (CPNs) part of the organic cells working across four locality team of the local CMHSOP who assess, diagnose, review medications and offer post-diagnostic support to people with dementia and signpost family carers to services available to them<br><br>How: the leaflet is offered in the context of the appointment attended by the person with dementia and their family carer. The CPN can offer the leaflet in response to a current specific need of the person with dementia or of the family carer (either explicitly reported or latent) or opportunistically, to make them aware of local services which could be of interest either at the present time or in the future<br><br>Where: the intervention takes place in the outpatient clinics of the CMHSOP services of the local mental health foundation trust<br><br>When and how much: CPNs can offer the leaflet at any point in the period when the patient is under the care of the CMHSOP (e.g. at initial assessment, at diagnosis, in the post diagnostic appointment, at review, upon transfer of care back to GP). It could be offered by the CPNs more than once at different points in time<br><br>Tailoring: CPNs are expected to use their clinical judgement in deciding whether, when, how and to whom to offer the leaflet based on<br>- patients or families’ readiness to accept the leaflet (e.g. some patients do not temporarily - or will never - accept a diagnosis of dementia)<br>- length of the appointments (e.g. initial assessment can take up to 90 minutes and patients and families may be overloaded with information) |



For peer review only - http://bmjopen.bmj.com/site/about/guidelines.xhtml

| INTERVENTION (i.e. the actual intervention being implemented) | | |
|---|---|---|
| **Item** | **Description and reference to methodological frameworks and reporting standards** | |
| | | - type of appointments (e.g. titration appointments may cause disappointment in patients) |
| 10. Sub-groups | Any subgroups recruited for additional research tasks, and/or nested studies | Not applicable to this study |
| **Evaluation** | | |
| 11. Outcomes | Primary and other outcomes of the intervention | For people with dementia and family carers (primary outcomes)<br>- their satisfaction with the availability of information on local services (e.g. in terms of timing and quality of information)<br>- their awareness of local services<br>- in the longer term, their access and use of local services<br><br>For implementers of the intervention (proximal outcomes)<br>- CPN, team and service managers' awareness of local services and support available to people diagnosed with dementia and to their family carers<br>- CPN, team and service managers' confidence to hold conversations around using services available to those living with dementia and their family carers<br><br>For local service providers (distal outcomes)<br>- volume of referrals into their services from people with dementia and their family carers<br>- appropriateness of the use of services among those living with dementia and family carers |
| 12. Process evaluation objectives and outcomes related to the mechanism(s) through which the strategy is expected to work | Objectives and outcomes related to the mechanism by which the strategy is expected to work, using MRC framework for complex interventions [7] | As per implementation strategy |
| 13. Data collection methods | | People with dementia and family carers<br>- Longitudinal semi-structured in-depth interviews: interview 1 carried out 4-6 weeks after the appointment with the CPN when they receive the leaflet; interview 2 (follow-up) scheduled about 5 months after interview 1<br><br>Implementers of the intervention |



For peer review only - http://bmjopen.bmj.com/site/about/guidelines.xhtml

| INTERVENTION (i.e. the actual intervention being implemented) | | |
|---|---|---|
| **Item** | **Description and reference to methodological frameworks and reporting standards** | |
| | | - Semi-structured in-depth interviews with CPNs, team managers and service manager of CMHSOP<br><br>Local service providers<br>- Activity log recording activity data for 24 months (12 months before/after the implementation of the intervention)<br>- Semi-structured in-depth interviews with managers<br><br>Local working group<br>- focus group with about 12 participants (including commissioners, statutory service providers, staff from CMHSOP, managers from third sector organizations, a person with dementia and a family carer) |
| 14. Sample size | | People living with dementia and family carers<br>- Up to 30 people living with dementia (any type of dementia, diagnosed at any time, willing and able to consent) and family carers (of somebody with any type of dementia, any degree of severity, diagnosed at any time)<br><br>Implementers of the intervention<br>- Up to 12 CPNs from 4 locality teams<br>- Four team managers<br>- Service manager<br><br>Local service providers<br>- managers from up to five local organisations providing care and support to people living with dementia and their family carers<br><br>Local working group<br>- about 12 participants attending the focus group (including commissioners, statutory service providers, staff from CMHSOP, managers from third sector organizations, a person with dementia and a family carer) |
| 15. Methods of analysis | | Thematic content analysis of interview and focus group data<br>Descriptive statistics for activity data for local service providers for triangulation purposes |



For peer review only - http://bmjopen.bmj.com/site/about/guidelines.xhtml

## REFERECES

1 Pinnock H, Barwick M, Carpenter CR, *et al.* Standards for Reporting Implementation Studies (StaRI) Statement. *BMJ* 2017;**356**. doi:10.1136/bmj.i6795

2 NHS Digital. QOF 2018/19. https://qof.digital.nhs.uk/ (accessed 6 Mar 2020).

3 Proctor EK, Powell BJ, McMillen JC. Implementation strategies: recommendations for specifying and reporting. *Implement Sci* 2013;**8**:139. doi:10.1186/1748-5908-8-139

4 Mazza D, Bairstow P, Buchan H, *et al.* Refining a taxonomy for guideline implementation: results of an exercise in abstract classification. *Implement Sci* 2013;**8**:32. doi:10.1186/1748-5908-8-32

5 Powell BJ, McMillen JC, Proctor EK, *et al.* A compilation of strategies for implementing clinical innovations in health and mental health. Med. Care Res. Rev. 2012;**69**:123–57. doi:10.1177/1077558711430690

6 Proctor E, Silmere H, Raghavan R, *et al.* Outcomes for implementation research: conceptual distinctions, measurement challenges, and research agenda. *Adm Policy Ment Health* 2011;**38**:65–76. doi:10.1007/s10488-010-0319-7

7 Moore GF, Audrey S, Barker M, *et al.* Process evaluation of complex interventions: Medical Research Council guidance. *BMJ* 2015;**350**:h1258. doi:10.1136/bmj.h1258

8 Hoffmann TC, Glasziou PP, Boutron I, *et al.* Better reporting of interventions: Template for intervention description and replication (TIDieR) checklist and guide. *BMJ* 2014;**348**. doi:10.1136/bmj.g1687

For peer review only



For peer review only - http://bmjopen.bmj.com/site/about/guidelines.xhtml

# Effectiveness-implementation hybrid type 2 study evaluating an intervention to support 'information work' in dementia care: implementation study protocol

| | |
|---|---|
| Journal: | *BMJ Open* |
| Manuscript ID | bmjopen-2020-038397.R1 |
| Article Type: | Protocol |
| Date Submitted by the Author: | 06-Jul-2020 |
| Complete List of Authors: | De Poli, Chiara; London School of Economics and Political Science, Department of Social Policy and Department of Management<br>Oyebode, Jan; University of Bradford, School of Dementia Studies<br>Binns, Christopher; Tees, Esk and Wear Valleys NHS Trust<br>Glover, Richard; NHS North of England Commissioning Support Unit<br>Airoldi, Mara; University of Oxford, Blavatnik School of Government |
| <b>Primary Subject Heading</b>: | Geriatric medicine |
| Secondary Subject Heading: | Patient-centred medicine, Health services research |
| Keywords: | Dementia < NEUROLOGY, Quality in health care < HEALTH SERVICES ADMINISTRATION & MANAGEMENT, QUALITATIVE RESEARCH |
| | |

SCHOLARONE™ Manuscripts

*I, the Submitting Author has the right to grant and does grant on behalf of all authors of the Work (as defined in the below author licence), an exclusive licence and/or a non-exclusive licence for contributions from authors who are: i) UK Crown employees; ii) where BMJ has agreed a CC-BY licence shall apply, and/or iii) in accordance with the terms applicable for US Federal Government officers or employees acting as part of their official duties; on a worldwide, perpetual, irrevocable, royalty-free basis to BMJ Publishing Group Ltd ("BMJ") its licensees and where the relevant Journal is co-owned by BMJ to the co-owners of the Journal, to publish the Work in this journal and any other BMJ products and to exploit all rights, as set out in our licence.*

*The Submitting Author accepts and understands that any supply made under these terms is made by BMJ to the Submitting Author unless you are acting as an employee on behalf of your employer or a postgraduate student of an affiliated institution which is paying any applicable article publishing charge ("APC") for Open Access articles. Where the Submitting Author wishes to make the Work available on an Open Access basis (and intends to pay the relevant APC), the terms of reuse of such Open Access shall be governed by a Creative Commons licence – details of these licences and which Creative Commons licence will apply to this Work are set out in our licence referred to above.*

*Other than as permitted in any relevant BMJ Author's Self Archiving Policies, I confirm this Work has not been accepted for publication elsewhere, is not being considered for publication elsewhere and does not duplicate material already published. I confirm all authors consent to publication of this Work and authorise the granting of this licence.*

For peer review only

# Effectiveness-implementation hybrid type 2 study evaluating an intervention to support 'information work' in dementia care: implementation study protocol

Chiara De Poli [a]
Jan Oyebode [b]
Chris Binns [c]
Richard Glover [d]
Mara Airoldi [e]

[a] Corresponding author. Department of Social Policy and Department of Management, London School of Economics and Political Science, Houghton Street, WC2A 2A London, UK, c.de-poli@lse.ac.uk, phone +44 (0)2079556749

[b] Centre for Applied Dementia Studies, Faculty of Health Studies, University of Bradford, Bradford, UK

[c] Tees, Esk and Wear Valleys NHS Trust, Durham, UK

[d] NHS North of England Commissioning Support, Durham, UK

[e] Blavatnik School of Government, University of Oxford, Oxford, UK

For peer review only

## ABSTRACT

### Introduction

Patients with long-term conditions consistently report a lack of information around services and support available to them. This unmet need for information is significant among people with dementia and family carers. A quality improvement intervention is being carried out to tackle this issue as part of a co-creation initiative in the North East of England (UK). The intervention consists of the dissemination (via the local community mental health services for older people) of a leaflet about services available to people with dementia and their family carers in the study site. This protocol is reported in accordance with the Standards for Reporting Implementation Studies (StaRI).

### Methods and analysis

This effectiveness-implementation hybrid type 2 study aims at understanding (1) the unfolding and outcomes of the implementation strategy, (2) the outcomes of the intervention (for people with dementia and family carers, staff implementing the intervention, local service providers), (3) the contribution of co-creation to the design and implementation of the intervention and its outcomes. The prospective theory of change of the intervention articulated by local stakeholders is used as a reference framework against which to assess the implementation and outcomes of the intervention. Evaluation data will be collected through in-depth interviews with people with dementia and family carers receiving the intervention, staff implementing the intervention, managers from local service providers. Referral data from local service providers will be collected to triangulate the interview data. A focus group with key stakeholders will support the sense-making of findings. The realist configuration of mechanism – context – outcome, operationalized using an information behaviour model, will inform data analysis and interpretation.

### Ethics and dissemination

Ethical and research governance approvals have been obtained from the West Midlands - South Birmingham Research Ethics Committee. The results of the study will be submitted for publication in peer-reviewed journals and disseminated through conferences.


For peer review only

**Strengths and limitations of this study**

- The study is reported in accordance with the Standards for Reporting Implementation Studies (StaRI) framework complemented by additional relevant reporting standards, such as the Template for intervention description and replication (TIDieR) checklist and Proctor et al.'s framework for specifying and reporting implementation strategies
- The evaluation framework integrates the evaluation of the implementation strategy and of the implemented intervention both in terms of process and of outcomes
- The mixed-method evaluation uses data collected through in-depth interviews with three sets of stakeholders, services' referral data and a focus group
- In-depth, longitudinal interviews with people with dementia are planned to explore their experiences of receiving and using the leaflet, however recruitment can be difficult and dropout rate can be high
- The evaluation will provide insights regarding the process and impact of delivering and receiving the intervention but will have no comparison condition

For peer review only



For peer review only - http://bmjopen.bmj.com/site/about/guidelines.xhtml

## INTRODUCTION

Patient activation, self-management, ageing in place, enablement and shared-decision making are recurrent themes in policy discourses across health systems addressing ageing populations, chronic diseases, multi-morbidity and complex needs.

Underpinning, albeit often implicit, assumptions of such policy discourses is the idea that by providing information, patients and family carers are supported to make better choices, have more agency on managing their own health (or the health of the person they care for), may feel empowered and ultimately may report better outcomes and satisfaction with the care and support they receive.

However, such a view fails to acknowledge the ‘information work’, i.e. the process of seeking, using, evaluating and sharing of information[1] that is required from patients and family carers in order for them to be able to make better choices or to play a more active role in their own or others’ care.[2] Also, it assumes patients and family carers are physically and cognitively able to carry out information work, and it overlooks the emotional processes that influence adaptation to long-term conditions, which may influence whether information work is successfully undertaken.

Extant research both documents and describes the unmet information need of patients and family carers and also offers possible explanations for it.[3–5] This issue has been recognised as highly significant among people with dementia and family carers,[6–10] whose information needs span beyond the topics usually addressed by information materials available, leaving them with some information gaps.[11,12] Moreover, people living with dementia identify a preference to receive information directly from health and social care professionals rather than over the internet, which is the primary source of information to which they are often signposted.[13] They highlight that the timeliness of their information needs, which vary along the dementia trajectory,[14] is seldom taken into account by information providers and resources. They also expect up-to-date information to be provided in a culturally acceptable way and in easy to understand language.[8,15,16] Several user-centred models in the fields of information science have been developed to unpack users’ information behaviour and explore how the actual process of information need, seeking and use occurs, in order to explain the observed variations in information need.[17] Notwithstanding the specificities of each, these models consistently acknowledge that information is context-sensitive and uniquely dependent on the individual user, who actively constructs its subjective meaning.[18]

Building on this research, an implementation study is being carried out as part of a co-creation initiative in dementia care taking place in the North East of England (UK). The study aims at designing, implementing and evaluating a leaflet about local services providing care and support to people with dementia and their family carers living in the study site.

This article reports the protocol for the evaluation of the implementation study in accordance with the Standards for Reporting Implementation Studies (StaRI) framework and adopts the recommended dual strands for reporting, one about the intervention being implemented and the other about its implementation strategy (i.e. the ‘bundle’ of techniques used to enhance the adoption, integration into routine practice and sustainability of the intervention) (Table 1 and Table 2).[19,20]

The evaluation has three aims. First, it intends to review the unfolding and outcomes of the implementation strategy. Secondly, it aims to assess the outcomes of the intervention as perceived by people living with dementia and their family carers, staff implementing the intervention and local organizations providing care and support to people with dementia and



For peer review only - http://bmjopen.bmj.com/site/about/guidelines.xhtml

family carers and to explain how and why the intervention contributed to achieve these outcomes. Thirdly, it will reflect on the extent to which, how and why the co-creation initiative has influenced the design, implementation and impact of the intervention.

## INTERVENTION DEVELOPMENT

The implementation study herein described has been developed as part of a co-creation initiative in dementia care taking place in the North East of England (UK). The initiative, started in 2015 and with expected completion at the end of 2020, involves a constellation of local stakeholders, including people living with dementia and their family carers, health and care professionals, service managers and commissioners alongside a research team, encompassing university-based researchers and a local research facilitator.

Grounded in the principles of action research, co-creation activities were organised in three broad phases (Figure 1).[21] The diagnostic phase (phase 1, 2015-2018) mapped the local dementia care system and identified its strengths and weaknesses. The improvement phase (phase 2, 2017-2018) articulated and designed three interventions to address the priority issues identified in the diagnostic phase. Each improvement area was addressed via a sub-cycle of action research. Local stakeholders with an investment in the respective improvement areas were invited to work with the research team to identify different available options to address the improvement areas, select a course of action and accordingly design an intervention and define its implementation.

The planned evaluation (phase 3, 2020) will focus on both the improvement interventions and the co-creation initiative. With respect to the first, the evaluation aims to assess the outcomes of the implementation strategy and the outcomes of each intervention. With respect to the latter, the evaluation aims to unpack how the co-creation initiative has unfolded in practice and its impact on local policy-making around care and support for people with dementia.

PLEASE INSERT FIGURE 1 ABOUT HERE

### *The intervention and the implementation strategy*

A fundamental unmet need for information was reported by people with dementia and family carers who took part in the diagnostic phase. Local stakeholders agreed to tackle this issue by providing up-to-date, accessible and timely information about local services available to those living with dementia and family carers. Figure 2 provides a timeline of the process underpinning the development of the agreed intervention.

PLEASE INSERT FIGURE 2 ABOUT HERE

A local working group (including commissioners, statutory service providers, third sector organisations providing support and care to people with dementia and family carers, a professional designer, alongside the research team) was set up with the remit of developing an information leaflet and planning its implementation. Efforts to involve family carers and people with dementia by asking local third sector organizations to identify people keen to be involved in the working group resulted in one family carer joining the group. Another turned down the offer because of competing commitments and no-one living with dementia was



For peer review only - http://bmjopen.bmj.com/site/about/guidelines.xhtml

identified. To mitigate their absence, inputs from front line staff providing daily direct support and care to people with dementia and to family carers were used (one was also a family carer). In a similar vein, we relied also on the professional designer who had extensive experience and expertise of designing information material intended to be used by people with cognitive impairment.

The group produced three prototypes which were shared and discussed in a consultation exercise involving about 20 among people with dementia and family carers. The consultation exercise was planned around the schedule of activities or groups organized locally for people with dementia and/or family carers. This solution overcame some of the practical problems that might have limited their participation in the working group.

The preferred prototype was iteratively refined using best practice guidance on Patient Information Leaflets[22] and taking into account feedback provided by its potential users, commissioners and service providers. Following local discussion, it was agreed to design only one version of the leaflet for both family caregivers and individuals living with dementia. The leaflet was intended to cover essential information about, and contact details of, the key local organizations that provide dementia-related support and services, which, in practice, often cater for both groups. The intention was to signpost people to such organizations, rather than providing an exhaustive list of services offered. Hence, two versions of the leaflet would not have added substantially different content but could have contributed to information overload, sometimes experienced by those living with dementia and their families (for example around the time of receiving the diagnosis). In terms of graphics, the design aimed to maximize accessibility, irrespective of the intended user's group. From the implementation perspective, it was felt that it would be easier to embed one version of the leaflet in the practice of professionals and, equally, designing and using one version of the leaflet could, more likely, contribute to its sustainability in the longer term, for example by simplifying the updating process.

In its final version, the leaflet presents services clustered around four main themes ("I want to speak to someone for advice and support", "I want to live safely and with the right support", "I want to know what to do in a crisis", "Where can I go for more information?") and it can be printed out on a two-sided A4 using a high contrast colour combination.

Subsequently, the working group agreed a phased implementation process: the Community Mental Health Services for Older People (CMHSOP) within the local mental health trust were identified as the first implementation route because of their key role in diagnosing dementia and in the resulting post-diagnostic care planning. Following diagnosis and initial care planning, those whose needs are stable are discharged to primary care while those with more complex needs continue to receive secondary services.

The working group preliminarily explored with the CMHSOP service manager, team managers and representatives of the Community Psychiatric Nurses (CPNs) how the leaflet could be used in clinical practice. In January 2019, following positive and supportive feedback, the leaflet working group members and the CMHSOP service manager, team managers and two CPNs took part in a focus group aimed at articulating the theory of change associated with the intervention itself. This exercise helped making explicit the expected process of implementing the intervention in practice and its anticipated impact. Mobilisation activities (e.g. information sessions within team meetings and regular reminders emailed to team managers and cascaded to CPNs) were planned and carried out to inform, and subsequently to remind, all CPNs about the leaflet and its use in practice. In June 2019, all



For peer review only - http://bmjopen.bmj.com/site/about/guidelines.xhtml

the CPNs across the four locality teams covering the study site were invited to start using the leaflet in their clinical practice.

CPNs agreed to hand out the leaflet to patients on their caseload who received a diagnosis of any type of dementia and/or to their family carers. CPNs were expected to use their clinical judgement in deciding to whom and when to offer the leaflet, dependent upon the patient's readiness to receive the leaflet and their individual and family circumstances. They expected to hand it out during their encounter with patients and families, when they felt that the leaflet would support their conversations with them. For these reasons, the use of the leaflet was not embedded at a specific point of the clinical pathway.

A detailed description of the intervention and its implementation strategy are outlined in accordance with the StaRI framework respectively in Table 1 and Table 2 (item 9).

For peer review only

**Table 1 – Overview of the implementation strategy reported in accordance with the StaRI checklist**

| IMPLEMENTATION STRATEGY (i.e. how the intervention was implemented) | | |
|---|---|---|
| **Item** | **Description and reference to methodological frameworks and reporting standards** | |
| 1. Title | | Effectiveness-implementation hybrid type 2 study evaluating an intervention to support 'information work' in dementia care |
| 2. Abstract | | See Abstract of the protocol |
| 3. Introduction | To identify the problem | See Introduction of the protocol |
| 4. Introduction | Rationale for the implementation strategy and the intervention | See Intervention development of the protocol |
| 5. Aims and objectives | | 1. To assess the unfolding of the implementation strategy and its outcomes<br>2. To reflect on the extent to which, how and why the co-creation initiative has influenced the design of the implementation strategy and its unfolding and outcomes |
| 6. The design and key features of the evaluation | | The evaluation of the implementation strategy aims at understanding the feasibility of the intervention by investigating how the actual implementation process has unfolded in practice and identifying any discrepancies between the planned and the actual implementation process |
| 7. Context | Context in which the intervention is implemented | Community mental health services for older people (CMHSOP) within a mental health foundation trust in the North East of England (UK)<br>- North East of England is an under-researched area compared to other parts of England<br>- Local health economy with average recorded diagnosed dementia prevalence (for 2018-2019, local recorded prevalence rate 0.9% vs national recorded prevalence rate 0.8%) and average recorded annual review rate (for 2018-2019, local annual review rate 79.2% vs national recorded prevalence rate 77.9%)[23]<br>- Mental health foundation trust, established in 2006 providing a range of mental health, learning disability and eating disorder services for around two million people across a large and diverse geographical area<br>- CMHSOP provide community-based services for older people experiencing cognitive impairment and mental health problems. Organized around four localities, each locality team includes community psychiatric nurses (CPNs), occupational therapists, physiotherapists, psychologists, support workers to provide multi-disciplinary support<br>Intervention was developed in the context of a 5-year action research co-creation initiative funded by an English charity, involving researchers from three English universities and a research facilitator from the local commissioning support unit alongside a constellation of local stakeholders |



For peer review only - http://bmjopen.bmj.com/site/about/guidelines.xhtml

<table>
<tr><th colspan="3">IMPLEMENTATION STRATEGY (i.e. how the intervention was implemented)</th></tr>
<tr><th>Item</th><th>Description and reference to methodological frameworks and reporting standards</th><th></th></tr>
<tr><td>8. Target sites</td><td></td><td>Four locality teams, staffing levels and caseload are reported in the table below<br><br><table><tr><th>Team</th><th>Total staff</th><th>CPNs</th><th>Caseload</th></tr><tr><td>Team 1</td><td>48</td><td>9</td><td>1,368</td></tr><tr><td>Team 2</td><td>43</td><td>8</td><td>1,377</td></tr><tr><td>Team 3</td><td>27</td><td>5</td><td>787</td></tr><tr><td>Team 4</td><td>26</td><td>5</td><td>791</td></tr></table></td></tr>
<tr><td>9. Implementation strategy</td><td>Description of the implementation strategy in accordance with Proctor et al.’s framework for reporting implementation strategies[24]</td><td>Name and definition of the implementation strategy: bundle of professional and material interventions aiming at the development, adoption and use of the dementia leaflet among CPNs (based on framework by [25,26]<br><br>Specification of the implementation strategy<br>- Actors: Community Psychiatric Nurses (CPNs) and team managers and service manager across four CMHSOP teams<br>- Actions: Described in accordance with relevant phase of the Plan-Do-Study-Act (PDSA) framework<br>  - Plan:<br>    Professional interventions<br>    o Established a local working group in charge of overseeing the development the leaflet and its implementation strategy<br>    o Iteratively developed a dementia leaflet, embedding feedback from people with dementia, family carers, professionals from local organisations providing care and support to people with dementia and family carers<br>    o Identified the implementation strategy via CPNs based in CMHSOP, identified local champion (service manager), identified implementation sites<br>    o Organised mobilisation activities with implementation sites, including information sessions at team meetings and email reminders<br>    Material intervention<br>    o Printed copies of the leaflet made available to implementers</td></tr>
</table>

For peer review only

| IMPLEMENTATION STRATEGY (i.e. how the intervention was implemented) | | |
|---|---|---|
| **Item** | **Description and reference to methodological frameworks and reporting standards** | |
| | | - Do: CPNs offer a copy of the leaflet to people with dementia or their family carers attending an appointment (any type)<br>- *Action targets*: People living with dementia (any type of dementia, diagnosed at any time) and their family carers attending an appointment with their CPNs. CPNs were expected to use their clinical judgment in identifying patients or family members deemed to be ready to receive the leaflet<br>- *Temporality*: The leaflet can be offered at any point during the period in which the patient was under the care of the CMHSOP (at initial assessment, at post diagnosis, at reviews, at discharge)<br>- *Dose:* Depending on the individual readiness to receive the leaflet and individual and family circumstance, CPNs decide whether to offer or not the leaflet as part of their appointment with the patient. The leaflet could be offered more than once at different points in time |
| 10. Sub-groups | Any subgroups recruited for additional research tasks, and/or nested studies | Not applicable to this study |
| **Evaluation** | | |
| 11. Outcomes | Using Proctor et al.'s taxonomy of outcomes for implementation research [27] | - Acceptability of the intervention by CPNs: perception among CPNs that the intervention is agreeable, palatable, or satisfactory<br>- Adoption of the intervention by CPNs: CPNs' intention, initial decision, or action to try the intervention<br>- Appropriateness of the intervention implemented as judged by CPNs: perceived fit, relevance, or compatibility of the intervention for the setting of CMHSOP and perceived fit of the intervention to address the issue<br>- Cost of the intervention for the implementers<br>- Feasibility of the implementation of the intervention: the extent to which the intervention can be successfully used or carried out within the setting of the CMHSOP<br>- Penetration of the intervention: integration of the intervention within the CMHSOP<br>- Sustainability of the intervention: extent to which the intervention is maintained or institutionalised within the CMHSOP |
| 12. Process evaluation objectives and outcomes related to the mechanism(s) through which the strategy is expected to work | Objectives and outcomes related to the mechanism by which the strategy is expected to work, using MRC framework for complex interventions [28] | - Fidelity of the intervention: adherence to the intervention protocol<br>- Adaptations of the intervention<br>- Dose: the quantity of intervention implemented<br>- Reach: whether the intended audience comes into contact with the intervention, and how |



For peer review only - http://bmjopen.bmj.com/site/about/guidelines.xhtml

| IMPLEMENTATION STRATEGY (i.e. how the intervention was implemented) | | |
|---|---|---|
| **Item** | **Description and reference to methodological frameworks and reporting standards** | |
| 13. Data collection methods | | In-depth interviews with CPNs, team managers and service manager of CMHSOP |
| 14. Sample size | | - Up to 12 CPNs from four locality teams<br>- Four team managers<br>- One service manager |
| 15. Methods of analysis | | Thematic content analysis of interview data |

For peer review only



For peer review only - http://bmjopen.bmj.com/site/about/guidelines.xhtml

**Table 2 – Overview of the intervention reported in accordance with the StaRI checklist**

| INTERVENTION (i.e. the actual intervention being implemented) | | |
|---|---|---|
| **Item** | **Description and reference to methodological frameworks and reporting standards** | |
| 1. Title | | Effectiveness-implementation hybrid type 2 study evaluating an intervention to support 'information work' in dementia care |
| 2. Abstract | | See Abstract of the protocol |
| 3. Introduction | To identify the problem | See Introduction of the protocol |
| 4. Introduction | Rationale for the implementation strategy and the intervention | See Intervention development of the protocol |
| 5. Aims and objectives | | 1. To assess the outcomes of the intervention as perceived by people living with dementia and their family carers, staff implementing the intervention and local organizations and to explain how and why the intervention contributed to achieve these outcomes<br>2. To reflect on the extent to which, how and why the co-creation initiative has influenced the design, implementation and impact of the intervention |
| 6. The design and key features of the evaluation | | The evaluation of the intervention aims at assessing the outcomes of the intervention with respect to people with dementia and family carers (primary outcomes), to the implementers of the intervention (proximal outcomes) and to local service providers (distal outcomes) |
| 7. Context | Context in which the intervention was implemented | As per implementation strategy described above |
| 8. Target population | Characteristics of the population targeted by the intervention and eligibility criteria | People living with dementia (any type of dementia, diagnosed at any time) and their family carers attending an appointment with their CPNs. CPNs were expected to use their clinical judgment in identifying patients or family members deemed to be ready to receive the leaflet |
| 9. Intervention | Description of the intervention, provided in accordance with the TIDieR checklist [29] | Name: Leaflet about local care and support available to people living with dementia and family carers<br><br>Why: in the diagnostic phase of the co-creation initiative, people living with dementia and family carers consistently reported a lack of timely, up-to-date information provided in a user-friendly format which hinders their 'information work'. The intervention has been designed to provide a short, up-to-date, paper-based source of information that could be handed out and used by CPNs in their encounter with people with dementia and their family carers and support their 'information work' |



For peer review only - http://bmjopen.bmj.com/site/about/guidelines.xhtml

| INTERVENTION (i.e. the actual intervention being implemented) | | |
|---|---|---|
| **Item** | **Description and reference to methodological frameworks and reporting standards** | |
| | | What (materials): An A4 three-folded, two-sided leaflet printed in high contrast colour combination. Information are organized in four themes, for which relevant services, a short description and contact details are provided<br><br>What (procedures): CPNs offer the leaflet to patients and their family carers as part of their clinical encounters<br><br>Who: 27 qualified mental health nurses (CPNs) part of the organic cells working across four locality teams of the local CMHSOP who assess, diagnose, review medications and offer post-diagnostic support to people with dementia and signpost family carers to services available to them<br><br>How: the leaflet is offered in the context of the appointment attended by the person with dementia and their family carer. The CPN can offer the leaflet in response to a current specific need of the person with dementia or of the family carer (either explicitly reported or latent) or opportunistically, to make them aware of local services which could be of interest either at the present time or in the future<br><br>Where: the intervention takes place in the outpatient clinics of the CMHSOP services of the local mental health foundation trust<br><br>When and how much: CPNs can offer the leaflet at any point in the period when the patient is under the care of the CMHSOP (e.g. at initial assessment, at diagnosis, in the post diagnostic appointment, at review, upon transfer of care back to GP). It could be offered by the CPNs more than once at different points in time<br><br>Tailoring: CPNs are expected to use their clinical judgement in deciding whether, when, how and to whom to offer the leaflet based on<br>- patients or families' readiness to accept the leaflet (e.g. some patients do not temporarily - or will never - accept a diagnosis of dementia)<br>- length of the appointments (e.g. initial assessment can take up to 90 minutes and patients and families may be overloaded with information)<br>- type of appointments (e.g. titration appointments may cause disappointment in patients) |



For peer review only - http://bmjopen.bmj.com/site/about/guidelines.xhtml

| INTERVENTION (i.e. the actual intervention being implemented) | | |
|---|---|---|
| **Item** | **Description and reference to methodological frameworks and reporting standards** | |
| 10. Sub-groups | Any subgroups recruited for additional research tasks, and/or nested studies | Not applicable to this study |
| **Evaluation** | | |
| 11. Outcomes | Primary and other outcomes of the intervention | For people with dementia and family carers (primary outcomes)<br>- their satisfaction with the availability of information on local services (e.g. in terms of timing and quality of information)<br>- their awareness of local services<br>- in the longer term, their access and use of local services<br><br>For implementers of the intervention (proximal outcomes)<br>- CPN, team and service managers' awareness of local services and support available to people diagnosed with dementia and to their family carers<br>- CPN, team and service managers' confidence to hold conversations around using services available to those living with dementia and their family carers<br><br>For local service providers (distal outcomes)<br>- volume of referrals into their services<br>- appropriateness of the use of services among those living with dementia and family carers (e.g. with respect to the type and level of need) |
| 12. Process evaluation objectives and outcomes related to the mechanism(s) through which the strategy is expected to work | Objectives and outcomes related to the mechanism by which the strategy is expected to work, using MRC framework for complex interventions [28] | As per implementation strategy |
| 13. Data collection methods | | People with dementia and family carers<br>- Longitudinal semi-structured in-depth interviews: interview 1 carried out 4-6 weeks after the appointment with the CPN when they receive the leaflet; interview 2 (follow-up) scheduled about 6 months after interview 1<br><br>Implementers of the intervention<br>- Semi-structured in-depth interviews with CPNs, team managers and service manager of CMHSOP (scheduled about 6 months from the start of the implementation of the intervention) |



For peer review only - http://bmjopen.bmj.com/site/about/guidelines.xhtml

| INTERVENTION (i.e. the actual intervention being implemented) | | |
|---|---|---|
| **Item** | **Description and reference to methodological frameworks and reporting standards** | |
| | | Local service providers<br>- Activity log consisting of a table recording the monthly number of referrals received by the provider in total, by user group (people with dementia or family carer), by referral source (i.e. self-referral, CMHSOP, social services, other), by reason of the referral (e.g. health and safety assessment, information, support for carer, support for people with dementia), over a 24-month period (i.e. in the 12 months preceding the implementation of the intervention and during the 12-month implementation period)<br>- Semi-structured in-depth interviews with managers (scheduled about 10 months from the start of the implementation of the intervention)<br><br>Local working group<br>- focus group with about 12 participants (including commissioners, statutory service providers, staff from CMHSOP, managers from third sector organizations, a family carer) (scheduled about 12 months from the start of the implementation of the intervention) |
| 14. Sample size | | People living with dementia and family carers<br>- Up to 30 people living with dementia (any type of dementia, diagnosed at any time, willing and able to consent) and family carers (of somebody with any type of dementia, any degree of severity, diagnosed at any time)<br><br>Implementers of the intervention<br>- Up to 12 CPNs from four locality teams<br>- Four team managers<br>- Service manager<br><br>Local service providers<br>- at least three local organisations out of the seven listed on the leaflet as service providers which in preliminary conversations committed to supply data to inform the evaluation<br><br>Local working group<br>- about 12 participants attending the focus group (including commissioners, statutory service providers, staff from CMHSOP, managers from third sector organizations, a person with dementia and a family carer) |

For peer review only

| INTERVENTION (i.e. the actual intervention being implemented) | | |
|---|---|---|
| **Item** | **Description and reference to methodological frameworks and reporting standards** | |
| 15. Methods of analysis | | Thematic content analysis of interview and focus group data<br>Descriptive statistics for referral data provided by local service providers, triangulated with interview data |

For peer review only



For peer review only - http://bmjopen.bmj.com/site/about/guidelines.xhtml

## METHODS AND ANALYSIS

### *Evaluation framework*

The study adopts an effectiveness-implementation hybrid type 2 design which allows simultaneous evaluation of the intervention and its implementation strategy,[30] as reflected in the evaluation framework (Figure 3).

PLEASE INSERT FIGURE 3 ABOUT HERE

The evaluation framework maps the phases of the study (design – implementation – evaluation) on to the key components of the intervention and shows the relations among them. In the design phase, the intervention and the implementation strategy (i.e. the bundle of professional and material interventions intended to sustain the adoption and use of the leaflet among CPNs) were articulated with the theory of change focus group. Following the implementation, the evaluation will

1. assess the unfolding of the implementation strategy and its outcomes
2. assess the outcomes of the intervention as perceived by people with dementia and family carers, the implementers of the intervention, as well as the providers of local services available to people with dementia and their families
3. identify the mechanisms of action, i.e. the “active ingredients” that ignited the implementation strategy and the intervention and, hence, contributed to the observed outcomes
4. reflect on the contribution of context (e.g. the social, economic, policy and organisational factors surrounding the intervention, including, but not limited to, the co-creation initiative, as described in Table 1, item 7) to the design and implementation of the intervention and its implementation strategy and relative outcomes

Outcomes of the implementation strategy

The implementation strategy is evaluated with respect to both its outcomes and its process dimension, as per the StaRI framework. Following Proctor et al.’s taxonomy,[27] the following outcomes will be assessed with respect to the implementation strategy (Table 1, item 11):

- Acceptability of the intervention by CPNs: perception among CPNs that the intervention is agreeable, palatable, or satisfactory
- Adoption of the intervention by CPNs: CPNs’ intention, initial decision, or action of trying out the intervention
- Appropriateness of the intervention implemented as judged by CPNs: perceived fit, relevance, or compatibility of the intervention for the setting of the CMHSOP and perceived fit of the intervention to address the issue
- Cost of the intervention for the implementers
- Feasibility of the implementation of the intervention: the extent to which the intervention can be successfully used or carried out within the setting of the CMHSOP



For peer review only - http://bmjopen.bmj.com/site/about/guidelines.xhtml

- Penetration of the intervention: the extent to which the intervention is integrated into the practice of the CMHSOP
- Sustainability of the intervention: extent to which the intervention is maintained or embedded within the CMHSOP

Outcomes of the intervention

The theory of change exercise run in January 2019 highlighted that the intervention was expected to have an impact on (Table 2, item 11):

- Its main target population, i.e. people with dementia and family carers (direct outcomes)
- The implementers of the intervention, i.e. CPNs, team and service managers of the CMHSOP delivering the intervention (proximal outcomes)
- Local providers of care and support to people with dementia and their families, such as local third sector organisations (distal outcomes)

With respect to people with dementia and family carers, the intervention was expected to improve three outcomes directly associated with the intervention: (i) their satisfaction with the availability of information on local services (e.g. in terms of timing and quality of information), (ii) their awareness of local services and (iii) in the longer term, their access and use of local services.

With respect to the implementers, the intervention was expected to influence two proximal outcomes: (i) CPN, team and service manager's awareness of local services and support available to people diagnosed with dementia and to their family carers and (ii) CPN, team and service manager's confidence to hold conversations with people living with dementia and their family carers about seeking support and using local services.

With respect to local service providers, the intervention was expected to impact on two distal outcomes: (i) volume of referrals into their services and (ii) appropriateness of the use of services among those living with dementia and family carers (e.g. with respect to the type and level of need).

Process evaluation

The MRC framework for process evaluation[28] is used to investigate (Table 1 and Table 2, item 12)

- the actual implementation strategy, in terms of implementation fidelity (or, conversely adaptation), reach and dose,
- the mechanisms of action of the implementation strategy and of the intervention which brought about change

The process evaluation will highlight how the implementation process has unfolded in practice and any discrepancies between the actual implementation process and how it was articulated in the theory of change. Moreover, it will help to surface how and why the intervention and its implementation strategy have contributed to achieve these outcomes.



For peer review only - http://bmjopen.bmj.com/site/about/guidelines.xhtml

### *Analytical framework*

The framework underpinning the realist evaluation will be adopted as analytical tool. Realist evaluation acknowledges that interventions work (have successful outcomes) in so far as they introduce appropriate ideas and opportunities (mechanisms) to groups of stakeholders in the appropriate social and cultural conditions (context), as condensed in the following formula [31]

$$\text{mechanism} + \text{context} = \text{outcome}$$

According to the realist framework, mechanisms are the underlying processes or hidden causal levers of intervention activities that make them work[32] and are represented by the psychological or social explanations of behaviour of intervention participants and their ideas about how change is achieved.[33] Context refers to the conditions likely to enable or constrain the activation of interventions' mechanisms (e.g. the organisational setting or the broader policy and institutional environment in which an intervention is embedded). Outcome is the anticipated and unanticipated consequence that is brought about by the interaction of different interventions' mechanisms in different contexts.

The realist framework will be overlaid with Wilson's information seeking behaviour model, a user-centred approach grounded in information science, which contends that any given information-seeking process is shaped by two crucial factors, the context in which it occurs and its activating mechanisms.[18] The model acknowledges that information need, seeking and use is situated and may be affected by its context, encompassing broader environmental factors (e.g. political, eco- nomic, technological) as well as the personal characteristics of those who seek information (e.g. demographic characteristics or psychological disposition) and their social roles (e.g. being a patient or a carer).[17] Activating mechanisms are the factors that prompt or discourage information seeking and motivate the sources used (e.g. stress and coping, which may explain why some do and others do not seek information; risk and reward, which relate to the role of information sources in addressing the uncertainty associated with a situation and may help explain which sources may be used in a given context; self-efficacy, i.e. the individual confidence to initiate the process of seeking information and to act upon it).[17,34] Both concepts will be used to operationalize their realist analogues in the analytical framework.

### *Data collection methods and samples*

The mixed-method evaluation will build on data collected from four sets of stakeholders involved in the intervention (Table 1 and Table 2, items 13 and 14):

- people living with dementia and family carers who are at the immediate receiving end of the intervention
- CPNs, team and service managers based in the CMHSOP who are implementing the intervention
- local providers of care and support to people with dementia and their families, as the organisations expected to observe a change in their service access and use as a consequence of the intervention
- local working group, who contributed to the development of the intervention



For peer review only - http://bmjopen.bmj.com/site/about/guidelines.xhtml

Figure 4 provides an overview of the data collection timeline.

PLEASE INSERT FIGURE 4 ABOUT HERE

People with dementia and family carers

Semi-structured in-depth interviews with up to 30 people living with dementia (any type of dementia, diagnosed at any time, willing and able to consent) and family carers (of somebody with any type of dementia, any degree of severity, diagnosed at any time) will take place at two points in time: interview 1 will be carried out 4-6 weeks after the appointment with the CPN when they receive the leaflet and interview 2 (follow up) will be scheduled about 6 months after interview 1.

The two timepoints have been established to best capture participants' experiences of receiving (interview 1) and using (follow up interview) the leaflet (the interview guides are provided as Supplementary file). Accordingly, interview 1 will aim to understand (i) if, when and how they received the leaflet; (ii) whether and how they anticipate using the leaflet; (iii) what they think of the leaflet overall (e.g. whether it has changed their perceptions and awareness of the local services offering care and support to those living with dementia and their family carers). Interview 2 will aim to understand (i) whether, how, when and why they used (or not) the leaflet; (ii) whether and how the leaflet has changed their care seeking behaviour; (iii) their satisfaction with the leaflet.

Longitudinal interviews with people living with dementia and their family carers are a well-established research method.[35–39] The follow up period is appropriate for research purposes. Over a period of 6 months, personal and family circumstances can change and this may prompt those living with dementia and their family carers to seek help and support. Similarly, those recently diagnosed may not feel immediately ready to get in touch with local services but this could change after 6 months.

This purposeful sample will be recruited when they attend their first appointment with the CPN. The size of the sample is expected to allow to capture a wide range of perspectives (varying for roles, i.e. people with dementia vs family carers, types of dementia diagnosis, levels of cognitive impairment, age, gender and living arrangements) while achieving data saturation. The recruitment will be carried out on a rolling basis alongside the implementation of the intervention and will stop when the desired sample size is reached.

Community Mental Health Services for Older People (CMHSOP)

In-depth interviews with CPNs, team and service managers from the implementing CMHSOP will be carried out about 6 months from the start of dissemination of the leaflet. Up to 20 staff members will be recruited across the 4 localities in which the CMHSOP operate.

The interviews aim to understand (i) whether, when and how they used (or not) the leaflet in clinical practice; (ii) whether and how the leaflet has changed any aspect of their clinical practice; (iii) their views about the impact of adopting the principles of co-creation on the implementation and impact of the intervention. The interview guides are provided as Supplementary file.



For peer review only - http://bmjopen.bmj.com/site/about/guidelines.xhtml

Local service providers

Referral data from a minimum of three out of the seven local organisations listed on the leaflet which provide care and support to people living with dementia and their family carers will be collected for a period of 24 months (in the 12 months preceding the implementation and for the 12-month implementation period). Semi-structured in-depth interviews with service managers will be undertaken about 10 months from the start of the dissemination of the leaflet to (i) review the referral data and (ii) identify and interpret the patterns of referral into each service (e.g. over time, with respect to the referral source or the user group) and their appropriateness (e.g. with respect to the type and level of need). The interview guide is provided as Supplementary file.

Local working group

A focus group with the local working group (about 12 participants including commissioners, statutory service providers, staff from CMHSOP, managers from third sector organizations, a family carer) will be held towards the end of the implementation period, following preliminary analysis of the evaluation data (see the Supplementary file for the focus group guide). This will aim to

- discuss and validate the emerging findings about the evaluation of the implementation strategy
- discuss and validate the emerging findings around the impact of the intervention on people with dementia and their families, CPNs and mental health services and local service providers
- reflect on the impact of co-creation initiative on the implementation and impact of the intervention

***Informed consent***

Written and verbal versions of the Participant Information Sheet and Informed Consent Sheet will be presented to participants. The Participant Information Sheet details the nature of the study, what participation will involve, any risks involved. It will be clearly stated that the participant is free to withdraw at any time, for any reason without impact on future care or service access, and with no obligation to give the reason for withdrawal. Participants will be allowed as much time as they wish to consider the information (at least 48 hours), and will have the opportunity to ask questions of the Investigator, their GP and/or CPNs (for people with dementia and family carers), or other independent parties in order to decide whether they will participate in the study. Written informed consent will be obtained.

Maximum efforts will be devoted to recruit people living with dementia into this study. Historically, people with dementia have been excluded from much research in social and health care on the basis that their declining cognitive abilities may affect capacity to consent.[40] However, the ability of many people with dementia to provide informed consent, based on their ability to understand information, evaluate risks and benefits, and communicate decisions effectively has been established.[41,42] Prior to the interview, an informal conversation about the research will allow the researchers to ascertain that the potential participant has capacity to consent. If the researcher believes that the person with dementia has capacity, she will take written informed consent and will start the interview.



For peer review only - http://bmjopen.bmj.com/site/about/guidelines.xhtml

### *Data analysis*

The referral data provided by each local provider will be collated, tabulated and described with simple metrics (e.g. number of referrals in total, by user group, by referral source, by reason for referral). The results will be triangulated with interview data collected from the participants living with dementia and family carers, the implementers and the managers of the local service providers.

The interviews will be transcribed, anonymised and analysed using thematic analysis (performed with NVivo12) (Table 1 and Table 2, item 15).[43] Following the realist approach, the initial codebook will be developed around the themes (codes) of context conditions, underlying mechanisms, observed outcomes matched on to implementation strategy and intervention. These will be then refined into sub-codes to capture specificities of data collected from different interviewee roles or to highlight different dimensions featuring in a main code. For example, for intervention strategy, the sub-codes of "fidelity", "dose", "adaptations", "reach" may be developed or "mechanisms" may be clustered around the sub-codes "self-efficacy" and "resources".

### *Patient and Public Involvement*

A person living with dementia and a family carer of somebody living with dementia are members of the advisory board of the co-creation initiative. In this capacity they are overseeing the development of the study, provided feedback in the design phase of the intervention, reviewed the interview guides and are expected to review the results.

## ETHICS AND DISSEMINATION

Ethical and research governance approvals have been obtained from the West Midlands - South Birmingham Research Ethics Committee [REC reference 16/WM/0397].

The study has been designed as part of a 5-year co-creation initiative by the local working group in collaboration with the research team. Regular updates about the implementation of the intervention are regularly shared with the working group and with the wider group of stakeholders involved in the co-creation initiative.

Findings of the work will be disseminated across professionals e.g. at national conferences and events for providers and commissioners of care and support for people with dementia, policy makers in the area of dementia, representatives of services users. Articles stemming from this work will be submitted for publication to peer-reviewed journals and to national and international scientific conferences in the field of applied health research.

## REFERENCES

1 Dalmer NK. 'Add info and stir': an institutional ethnographic scoping review of family care-givers' information work. *Ageing Soc* 2018;:1–27. doi:10.1017/S0144686X18001125



For peer review only - http://bmjopen.bmj.com/site/about/guidelines.xhtml

2 Harris R, Wathen N, Wyatt S. Working to be healthy: empowering consumers or citizens? In: Harris R, Wathen N, Wyatt S, eds. *Configuring Health Consumers: Health Work and the Imperative of Personal Responsibility*. New York, NY: : Palgrave Macmillan 2010. 211–24.

3 Washington KT, Meadows SE, Elliott SG, *et al.* Information needs of informal caregivers of older adults with chronic health conditions. *Patient Educ Couns* 2011;**83**:37–44. doi:10.1016/j.pec.2010.04.017

4 Mastel-Smith B, Stanley-Hermanns M. 'It's like we're grasping at anything': Caregivers' education needs and preferred learning methods. *Qual Health Res* 2012;**22**:1007–15. doi:10.1177/1049732312443739

5 Dunbrack J. The information needs of informal caregiversinvolved in providing support to a critically ill loved one. 2005.

6 Vaingankar JA, Subramaniam M, Picco L, *et al.* Perceived unmet needs of informal caregivers of people with dementia in Singapore. *Int Psychogeriatrics* 2013;**25**:1605–19. doi:10.1017/S1041610213001051

7 Prorok J, Horgan S, Seitz D. Health care experiences of people with dementia and their caregivers: a meta-ethnographic analysis of qualitative studies. *CMAJ* 2013;**185**:E669-80.

8 Greenwood N, Habibi R, Smith R, *et al.* Barriers to access and minority ethnic carers' satisfaction with social care services in the community: A systematic review of qualitative and quantitative literature. *Heal Soc Care Community* 2015;**23**:64–78. doi:10.1111/hsc.12116

9 Afram B, Verbeek H, Bleijlevens MHC, *et al.* Needs of informal caregivers during transition from home towards institutional care in dementia: A systematic review of qualitative studies. Int. Psychogeriatrics. 2015;**27**:891–902. doi:10.1017/S1041610214002154

10 Sutcliffe CL, Roe B, Jasper R, *et al.* People with dementia and carers' experiences of dementia care and services: Outcomes of a focus group study. *Dementia* 2015;**14**:769–87. doi:10.1177/1471301213511957

11 Downs M, Clibbens R, Rae C, *et al.* What Do General Practitioners Tell People with Dementia and their Families about the Condition? *Dementia* 2002;**1**:47–58. doi:10.1177/147130120200100106

12 Van Hout HP, Vernooij-Dassen MJ, Jansen DA, *et al.* Do general practitioners disclose correct information to their patients suspected of dementia and their caregivers? A prospective observational study. *Aging Ment Heal* 2006;**10**:151–5. doi:10.1080/13607860500310468

13 Allen F, Cain R, Meyer C. Seeking relational information sources in the digital age: A study into information source preferences amongst family and friends of those with dementia. Dementia. 2018. doi:10.1177/1471301218786568

14 Wackerbarth SB, Johnson MMS. Essential information and support needs of family caregivers. *Patient Educ Couns* 2002;**47**:95–100. doi:10.1016/S0738-3991(01)00194-X

15 Koehn S. Negotiating candidacy: ethnic minority seniors' access to care. *Ageing Soc* 2009;**29**:585–608. doi:10.1017/S0144686X08007952



For peer review only - http://bmjopen.bmj.com/site/about/guidelines.xhtml

16 Kelly F, Innes A. Facilitating independence: The benefits of a post-diagnostic support project for people with dementia. *Dementia* 2016;**15**:162–80. doi:10.1177/1471301214520780

17 Wilson TD. Models in information behaviour research. *J Doc* 1999;**55**:249–70. doi:10.1108/EUM0000000007145

18 Harland JA, Bath PA. Understanding the information behaviours of carers of people with dementia: A critical review of models from information science. Aging Ment. Heal. 2008;**12**:467–77. doi:10.1080/13607860802224300

19 Pinnock H, Barwick M, Carpenter CR, *et al.* Standards for Reporting Implementation Studies (StaRI) Statement. *BMJ* 2017;**356**:i6795. doi:10.1136/bmj.i6795

20 Pinnock H, Barwick M, Carpenter CR, *et al.* Standards for Reporting Implementation Studies (StaRI): explanation and elaboration document. *BMJ Open* 2017;**7**:e013318. doi:10.1136/bmjopen-2016-013318

21 Susman G, Evered R. An assessment of the scientific merits of action research. *Adm Sci Q* 1978;**23**:582–603.

22 Sustersic M, Gauchet A, Foote A, *et al.* How best to use and evaluate Patient Information Leaflets given during a consultation: a systematic review of literature reviews. *Heal Expect* 2017;**20**:531–42. doi:10.1111/hex.12487

23 NHS Digital. QOF 2018/19. https://qof.digital.nhs.uk/ (accessed 6 Mar 2020).

24 Proctor EK, Powell BJ, McMillen JC. Implementation strategies: recommendations for specifying and reporting. *Implement Sci* 2013;**8**:139. doi:10.1186/1748-5908-8-139

25 Mazza D, Bairstow P, Buchan H, *et al.* Refining a taxonomy for guideline implementation: results of an exercise in abstract classification. *Implement Sci* 2013;**8**:32. doi:10.1186/1748-5908-8-32

26 Powell BJ, McMillen JC, Proctor EK, *et al.* A compilation of strategies for implementing clinical innovations in health and mental health. Med. Care Res. Rev. 2012;**69**:123–57. doi:10.1177/1077558711430690

27 Proctor E, Silmere H, Raghavan R, *et al.* Outcomes for implementation research: conceptual distinctions, measurement challenges, and research agenda. *Adm Policy Ment Health* 2011;**38**:65–76. doi:10.1007/s10488-010-0319-7

28 Moore GF, Audrey S, Barker M, *et al.* Process evaluation of complex interventions: Medical Research Council guidance. *BMJ* 2015;**350**:h1258. doi:10.1136/bmj.h1258

29 Hoffmann TC, Glasziou PP, Boutron I, *et al.* Better reporting of interventions: Template for intervention description and replication (TIDieR) checklist and guide. *BMJ* 2014;**348**. doi:10.1136/bmj.g1687

30 Curran GM, Bauer M, Mittman B, *et al.* Effectiveness-implementation hybrid designs: combining elements of clinical effectiveness and implementation research to enhance public health impact. *Med Care* 2012;**50**:217–26. doi:10.1097/MLR.0b013e3182408812

31 Pawson R, Tilley N. *Realistic Evaluation*. Thousand Oaks, CA: : SAGE publications 1997.

32 Astbury B, Leeuw FL. Unpacking Black Boxes: Mechanisms and Theory Building in Evaluation. *Am J Eval* 2010;**31**:363–81. doi:10.1177/1098214010371972



For peer review only

33 Greenhalgh T, Humphrey C, Hughes J, *et al.* How do you modernize a health service? A realist evaluation of whole-scale transformation in london. *Milbank Q* 2009;**87**:391–416. doi:10.1111/j.1468-0009.2009.00562.x

34 Case DO, Andrews JE, Johnson JD, *et al.* Avoiding versus seeking: The relationship of information seeking to avoidance, blunting, coping, dissonance, and related concepts. J. Med. Libr. Assoc. 2005;**93**:353–62.

35 Johannessen A, Engedal K, Haugen PK, *et al.* "To be, or not to be": experiencing deterioration among people with young-onset dementia living alone. *Int J Qual Stud Health Well-being* 2018;**13**:1490620. doi:10.1080/17482631.2018.1490620

36 Samsi K, Manthorpe J. Everyday decision-making in dementia: findings from a longitudinal interview study of people with dementia and family carers. *Int Psychogeriatrics* 2013;**25**:949–61. doi:10.1017/S1041610213000306

37 Windle G, Joling KJ, Howson-Griffiths T, *et al.* The impact of a visual arts program on quality of life, communication, and well-being of people living with dementia: a mixed-methods longitudinal investigation. *Int Psychogeriatrics* 2018;**30**:409–23. doi:10.1017/S1041610217002162

38 Keller HH, Martin LS, Dupuis S, *et al.* Strategies to support engagement and continuity of activity during mealtimes for families living with dementia; a qualitative study. *BMC Geriatr* 2015;**15**:119. doi:10.1186/s12877-015-0120-2

39 Barnes M, Henwood F, Smith N. Information and care: A relational approach. *Dementia* 2016;**15**:510–25. doi:10.1177/1471301214527750

40 Moye J, Marson DC. Assessment of decision-making capacity in older adults: an emerging area of practice and research. *J Gerontol B Psychol Sci Soc Sci* 2007;**62**:P3–11.

41 Karel MJ, Gurrera RJ, Hicken B, *et al.* Reasoning in the capacity to make medical decisions: the consideration of values. *J Clin Ethics* 2010;**21**:58–71.

42 Karlawish J. Measuring Decision-Making Capacity in Cognitively Impaired Individuals. *Neurosignals* 2008;**16**:91–8. doi:10.1159/000109763

43 QSR International Pty Ltd. NVivo qualitative data analysis software. 2018.

## AUTHORS' CONTRIBUTIONS

CDP was involved in the design of the intervention and of the implementation strategy, conceived the evaluation framework, drafted the first version of the protocol and revised it following discussions with the other Authors. JO and MA oversaw the design of the intervention and of the implementation strategy, contributed to developing the evaluation framework and revised the manuscript. CB was involved in the design of the intervention and of the implementation strategy, supported local engagement and revised the manuscript. RG was involved in the design of the intervention and of the implementation strategy, supported local engagement and revised the manuscript. All authors have read and approved the final manuscript.



For peer review only - http://bmjopen.bmj.com/site/about/guidelines.xhtml

**FUNDING STATEMENT**

CDP, JO, RG and MA were supported by The Health Foundation, grant number 1274233. CB received no specific grant from any funding agency in the public, commercial or not-for-profit sectors.

**COMPETING INTERESTS**

None to declare

For peer review only



For peer review only - http://bmjopen.bmj.com/site/about/guidelines.xhtml

**Figure 1 - The phases of the co-creation initiative**

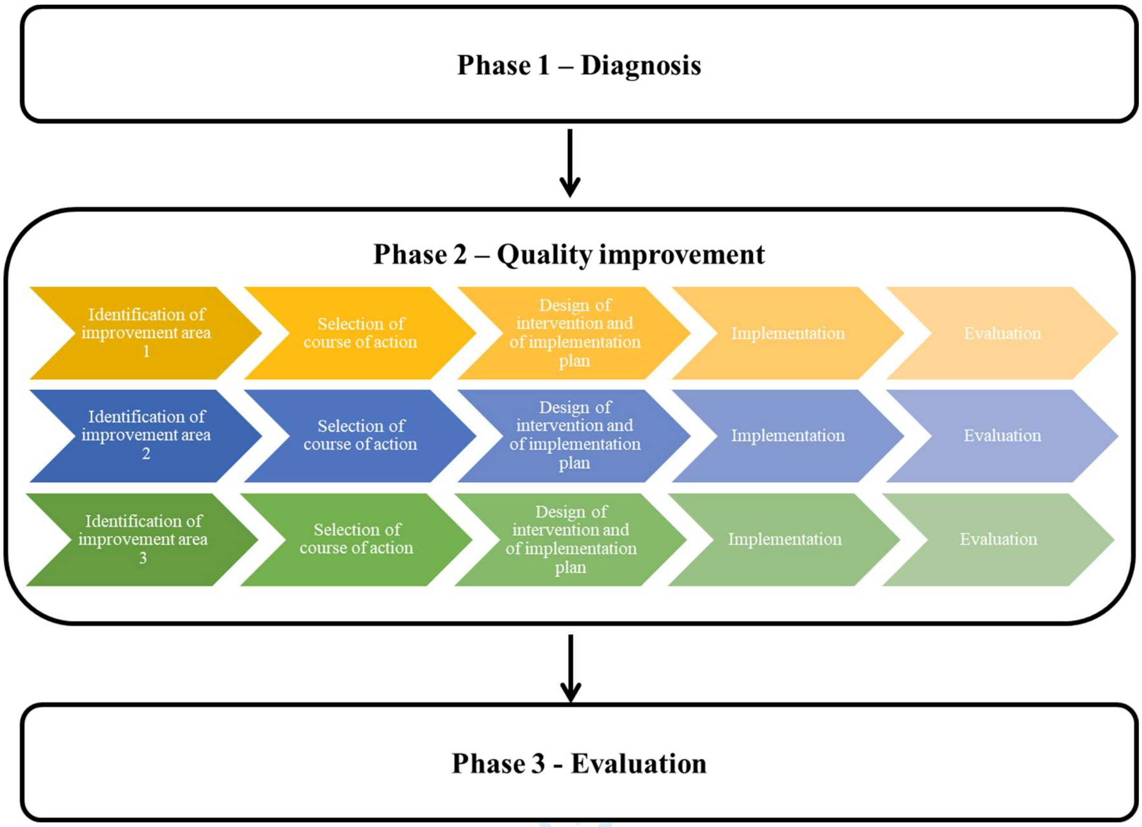

**Figure 2 – Timeline of the development of the intervention**

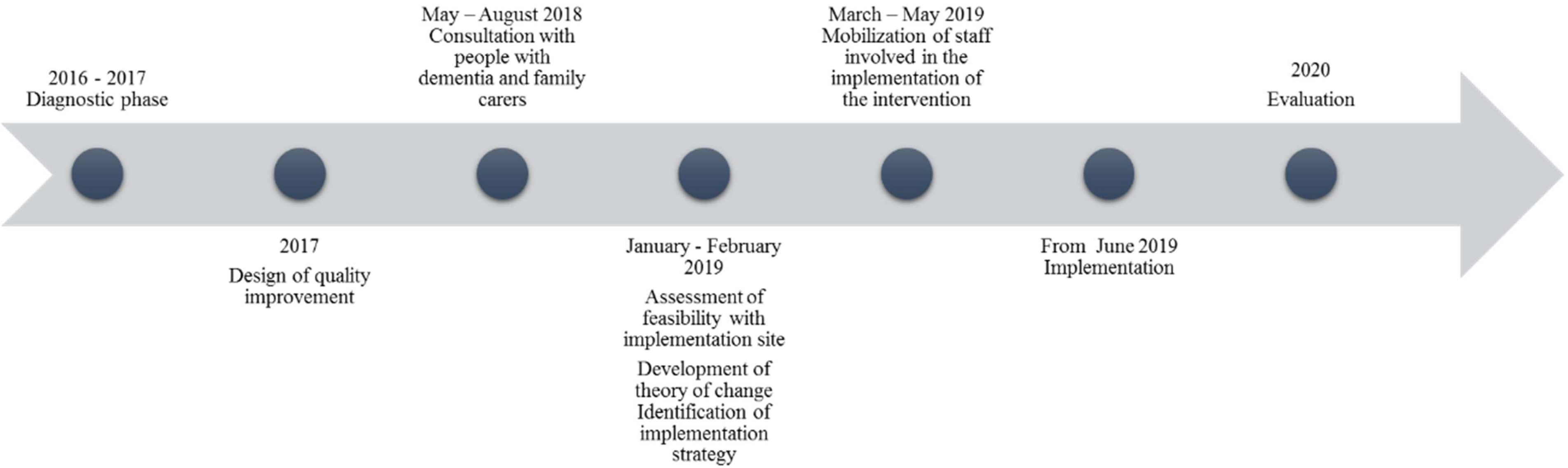

**Figure 3 – The evaluation framework of the intervention**

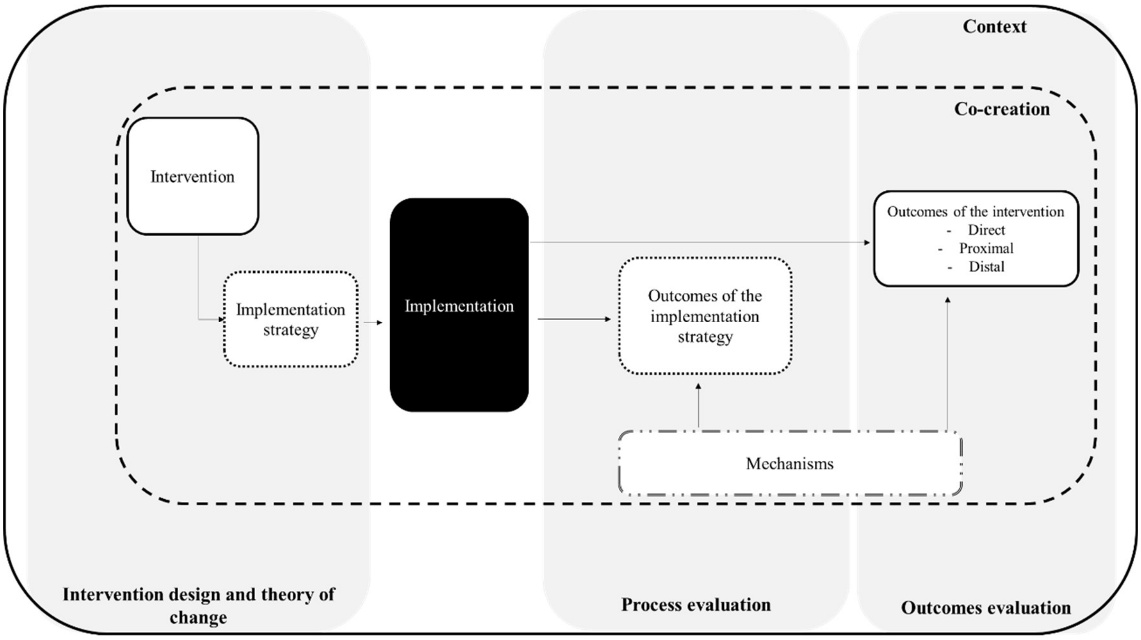

**Figure 4 – Data collection timeline**

| **Month** | | **1** | **2** | **3** | **4** | **5** | **6** | **7** | **8** | **9** | **10** | **11** | **12** |
|---|---|---|---|---|---|---|---|---|---|---|---|---|---|
| **Implementation** | | *Ongoing implementation* | | | | | | | | | | | |
| **People with dementia and family carers** | Recruitment | *Ongoing recruitment* | | | | | | | | | | | |
| | Data collection | | Interview 1 | | | | | | Interview 2 | | | | |
| **Implementers (CPNs, team and service managers)** | Data collection | | | | | | Interviews | | | | | | |
| **Local service providers** | Data collection | Activity log | | | | | | | | | | | |
| | | | | | | | | | | | Interviews | | |
| **Local working group** | Data collection | | | | | | | | | | | | Focus group |

For peer review only

**Supplementary material**

For peer review only

## Interview guide – People living with dementia (interview 1)

| | |
|---|---|
| Preamble | I'd like to ask you to tell me more about your experience of using this leaflet that I believe you recently received… |
| Receiving the leaflet | 1. Do you remember receiving this leaflet? When? Where? From whom? |
| | 2. When you were given the leaflet, did the supplier explained what information were available and how to use it? |
| Information seeking process before the leaflet was offered or alternative to the leaflet | 3. [If not recently diagnosed] – Before you were given the leaflet, how did you go about accessing information on dementia or local support available for yourself? How did you find this process? |
| | 4. Are you aware of similar sources of information? How have you found out about them? |
| | 5. What do you think of these other information materials available? |
| | 6. If you are aware of similar resources, have you used them? If not, why? |
| Expected or initial use | 7. What are your impressions of the new leaflet in terms of its usefulness? |
| | 8. Have you used it already? If yes, when did you use it? If not, do you think you will use it in the future? |
| | 9. If you used it, what particular information were you looking for? Could you find the information you were looking for? |
| | 10. Have you acted upon the information you located – e.g. have you already contacted/accessed a service? |
| | 11. Were there any particular aspects you think may be useful to you over the coming months? |
| Satisfaction | 12. Is there anything in particular you like or do not like about the leaflet? |
| | 13. Are there aspects of the leaflet you might want to change in order to make it better for your purposes? |
| Conclusions | 14. Is there anything else that you'd like to add? |
| Agreeing follow up interview | |
| Thank you | |



For peer review only - http://bmjopen.bmj.com/site/about/guidelines.xhtml

## Interview guide – People living with dementia (follow up)

| | |
|---|---|
| Preamble | I'd like to ask you to tell me more about your experience of using this leaflet that you received some months ago… |
| Use | 1. Since you have been given a copy by your CPN, have you used the leaflet? |
| | 2. If not, why? Do you plan to use it in the future? |
| | 3. If yes, when did you use it? What information were you looking for? Could you find what you were looking for? |
| | 4. Have you acted upon the information you found, e.g. have you contacted/accessed a service? |
| Barriers and facilitators | 5. What helped you to use the leaflet e.g. you received a printed copy or a trusted person gave it to you? |
| | 6. What prevented you from using the leaflet, e.g. format or content? |
| Information seeking process before the leaflet was offered or alternative to the leaflet | 7. Since we last spoke, have you found out about other information resources for people living with dementia? How have you found them? |
| | 8. If you are aware of similar resources, have you used them? If you haven't used them, why not? |
| | 9. What do you think of these other information resources? |
| Impact | 10. Has the leaflet changed the way you look for information about services available locally? If yes, in what way? If not, why? |
| | 11. Has the leaflet changed the care and support you receive? If yes, in what way? If not, why? |
| | 12. Since receiving the leaflet, are you in touch with organizations you previously did not know? |
| Satisfaction | 13. What do you like most/ least about the leaflet? |
| | 14. Is there anything you would change? |
| Future use | 15. Would you expect to receive a new copy of the leaflet if it is updated in the future? How often? From whom? |
| | 16. Would you keep using the leaflet in the future? |
| | 17. Is there anything else that you'd like to add? |
| Thank you | |



For peer review only - http://bmjopen.bmj.com/site/about/guidelines.xhtml

# Interview guide – Family carers (interview 1)

| | |
|---|---|
| Preamble | I'd like to ask you to tell me more about your experience of using this leaflet that I believe you recently received… |
| Receiving the leaflet | 1. Do you remember receiving this leaflet? When? Where? From whom? |
| | 2. When you were given the leaflet, did the supplier explained what information were available and how to use it? |
| Information seeking process before the leaflet was offered or alternative to the leaflet | 3. [If person with dementia not recently diagnosed] – Before you were given the leaflet, how did you go about accessing information on dementia or local support available for yourself or for <name of the person living with dementia>? How did you find this process? |
| | 4. Are you aware of similar sources of information for yourself or for <name of person living with dementia>? How have you found out about them? |
| | 5. What do you think of these other information materials available? |
| | 6. If you are aware of similar resources, have you used them? If not, why? |
| Expected or initial use | 7. What are your impressions of the new leaflet in terms of its usefulness? |
| | 8. Have you used it already? If yes, when did you use it? If not, do you think you will use it in the future? |
| | 9. If you used it, what particular information were you looking for? Could you find the information you were looking for? |
| | 10. Have you acted upon the information you located – e.g. have you already contacted/accessed a service for yourself or for <name of the person living with dementia>? |
| | 11. Were there any particular aspects you think may be useful to you over the coming months? |
| Satisfaction | 12. Is there anything in particular that you like or do not like about the leaflet? |
| | 13. Are there aspects of the leaflet you might want to change in order to make it better for your purposes? |
| Conclusions | 14. Is there anything else that you'd like to add? |
| Agreeing follow up interview | |
| Thank you | |



For peer review only - http://bmjopen.bmj.com/site/about/guidelines.xhtml

## Interview guide – Family carers (follow up)

| | |
|---|---|
| Preamble | I'd like to ask you to tell me more about your experience of using the leaflet that you received some months ago… |
| Use | 1. Since you have been given a copy by your CPN, have you used the leaflet? |
| | 2. If not, why? Do you plan to use it in the future? |
| | 3. If yes, when did you use it? What information were you looking for? Could you find what you were looking for? |
| | 4. Have you acted upon the information you found, e.g. have you contacted/accessed a service for yourself or for <name of the person living with dementia>? |
| Barriers and facilitators | 5. What helped you to use the leaflet e.g. you received a printed copy or a trusted person gave it to you? |
| | 6. What prevented you from using the leaflet, e.g. format or content? |
| Information seeking process before the leaflet was offered or alternative to the leaflet | 7. Since we last spoke, have you found out about other information resources for carers of somebody living with dementia? How have you found them? |
| | 8. If you are aware of similar resources, have you used them? If you haven't used them, why not? |
| | 9. What do you think of these other information resources? |
| Impact | 10. Has the leaflet changed the way you look for information about local services for yourself or for <name of the person living with dementia>? If yes, in what way? If not, why? |
| | 11. Has the leaflet changed the care and support you or <name of the person living with dementia> receive? If yes, in what way? If not, why? |
| | 12. Since receiving the leaflet, are you in touch with organizations you previously did not know? |
| Satisfaction | 13. What do you like most/least about the leaflet? |
| | 14. Is there anything you would change? |
| Future use | 15. Would you expect to receive a new copy of the leaflet if it is updated in the future? How often? From whom? |
| | 16. Would you keep using the leaflet in the future? |
| | 17. Is there anything else that you'd like to add? |
| Thank you | |

For peer review only



For peer review only - http://bmjopen.bmj.com/site/about/guidelines.xhtml

# Interview guide – CPNs from Community Mental Health Services for Older People

| | |
|---|---|
| Preamble | I'd like to ask you about your experience of using the leaflet in clinical practice… |
| Implementation strategy | 1. How were you made aware that the leaflet would have been available to you to use in their clinical work? |
| | 2. Were you given any guidance about when and how to hand the leaflet out to patients? |
| | 3. Were you made aware of the type of patients who should have been given the leaflet? |
| | 4. How was the leaflet made available to you? E.g. was it stored somewhere on your IT system and you had to print it out? |
| | 5. When have you started using it? For how long will you use it in your clinical practice? |
| Actual use | 6. How are you using the leaflet with your patients, e.g. for signposting patients or families to local services? |
| | 7. Once you started using the leaflet in clinical practice, have you changed something in how you are using it? If yes, what have you changed? Why did you change it? |
| | 8. Do you think you reached the expected group(s) of users? |
| | 9. What are the factors that influenced the actual use of the leaflet in your clinical practice? |
| Impact | 10. Has the leaflet changed how your appointments with the patients and families take place? If yes, what has changed? Why has it changed? |
| | 11. Do you think that the leaflet has changed patients' and family carers' perceptions about local care and support available to them? |
| | 12. Do you think that the leaflet has changed how people with dementia and family carers access and use local services? |
| Future use | 13. Do you plan to keep using it? Do you think it should be updated regularly? |
| | 14. Do you think we should change anything about the leaflet (e.g. thinking about its content or its design) or about how it is used in clinical practice? |
| Other strategies | 15. Are you aware of the current strategies or priorities in the area of dementia (e.g. these could be national, local or identified by the organization you work for)? Do you think the leaflet fits with the current strategies or priorities? |
| Co-creation – Process | 16. Are you aware that the leaflet was designed and developed with inputs from both people living with dementia, their family carers, and a wide range of local organizations providing or commissioning care and support for them? If yes, did you contribute to the design and development of the leaflet? How? |

For peer review only

| | |
|---|---|
| Co-creation – Impact | 17. In your opinion did this approach influence the use of the leaflet in clinical practice? In what ways? |
| Conclusion | 18. Do you have any final comment on the leaflet? Or on your experience of using it with your patients and their families? Or on your experience of using it in clinical practice? |
| | 19. Is there anything else that you'd like to add? |
| Thank you | |

For peer review only

## Interview guide - Team and service managers from Community Mental Health Services for Older People

| | |
|---|---|
| Preamble | I'd like to ask you about your experience of using the leaflet in your organization… |
| Implementation strategy | 1. Can you please describe how you organized the dissemination of the leaflet in practice among your staff? How did you inform the staff that the leaflet would have been made available to them to use in their clinical work? How you given your staff any guidance about when and how to hand the leaflet out to patients? |
| | 2. How you identified the type of patients who should have been given the leaflet? Was this made clear to your staff? |
| | 3. How was the leaflet embedded in the clinical practice of CPNs? |
| | 4. How is it made available to them e.g. was it stored somewhere on your IT system and CPNs can print it out? |
| | 5. When have you started using it? For how long have you planned to use it in practice? |
| | 6. Once you started using the leaflet in clinical practice, have you changed how it was originally planned to be used? If yes, what have you changed and why? |
| Actual use | 7. Are you aware of how the CPNs have been using the leaflet with their patients, e.g. for signposting users to local services available? |
| | 8. Do you think you reached the group(s) of users that you were expecting? |
| | 9. Are you aware of any specific factors that influenced the actual use of the leaflet by the CPNs in clinical practice? |
| Barriers and facilitators | 10. In your opinion, what facilitated and hindered the use of the leaflet in clinical practice? |
| Impact of the leaflet | 11. Has the leaflet changed how the appointments of the CPNs with their patients and families take place? Is this what you were expecting from the dissemination of the leaflet? |
| | 12. Do you think that the leaflet has changed patients' and family carers' perceptions about local care and support available? |
| | 13. Do you think that the leaflet has changed how people with dementia and family carers access and use local services? |
| Future use | 14. Do you plan to keep using the leaflet within your organization? Do you think it should be updated regularly? |
| | 15. Do you think we should change anything about the leaflet (e.g. thinking about its content or its design) or about how it is used in clinical practice? |
| Other strategies | 16. Are you aware of the current strategies or priorities in the area of dementia (e.g. these could be national, local or identified by the organization you work for)? Do you think the leaflet fits the current strategies or priorities? |



For peer review only - http://bmjopen.bmj.com/site/about/guidelines.xhtml

| | |
|---|---|
| Co-creation – Process | 17. Are you aware that the leaflet was developed with inputs from people living with dementia, their family carers, and several local organizations providing/commissioning dementia care? Did you contribute to the design and development of the leaflet? How? |
| Co-creation – Impact | 18. In your opinion did this approach influence the use of the leaflet in clinical practice? In what way? |
| Conclusion | 19. Do you have any final comment on the leaflet? Or on your experience of using it with your team/service? |
| | 20. Is there anything else that you'd like to add? |
| Thank you | |

For peer review only



For peer review only - http://bmjopen.bmj.com/site/about/guidelines.xhtml

## Interview guide – Managers from local service providers

| | |
|---|---|
| Preamble | I'd like to review with you the data you have collected and I will need your help to make sense of them… |
| Review the activity log | 1. Can we have a look at the data covering the period in which the leaflet was used? How do these compare with the previous year? |
| Pattern of referrals | 2. Have you seen a change in the referrals that you received since the dissemination of the leaflet, e.g. in terms of<br>- who the referrers are (e.g. self referral or professionals)<br>- for what services the referrals are made<br>- for whom the referrals are made (i.e. person with dementia or family carers)<br>- when a referral is made (e.g. at a crisis point) |
| | 3. Do you think that the leaflet has changed the perceptions of people with dementia and family carers' about local care and support available? |
| | 4. Do you think that the leaflet has changed how people with dementia and family carers access and use local services? |
| Future use | 5. Do you think we should keep offering the leaflet to people with dementia and family carers in the future? |
| | 6. Do you think we should change anything in how/when/to whom the leaflet is offered? |
| Other strategies | 7. Are you aware of current strategies or priorities in the area of dementia (e.g. these could be national, local or identified by the organization you work for)? Do you think the leaflet meets the priorities of current strategies or priorities? |
| Co-creation – Process | 8. Are you aware that the leaflet was developed with input from both people living with dementia, family carers, and a wide range of local organizations providing or commissioning care and support? If yes, did you contribute to the development of the leaflet? How? |
| Co-creation – Impact | 9. In your opinion did this approach influence whether and how people with dementia and family carers used the leaflet? In what ways? |
| Conclusion | 10. Do you have any final comment on the leaflet? Or on your organisation's experience of using it with people with dementia and their families? Or on your organisation's experience of using it in clinical practice? |
| | 11. Is there anything else that you'd like to add? |
| Thank you | |



For peer review only - http://bmjopen.bmj.com/site/about/guidelines.xhtml

**Focus group guide – Leaflet working group**

| | |
|---|---|
| Preamble | I'd like to share with you the feedback about the leaflet that we collected from different perspectives (users, implementers, local providers) and invite you to discuss them … |
| Feedback from people living with dementia and family carers | 1. These are some extracts from interviews carried out with a group of people living with dementia and family carers…. |
| Feedback from implementers (CPNs, team and service managers) | 2. These are some extracts from interviews carried out with a group of CPNs and with the team and service managers of the CMHSOP… |
| Feedback from third sector organizations | 3. These are some data about the trends in referrals into these organizations… |
| | 4. These are some extracts from the interviews carried out with the local providers… |
| Co-creation – Impact | 5. As you are aware, the leaflet was developed collaboratively, with input from different stakeholders. In your opinion did this approach influence whether and how people with dementia and family carers used the leaflet? In what ways? |
| | 6. Similarly, do you think that this approach has had an impact on the use of the leaflet by CPNs? |
| Updating the leaflet | 7. This is the feedback we collected about the leaflet, with some suggestions for improvement… |
| | 8. Do you have any other suggestions for improvement? |
| | 9. Do you have any suggestions for improving the process of dissemination of the leaflet? |
| | 10. Do you intend to update the leaflet and make it available to people living with dementia and family carers? How do you plan to go about this? |
| | 11. Is there anything else you would like to add? |
| Thank you | |

For peer review only

# Research checklist

For peer review only

**Overview of the implementation strategy reported in accordance with the StaRI checklist** [1]

| IMPLEMENTATION STRATEGY (i.e. how the intervention was implemented) | | |
|---|---|---|
| **Item** | **Description and reference to methodological frameworks and reporting standards** | |
| 1. Title | | Effectiveness-implementation hybrid type 2 study evaluating an intervention to support ‘information work’ in dementia care |
| 2. Abstract | | See Abstract of the protocol |
| 3. Introduction | To identify the problem | See Introduction of the protocol |
| 4. Introduction | Rationale for the implementation strategy and the intervention | See Intervention development of the protocol |
| 5. Aims and objectives | | 1. To assess the unfolding of the implementation strategy and its outcomes<br>2. To reflect on the extent to which, how and why the co-creation initiative has influenced the design of the implementation strategy and its unfolding and outcomes |
| 6. The design and key features of the evaluation | | The evaluation of the implementation strategy aims at understanding the feasibility of the intervention by investigating how the actual implementation process has unfolded in practice and identifying any discrepancies between the planned and the actual implementation process |
| 7. Context | Context in which the intervention is implemented | Community mental health services for older people (CMHSOP) within a mental health foundation trust in the North East of England (UK)<br>- North East of England is an under-researched area compared to other parts of England<br>- Local health economy with average recorded diagnosed dementia prevalence (for 2018-2019, local recorded prevalence rate 0.9% vs national recorded prevalence rate 0.8%) and average recorded annual review rate (for 2018-2019, local annual review rate 79.2% vs national recorded prevalence rate 77.9%)[2]<br>- Mental health foundation trust, established in 2006 providing a range of mental health, learning disability and eating disorder services for around two million people across a large and diverse geographical area<br>- CMHSOP provide community-based services for older people experiencing cognitive impairment and mental health problems. Organized around four localities, each locality team includes community psychiatric nurses (CPNs), occupational therapists, physiotherapists, psychologists, support workers to provide multi-disciplinary support<br>Intervention was developed in the context of a 5-year action research co-creation initiative funded by an English charity, involving researchers from three English universities and a research facilitator from the local commissioning support unit alongside a constellation of local stakeholders |

For peer review only - http://bmjopen.bmj.com/site/about/guidelines.xhtml

<table>
<tr><th colspan="3">IMPLEMENTATION STRATEGY (i.e. how the intervention was implemented)</th></tr>
<tr><th>Item</th><th>Description and reference to methodological frameworks and reporting standards</th><th></th></tr>
<tr><td>8. Target sites</td><td></td><td>Four locality teams, staffing levels and caseload are reported in the table below<br><br>
<table>
<tr><th>Team</th><th>Total staff</th><th>CPNs</th><th>Caseload</th></tr>
<tr><td>Team 1</td><td>48</td><td>9</td><td>1,368</td></tr>
<tr><td>Team 2</td><td>43</td><td>8</td><td>1,377</td></tr>
<tr><td>Team 3</td><td>27</td><td>5</td><td>787</td></tr>
<tr><td>Team 4</td><td>26</td><td>5</td><td>791</td></tr>
</table></td></tr>
<tr><td>9. Implementation strategy</td><td>Description of the implementation strategy in accordance with Proctor et al.’s framework for reporting implementation strategies[3]</td><td>Name and definition of the implementation strategy: bundle of professional and material interventions aiming at the development, adoption and use of the dementia leaflet among CPNs (based on framework by [4,5]<br><br>
Specification of the implementation strategy<br>
- Actors: Community Psychiatric Nurses (CPNs) and team managers and service manager across four CMHSOP teams<br>
- Actions: Described in accordance with relevant phase of the Plan-Do-Study-Act (PDSA) framework<br>
  - Plan:<br>
    Professional interventions<br>
    o Established a local working group in charge of overseeing the development the leaflet and its implementation strategy<br>
    o Iteratively developed a dementia leaflet, embedding feedback from people with dementia, family carers, professionals from local organisations providing care and support to people with dementia and family carers<br>
    o Identified the implementation strategy via CPNs based in CMHSOP, identified local champion (service manager), identified implementation sites<br>
    o Organised mobilisation activities with implementation sites, including information sessions at team meetings and email reminders<br>
    Material intervention<br>
    o Printed copies of the leaflet made available to implementers</td></tr>
</table>

For peer review only

| IMPLEMENTATION STRATEGY (i.e. how the intervention was implemented) | | |
|---|---|---|
| **Item** | **Description and reference to methodological frameworks and reporting standards** | |
| | | - Do: CPNs offer a copy of the leaflet to people with dementia or their family carers attending an appointment (any type)<br>- *Action targets*: People living with dementia (any type of dementia, diagnosed at any time) and their family carers attending an appointment with their CPNs. CPNs were expected to use their clinical judgment in identifying patients or family members deemed to be ready to receive the leaflet<br>- *Temporality*: The leaflet can be offered at any point during the period in which the patient was under the care of the CMHSOP (at initial assessment, at post diagnosis, at reviews, at discharge)<br>- *Dose:* Depending on the individual readiness to receive the leaflet and individual and family circumstance, CPNs decide whether to offer or not the leaflet as part of their appointment with the patient. The leaflet could be offered more than once at different points in time |
| 10. Sub-groups | Any subgroups recruited for additional research tasks, and/or nested studies | Not applicable to this study |
| **Evaluation** | | |
| 11. Outcomes | Using Proctor et al.'s taxonomy of outcomes for implementation research [6] | - Acceptability of the intervention by CPNs: perception among CPNs that the intervention is agreeable, palatable, or satisfactory<br>- Adoption of the intervention by CPNs: CPNs' intention, initial decision, or action to try the intervention<br>- Appropriateness of the intervention implemented as judged by CPNs: perceived fit, relevance, or compatibility of the intervention for the setting of CMHSOP and perceived fit of the intervention to address the issue<br>- Cost of the intervention for the implementers<br>- Feasibility of the implementation of the intervention: the extent to which the intervention can be successfully used or carried out within the setting of the CMHSOP<br>- Penetration of the intervention: integration of the intervention within the CMHSOP<br>- Sustainability of the intervention: extent to which the intervention is maintained or institutionalised within the CMHSOP |
| 12. Process evaluation objectives and outcomes related to the mechanism(s) through which the strategy is expected to work | Objectives and outcomes related to the mechanism by which the strategy is expected to work, using MRC framework for complex interventions [7] | - Fidelity of the intervention: adherence to the intervention protocol<br>- Adaptations of the intervention<br>- Dose: the quantity of intervention implemented<br>- Reach: whether the intended audience comes into contact with the intervention, and how |

For peer review only

| IMPLEMENTATION STRATEGY (i.e. how the intervention was implemented) | | |
|---|---|---|
| **Item** | **Description and reference to methodological frameworks and reporting standards** | |
| 13. Data collection methods | | In-depth interviews with CPNs, team managers and service manager of CMHSOP |
| 14. Sample size | | - Up to 12 CPNs from four locality teams<br>- Four team managers<br>- One service manager |
| 15. Methods of analysis | | Thematic content analysis of interview data |

For peer review only

**Overview of the intervention reported in accordance with the StaRI checklist** [1]

| INTERVENTION (i.e. the actual intervention being implemented) | | |
|---|---|---|
| **Item** | **Description and reference to methodological frameworks and reporting standards** | |
| 1. Title | | Effectiveness-implementation hybrid type 2 study evaluating an intervention to support 'information work' in dementia care |
| 2. Abstract | | See Abstract of the protocol |
| 3. Introduction | To identify the problem | See Introduction of the protocol |
| 4. Introduction | Rationale for the implementation strategy and the intervention | See Intervention development of the protocol |
| 5. Aims and objectives | | 1. To assess the outcomes of the intervention as perceived by people living with dementia and their family carers, staff implementing the intervention and local organizations and to explain how and why the intervention contributed to achieve these outcomes<br>2. To reflect on the extent to which, how and why the co-creation initiative has influenced the design, implementation and impact of the intervention |
| 6. The design and key features of the evaluation | | The evaluation of the intervention aims at assessing the outcomes of the intervention with respect to people with dementia and family carers (primary outcomes), to the implementers of the intervention (proximal outcomes) and to local service providers (distal outcomes) |
| 7. Context | Context in which the intervention was implemented | As per implementation strategy described above |
| 8. Target population | Characteristics of the population targeted by the intervention and eligibility criteria | People living with dementia (any type of dementia, diagnosed at any time) and their family carers attending an appointment with their CPNs. CPNs were expected to use their clinical judgment in identifying patients or family members deemed to be ready to receive the leaflet |
| 9. Intervention | Description of the intervention, provided in accordance with the TIDieR checklist [8] | Name: Leaflet about local care and support available to people living with dementia and family carers<br><br>Why: in the diagnostic phase of the co-creation initiative, people living with dementia and family carers consistently reported a lack of timely, up-to-date information provided in a user-friendly format which hinders their 'information work'. The intervention has been designed to provide a short, up-to-date, paper-based source of information that could be handed out and used by CPNs in their encounter with people with dementia and their family carers and support their 'information work' |

For peer review only

| INTERVENTION (i.e. the actual intervention being implemented) | | |
|---|---|---|
| **Item** | **Description and reference to methodological frameworks and reporting standards** | |
| | | What (materials): An A4 three-folded, two-sided leaflet printed in high contrast colour combination. Information are organized in four themes, for which relevant services, a short description and contact details are provided<br><br>What (procedures): CPNs offer the leaflet to patients and their family carers as part of their clinical encounters<br><br>Who: 27 qualified mental health nurses (CPNs) part of the organic cells working across four locality teams of the local CMHSOP who assess, diagnose, review medications and offer post-diagnostic support to people with dementia and signpost family carers to services available to them<br><br>How: the leaflet is offered in the context of the appointment attended by the person with dementia and their family carer. The CPN can offer the leaflet in response to a current specific need of the person with dementia or of the family carer (either explicitly reported or latent) or opportunistically, to make them aware of local services which could be of interest either at the present time or in the future<br><br>Where: the intervention takes place in the outpatient clinics of the CMHSOP services of the local mental health foundation trust<br><br>When and how much: CPNs can offer the leaflet at any point in the period when the patient is under the care of the CMHSOP (e.g. at initial assessment, at diagnosis, in the post diagnostic appointment, at review, upon transfer of care back to GP). It could be offered by the CPNs more than once at different points in time<br><br>Tailoring: CPNs are expected to use their clinical judgement in deciding whether, when, how and to whom to offer the leaflet based on<br>- patients or families' readiness to accept the leaflet (e.g. some patients do not temporarily - or will never - accept a diagnosis of dementia)<br>- length of the appointments (e.g. initial assessment can take up to 90 minutes and patients and families may be overloaded with information)<br>- type of appointments (e.g. titration appointments may cause disappointment in patients) |

For peer review only

| INTERVENTION (i.e. the actual intervention being implemented) | | |
|---|---|---|
| **Item** | **Description and reference to methodological frameworks and reporting standards** | |
| 10. Sub-groups | Any subgroups recruited for additional research tasks, and/or nested studies | Not applicable to this study |
| **Evaluation** | | |
| 11. Outcomes | Primary and other outcomes of the intervention | For people with dementia and family carers (primary outcomes)<br>- their satisfaction with the availability of information on local services (e.g. in terms of timing and quality of information)<br>- their awareness of local services<br>- in the longer term, their access and use of local services<br><br>For implementers of the intervention (proximal outcomes)<br>- CPN, team and service managers' awareness of local services and support available to people diagnosed with dementia and to their family carers<br>- CPN, team and service managers' confidence to hold conversations around using services available to those living with dementia and their family carers<br><br>For local service providers (distal outcomes)<br>- volume of referrals into their services<br>- appropriateness of the use of services among those living with dementia and family carers (e.g. with respect to the type and level of need) |
| 12. Process evaluation objectives and outcomes related to the mechanism(s) through which the strategy is expected to work | Objectives and outcomes related to the mechanism by which the strategy is expected to work, using MRC framework for complex interventions [7] | As per implementation strategy |
| 13. Data collection methods | | People with dementia and family carers<br>- Longitudinal semi-structured in-depth interviews: interview 1 carried out 4-6 weeks after the appointment with the CPN when they receive the leaflet; interview 2 (follow-up) scheduled about 6 months after interview 1<br><br>Implementers of the intervention<br>- Semi-structured in-depth interviews with CPNs, team managers and service manager of CMHSOP (scheduled about 6 months from the start of the implementation of the intervention) |

For peer review only

| INTERVENTION (i.e. the actual intervention being implemented) | | |
|---|---|---|
| **Item** | **Description and reference to methodological frameworks and reporting standards** | |
| | | Local service providers<br>- Activity log consisting of a table recording the monthly number of referrals received by the provider in total, by user group (people with dementia or family carer), by referral source (i.e. self-referral, CMHSOP, social services, other), by reason of the referral (e.g. health and safety assessment, information, support for carer, support for people with dementia), over a 24-month period (i.e. in the 12 months preceding the implementation of the intervention and during the 12-month implementation period)<br>- Semi-structured in-depth interviews with managers (scheduled about 10 months from the start of the implementation of the intervention)<br><br>Local working group<br>- focus group with about 12 participants (including commissioners, statutory service providers, staff from CMHSOP, managers from third sector organizations, a family carer) (scheduled about 12 months from the start of the implementation of the intervention) |
| 14. Sample size | | People living with dementia and family carers<br>- Up to 30 people living with dementia (any type of dementia, diagnosed at any time, willing and able to consent) and family carers (of somebody with any type of dementia, any degree of severity, diagnosed at any time)<br><br>Implementers of the intervention<br>- Up to 12 CPNs from four locality teams<br>- Four team managers<br>- Service manager<br><br>Local service providers<br>- at least three local organisations out of the seven listed on the leaflet as service providers which in preliminary conversations committed to supply data to inform the evaluation<br><br>Local working group<br>- about 12 participants attending the focus group (including commissioners, statutory service providers, staff from CMHSOP, managers from third sector organizations, a person with dementia and a family carer) |

For peer review only

| INTERVENTION (i.e. the actual intervention being implemented) | | |
|---|---|---|
| **Item** | **Description and reference to methodological frameworks and reporting standards** | |
| 15. Methods of analysis | | Thematic content analysis of interview and focus group data<br>Descriptive statistics for referral data provided by local service providers, triangulated with interview data |

For peer review only

## REFERECES


1 Pinnock H, Barwick M, Carpenter CR, *et al.* Standards for Reporting Implementation Studies (StaRI) Statement. *BMJ* 2017;**356**. doi:10.1136/bmj.i6795

2 NHS Digital. QOF 2018/19. https://qof.digital.nhs.uk/ (accessed 6 Mar 2020).

3 Proctor EK, Powell BJ, McMillen JC. Implementation strategies: recommendations for specifying and reporting. *Implement Sci* 2013;**8**:139. doi:10.1186/1748-5908-8-139

4 Mazza D, Bairstow P, Buchan H, *et al.* Refining a taxonomy for guideline implementation: results of an exercise in abstract classification. *Implement Sci* 2013;**8**:32. doi:10.1186/1748-5908-8-32

5 Powell BJ, McMillen JC, Proctor EK, *et al.* A compilation of strategies for implementing clinical innovations in health and mental health. Med. Care Res. Rev. 2012;**69**:123–57. doi:10.1177/1077558711430690

6 Proctor E, Silmere H, Raghavan R, *et al.* Outcomes for implementation research: conceptual distinctions, measurement challenges, and research agenda. *Adm Policy Ment Health* 2011;**38**:65–76. doi:10.1007/s10488-010-0319-7

7 Moore GF, Audrey S, Barker M, *et al.* Process evaluation of complex interventions: Medical Research Council guidance. *BMJ* 2015;**350**:h1258. doi:10.1136/bmj.h1258

8 Hoffmann TC, Glasziou PP, Boutron I, *et al.* Better reporting of interventions: Template for intervention description and replication (TIDieR) checklist and guide. *BMJ* 2014;**348**. doi:10.1136/bmj.g1687


For peer review only



For peer review only - http://bmjopen.bmj.com/site/about/guidelines.xhtml

BMJ Open

# Effectiveness-implementation hybrid type 2 study evaluating an intervention to support 'information work' in dementia care: implementation study protocol

| | |
|---|---|
| Journal: | *BMJ Open* |
| Manuscript ID | bmjopen-2020-038397.R2 |
| Article Type: | Protocol |
| Date Submitted by the Author: | 29-Aug-2020 |
| Complete List of Authors: | De Poli, Chiara; London School of Economics and Political Science, Department of Social Policy and Department of Management<br>Oyebode, Jan; University of Bradford, School of Dementia Studies<br>Binns, Christopher; Tees, Esk and Wear Valleys NHS Trust<br>Glover, Richard; NHS North of England Commissioning Support Unit<br>Airoldi, Mara; University of Oxford, Blavatnik School of Government |
| <b>Primary Subject Heading</b>: | Geriatric medicine |
| Secondary Subject Heading: | Patient-centred medicine, Health services research |
| Keywords: | Dementia < NEUROLOGY, Quality in health care < HEALTH SERVICES ADMINISTRATION & MANAGEMENT, QUALITATIVE RESEARCH |
| | |

SCHOLARONE™
Manuscripts

*I, the Submitting Author has the right to grant and does grant on behalf of all authors of the Work (as defined in the below author licence), an exclusive licence and/or a non-exclusive licence for contributions from authors who are: i) UK Crown employees; ii) where BMJ has agreed a CC-BY licence shall apply, and/or iii) in accordance with the terms applicable for US Federal Government officers or employees acting as part of their official duties; on a worldwide, perpetual, irrevocable, royalty-free basis to BMJ Publishing Group Ltd ("BMJ") its licensees and where the relevant Journal is co-owned by BMJ to the co-owners of the Journal, to publish the Work in this journal and any other BMJ products and to exploit all rights, as set out in our licence.*

*The Submitting Author accepts and understands that any supply made under these terms is made by BMJ to the Submitting Author unless you are acting as an employee on behalf of your employer or a postgraduate student of an affiliated institution which is paying any applicable article publishing charge ("APC") for Open Access articles. Where the Submitting Author wishes to make the Work available on an Open Access basis (and intends to pay the relevant APC), the terms of reuse of such Open Access shall be governed by a Creative Commons licence – details of these licences and which Creative Commons licence will apply to this Work are set out in our licence referred to above.*

*Other than as permitted in any relevant BMJ Author's Self Archiving Policies, I confirm this Work has not been accepted for publication elsewhere, is not being considered for publication elsewhere and does not duplicate material already published. I confirm all authors consent to publication of this Work and authorise the granting of this licence.*

For peer review only

**Effectiveness-implementation hybrid type 2 study evaluating an intervention to support 'information work' in dementia care: implementation study protocol**

Chiara De Poli [a]
Jan Oyebode [b]
Christopher Binns [c]
Richard Glover [d]
Mara Airoldi [e]

[a] Corresponding author. Department of Social Policy and Department of Management, London School of Economics and Political Science, Houghton Street, WC2A 2A London, UK, c.de-poli@lse.ac.uk, phone +44 (0)2079556749

[b] Centre for Applied Dementia Studies, Faculty of Health Studies, University of Bradford, Bradford, UK

[c] Tees, Esk and Wear Valleys NHS Trust, Durham, UK

[d] NHS North of England Commissioning Support, Durham, UK

[e] Blavatnik School of Government, University of Oxford, Oxford, UK

Word count: 4,404



For peer review only - http://bmjopen.bmj.com/site/about/guidelines.xhtml

For peer review only

## ABSTRACT

### Introduction

Patients with long-term conditions consistently report a lack of information around services and support available to them. This unmet need for information is significant among people with dementia and family carers. A quality improvement intervention is being carried out to tackle this issue as part of a co-creation initiative in the North East of England (UK). The intervention consists of the dissemination (via the local community mental health services for older people) of a leaflet about services available to people with dementia and their family carers in the study site. This protocol is reported in accordance with the Standards for Reporting Implementation Studies (StaRI).

### Methods and analysis

This effectiveness-implementation hybrid type 2 study aims at understanding (1) the unfolding and outcomes of the implementation strategy, (2) the outcomes of the intervention (for people with dementia and family carers, staff implementing the intervention, local service providers), (3) the contribution of co-creation to the design and implementation of the intervention and its outcomes. The prospective theory of change of the intervention articulated by local stakeholders is used as a reference framework against which to assess the implementation and outcomes of the intervention. Evaluation data will be collected through in-depth interviews with people with dementia and family carers receiving the intervention, staff implementing the intervention, managers from local service providers. Referral data from local service providers will be collected to triangulate the interview data. A focus group with key stakeholders will support the sense-making of findings. The realist configuration of mechanism – context – outcome, operationalized using an information behaviour model, will inform data analysis and interpretation.

### Ethics and dissemination

Ethical and research governance approvals have been obtained from the West Midlands - South Birmingham Research Ethics Committee. The results of the study will be submitted for publication in peer-reviewed journals and disseminated through conferences.


For peer review only

**Strengths and limitations of this study**

- The study is reported in accordance with the Standards for Reporting Implementation Studies (StaRI) framework complemented by additional relevant reporting standards, such as the Template for intervention description and replication (TIDieR) checklist and Proctor et al.'s framework for specifying and reporting implementation strategies
- The evaluation framework integrates the evaluation of the implementation strategy and of the implemented intervention both in terms of process and of outcomes
- The mixed-method evaluation uses data collected through in-depth interviews with three sets of stakeholders, services' referral data and a focus group
- In-depth, longitudinal interviews with people with dementia are planned to explore their experiences of receiving and using the leaflet, however recruitment can be difficult and dropout rate can be high
- The evaluation will provide insights regarding the process and impact of delivering and receiving the intervention but will have no comparison condition

For peer review only



For peer review only - http://bmjopen.bmj.com/site/about/guidelines.xhtml

## INTRODUCTION

Patient activation, self-management, ageing in place, enablement and shared-decision making are recurrent themes in policy discourses across health systems addressing ageing populations, chronic diseases, multi-morbidity and complex needs.

Underpinning, albeit often implicit, assumptions of such policy discourses is the idea that by providing information, patients and family carers are supported to make better choices, have more agency on managing their own health (or the health of the person they care for), may feel empowered and ultimately may report better outcomes and satisfaction with the care and support they receive.

However, such a view fails to acknowledge the 'information work', i.e. the process of seeking, using, evaluating and sharing of information[1] that is required from patients and family carers in order for them to be able to make better choices or to play a more active role in their own or others' care.[2] Also, it assumes patients and family carers are physically and cognitively able to carry out information work, and it overlooks the emotional processes that influence adaptation to long-term conditions, which may influence whether information work is successfully undertaken.[3]

Extant research both documents and describes the unmet information need of patients and family carers and also offers possible explanations for it.[4–6] This issue has been recognised as highly significant among people with dementia and family carers,[7–11] whose information needs span beyond the topics usually addressed by information materials available, leaving them with some information gaps.[12,13] Moreover, people living with dementia identify a preference to receive information directly from health and social care professionals rather than over the internet, which is the primary source of information to which they are often signposted.[14] They highlight that the timeliness of their information needs, which vary along the dementia trajectory,[15] is seldom taken into account by information providers and resources. They also expect up-to-date information to be provided in a culturally acceptable way and in easy to understand language.[9,16,17] Several user-centred models in the fields of information science have been developed to unpack users' information behaviour and explore how the actual process of information need, seeking and use occurs, in order to explain the observed variations in information need.[18] Notwithstanding the specificities of each, these models consistently acknowledge that information is context-sensitive and uniquely dependent on the individual user, who actively constructs its subjective meaning.[19]

Building on this research, an implementation study is being carried out as part of a co-creation initiative in dementia care taking place in the North East of England (UK). The study aims at designing, implementing and evaluating a leaflet about local services providing care and support to people with dementia and their family carers living in the study site.

This article reports the protocol for the evaluation of the implementation study in accordance with the Standards for Reporting Implementation Studies (StaRI) framework and adopts the recommended dual strands for reporting, one about the intervention being implemented and the other about its implementation strategy (i.e. the 'bundle' of techniques used to enhance the adoption, integration into routine practice and sustainability of the intervention).[20,21]

The evaluation has three aims. First, it intends to review the unfolding and outcomes of the implementation strategy. Secondly, it aims to assess the outcomes of the intervention as perceived by people living with dementia and their family carers, staff implementing the intervention and local organizations providing care and support to people with dementia and family carers and to explain how and why the intervention contributed to achieve these



For peer review only - http://bmjopen.bmj.com/site/about/guidelines.xhtml

outcomes. Thirdly, it will reflect on the extent to which, how and why the co-creation initiative has influenced the design, implementation and impact of the intervention.

## INTERVENTION DEVELOPMENT

The implementation study herein described has been developed as part of a co-creation initiative in dementia care taking place in the North East of England (UK). The initiative, started in 2015 and with expected completion at the end of 2020, involves a constellation of local stakeholders, including people living with dementia and their family carers, health and care professionals, service managers and commissioners alongside a research team, encompassing university-based researchers and a local research facilitator.

Grounded in the principles of action research, co-creation activities were organised in three broad phases (Figure 1).[22] The diagnostic phase (phase 1, 2015-2018) mapped the local dementia care system and identified its strengths and weaknesses. The improvement phase (phase 2, 2017-2018) articulated and designed three interventions to address the priority issues identified in the diagnostic phase. Each improvement area was addressed via a sub-cycle of action research. Local stakeholders with an investment in the respective improvement areas were invited to work with the research team to identify different available options to address the improvement areas, select a course of action and accordingly design an intervention and define its implementation.

The planned evaluation (phase 3, 2020) will focus on both the improvement interventions and the co-creation initiative. With respect to the first, the evaluation aims to assess the outcomes of the implementation strategy and the outcomes of each intervention. With respect to the latter, the evaluation aims to unpack how the co-creation initiative has unfolded in practice and its impact on local policy-making around care and support for people with dementia.

PLEASE INSERT FIGURE 1 ABOUT HERE

### *The intervention and the implementation strategy*

A fundamental unmet need for information was reported by people with dementia and family carers who took part in the diagnostic phase. Local stakeholders agreed to tackle this issue by providing up-to-date, accessible and timely information about local services available to those living with dementia and family carers. Figure 2 provides a timeline of the process underpinning the development of the agreed intervention.

PLEASE INSERT FIGURE 2 ABOUT HERE

A local working group (including commissioners, statutory service providers, third sector organisations providing support and care to people with dementia and family carers, a professional designer, alongside the research team) was set up with the remit of developing an information leaflet and planning its implementation. Efforts to involve family carers and people with dementia by asking local third sector organizations to identify people keen to be involved in the working group resulted in one family carer joining the group. Another turned down the offer because of competing commitments and no-one living with dementia was



For peer review only - http://bmjopen.bmj.com/site/about/guidelines.xhtml

identified. To mitigate their absence, inputs from front line staff providing daily direct support and care to people with dementia and to family carers were used (one was also a family carer). In a similar vein, we relied also on the professional designer who had extensive experience and expertise of designing information material intended to be used by people with cognitive impairment.

The group produced three prototypes which were shared and discussed in a consultation exercise involving about 20 among people with dementia and family carers. The consultation exercise was planned around the schedule of activities or groups organized locally for people with dementia and/or family carers. This solution overcame some of the practical problems that might have limited their participation in the working group.

The preferred prototype was iteratively refined using best practice guidance on Patient Information Leaflets[23] and taking into account feedback provided by its potential users, commissioners and service providers. Following local discussion, it was agreed to design only one version of the leaflet for both family caregivers and individuals living with dementia. The leaflet was intended to cover essential information about, and contact details of, the key local organizations that provide dementia-related support and services, which, in practice, often cater for both groups. The intention was to signpost people to such organizations, rather than providing an exhaustive list of services offered. Hence, two versions of the leaflet would not have added substantially different content but could have contributed to information overload, sometimes experienced by those living with dementia and their families (for example around the time of receiving the diagnosis). In terms of graphics, the design aimed to maximize accessibility, irrespective of the intended user's group. From the implementation perspective, it was felt that it would be easier to embed one version of the leaflet in the practice of professionals and, equally, designing and using one version of the leaflet could, more likely, contribute to its sustainability in the longer term, for example by simplifying the updating process.

In its final version, the leaflet presents services clustered around four main themes ("I want to speak to someone for advice and support", "I want to live safely and with the right support", "I want to know what to do in a crisis", "Where can I go for more information?") and it can be printed out on a two-sided A4 using a high contrast colour combination.

Subsequently, the working group agreed a phased implementation process: the Community Mental Health Services for Older People (CMHSOP) within the local mental health trust were identified as the first implementation route because of their key role in diagnosing dementia and in the resulting post-diagnostic care planning. Following diagnosis and initial care planning, those whose needs are stable are discharged to primary care while those with more complex needs continue to receive secondary services.

The working group preliminarily explored with the CMHSOP service manager, team managers and representatives of the Community Psychiatric Nurses (CPNs) how the leaflet could be used in clinical practice. In January 2019, following positive and supportive feedback, the leaflet working group members and the CMHSOP service manager, team managers and two CPNs took part in a focus group aimed at articulating the theory of change associated with the intervention itself. This exercise helped making explicit the expected process of implementing the intervention in practice and its anticipated impact. Mobilisation activities (e.g. information sessions within team meetings and regular reminders emailed to



For peer review only - http://bmjopen.bmj.com/site/about/guidelines.xhtml

team managers and cascaded to CPNs) were planned and carried out to inform, and subsequently to remind, all CPNs about the leaflet and its use in practice. In June 2019, all the CPNs across the four locality teams covering the study site were invited to start using the leaflet in their clinical practice.

CPNs agreed to hand out the leaflet to patients on their caseload who received a diagnosis of any type of dementia and/or to their family carers. CPNs were expected to use their clinical judgement in deciding to whom and when to offer the leaflet, dependent upon the patient's readiness to receive the leaflet and their individual and family circumstances. They expected to hand it out during their encounter with patients and families, when they felt that the leaflet would support their conversations with them. For these reasons, the use of the leaflet was not embedded at a specific point of the clinical pathway.

A detailed description of the intervention and its implementation strategy are outlined in accordance with the StaRI framework respectively in Table 1 and Table 2 (item 9).

For peer review only



For peer review only - http://bmjopen.bmj.com/site/about/guidelines.xhtml

**Table 1 – Overview of the implementation strategy reported in accordance with the StaRI checklist**

| IMPLEMENTATION STRATEGY (i.e. how the intervention was implemented) | | |
|---|---|---|
| **Item** | **Description and reference to methodological frameworks and reporting standards** | |
| 1. Title | | Effectiveness-implementation hybrid type 2 study evaluating an intervention to support 'information work' in dementia care |
| 2. Abstract | | See Abstract of the protocol |
| 3. Introduction | To identify the problem | See Introduction of the protocol |
| 4. Introduction | Rationale for the implementation strategy and the intervention | See Intervention development of the protocol |
| 5. Aims and objectives | | 1. To assess the unfolding of the implementation strategy and its outcomes<br>2. To reflect on the extent to which, how and why the co-creation initiative has influenced the design of the implementation strategy and its unfolding and outcomes |
| 6. The design and key features of the evaluation | | The evaluation of the implementation strategy aims at understanding the feasibility of the intervention by investigating how the actual implementation process has unfolded in practice and identifying any discrepancies between the planned and the actual implementation process |
| 7. Context | Context in which the intervention is implemented | Community mental health services for older people (CMHSOP) within a mental health foundation trust in the North East of England (UK)<br>- North East of England is an under-researched area compared to other parts of England<br>- Local health economy with average recorded diagnosed dementia prevalence (for 2018-2019, local recorded prevalence rate 0.9% vs national recorded prevalence rate 0.8%) and average recorded annual review rate (for 2018-2019, local annual review rate 79.2% vs national recorded prevalence rate 77.9%)[24]<br>- Mental health foundation trust, established in 2006 providing a range of mental health, learning disability and eating disorder services for around two million people across a large and diverse geographical area<br>- CMHSOP providing community-based services for older people experiencing cognitive impairment and mental health problems. Organized around four localities, each locality team includes community psychiatric nurses (CPNs), occupational therapists, physiotherapists, psychologists, support workers to provide multi-disciplinary support<br>Intervention was developed in the context of a 5-year action research co-creation initiative funded by an English charity, involving researchers from three English universities and a |


For peer review only - http://bmjopen.bmj.com/site/about/guidelines.xhtml

| IMPLEMENTATION STRATEGY (i.e. how the intervention was implemented) | | |
|---|---|---|
| **Item** | **Description and reference to methodological frameworks and reporting standards** | |
| | | research facilitator from the local commissioning support unit alongside a constellation of local stakeholders |
| 8. Target sites | | Four locality teams, staffing levels and caseload are reported in the table below<br><br>**Team** / **Total staff** / **CPNs** / **Caseload**<br>Team 1 / 48 / 9 / 1,368<br>Team 2 / 43 / 8 / 1,377<br>Team 3 / 27 / 5 / 787<br>Team 4 / 26 / 5 / 791 |
| 9. Implementation strategy | Description of the implementation strategy in accordance with Proctor et al.'s framework for reporting implementation strategies[25] | **Name and definition of the implementation strategy**: bundle of professional and material interventions aiming at the development, adoption and use of the dementia leaflet among CPNs (based on framework by [26,27]<br><br>**Specification of the implementation strategy**<br>- *Actors*: Community Psychiatric Nurses (CPNs),team managers and service manager across four CMHSOP teams<br>- *Actions*: Described in accordance with relevant phase of the Plan-Do-Study-Act (PDSA) framework<br>  - Plan:<br>    Professional interventions<br>    o Established a local working group in charge of overseeing the development the leaflet and its implementation strategy<br>    o Iteratively developed a dementia leaflet, embedding feedback from people with dementia, family carers, professionals from local organisations providing care and support to people with dementia and family carers<br>    o Identified the implementation strategy via CPNs based in CMHSOP, identified local champion (service manager), identified implementation sites<br>    o Organised mobilisation activities with implementation sites, including information sessions at team meetings and email reminders<br>    Material intervention |



For peer review only - http://bmjopen.bmj.com/site/about/guidelines.xhtml

| IMPLEMENTATION STRATEGY (i.e. how the intervention was implemented) | | |
|---|---|---|
| **Item** | **Description and reference to methodological frameworks and reporting standards** | |
| | | ○ Printed copies of the leaflet made available to implementers<br>- Do: CPNs offer a copy of the leaflet to people with dementia or their family carers attending an appointment (any type)<br>- *Action targets*: People living with dementia (any type of dementia, diagnosed at any time) and their family carers attending an appointment with their CPNs. CPNs were expected to use their clinical judgment in identifying patients or family members deemed to be ready to receive the leaflet<br>- *Temporality*: The leaflet can be offered at any point during the period in which the patient was under the care of the CMHSOP (at initial assessment, at post diagnosis, at reviews, at discharge)<br>- *Dose:* Depending on the individual readiness to receive the leaflet and individual and family circumstance, CPNs decide whether to offer or not the leaflet as part of their appointment with the patient. The leaflet could be offered more than once at different points in time |
| 10. Sub-groups | Any subgroups recruited for additional research tasks, and/or nested studies | Not applicable to this study |
| **Evaluation** | | |
| 11. Outcomes | Using Proctor et al.'s taxonomy of outcomes for implementation research [28] | - Acceptability of the intervention by CPNs: perception among CPNs that the intervention is agreeable, palatable, or satisfactory<br>- Adoption of the intervention by CPNs: CPNs' intention, initial decision, or action to try the intervention<br>- Appropriateness of the intervention implemented as judged by CPNs: perceived fit, relevance, or compatibility of the intervention for the setting of CMHSOP and perceived fit of the intervention to address the issue<br>- Cost of the intervention for the implementers<br>- Feasibility of the implementation of the intervention: the extent to which the intervention can be successfully used or carried out within the setting of the CMHSOP<br>- Penetration of the intervention: integration of the intervention within the CMHSOP<br>- Sustainability of the intervention: extent to which the intervention is maintained or institutionalised within the CMHSOP |
| 12. Process evaluation objectives and outcomes related to the mechanism(s) through which the strategy is expected to work | Objectives and outcomes related to the mechanism by which the strategy is expected to work, using MRC | - Fidelity of the intervention: adherence to the intervention protocol<br>- Adaptations of the intervention<br>- Dose: the quantity of intervention implemented |



For peer review only - http://bmjopen.bmj.com/site/about/guidelines.xhtml

| IMPLEMENTATION STRATEGY (i.e. how the intervention was implemented) | | |
|---|---|---|
| **Item** | **Description and reference to methodological frameworks and reporting standards** | |
| | framework for complex interventions [29] | - Reach: whether the intended audience comes into contact with the intervention, and how |
| 13. Data collection methods | | In-depth interviews with CPNs, team managers and service manager of CMHSOP |
| 14. Sample size | | - Up to 12 CPNs from four locality teams<br>- Four team managers<br>- One service manager |
| 15. Methods of analysis | | Thematic content analysis of interview data |

For peer review only

**Table 2 – Overview of the intervention reported in accordance with the StaRI checklist**

| **INTERVENTION (i.e. the actual intervention being implemented)** | | |
|---|---|---|
| **Item** | **Description and reference to methodological frameworks and reporting standards** | |
| 1. Title | | Effectiveness-implementation hybrid type 2 study evaluating an intervention to support 'information work' in dementia care |
| 2. Abstract | | See Abstract of the protocol |
| 3. Introduction | To identify the problem | See Introduction of the protocol |
| 4. Introduction | Rationale for the implementation strategy and the intervention | See Intervention development of the protocol |
| 5. Aims and objectives | | 1. To assess the outcomes of the intervention as perceived by people living with dementia and their family carers, staff implementing the intervention and local organizations and to explain how and why the intervention contributed to achieve these outcomes<br>2. To reflect on the extent to which, how and why the co-creation initiative has influenced the design, implementation and impact of the intervention |
| 6. The design and key features of the evaluation | | The evaluation of the intervention aims at assessing the outcomes of the intervention with respect to people with dementia and family carers (primary outcomes), to the implementers of the intervention (proximal outcomes) and to local service providers (distal outcomes) |
| 7. Context | Context in which the intervention was implemented | As per implementation strategy described above |
| 8. Target population | Characteristics of the population targeted by the intervention and eligibility criteria | People living with dementia (any type of dementia, diagnosed at any time) and their family carers attending an appointment with their CPNs. CPNs were expected to use their clinical judgment in identifying patients or family members deemed to be ready to receive the leaflet |
| 9. Intervention | Description of the intervention, provided in accordance with the TIDieR checklist [30] | Name: Leaflet about local care and support available to people living with dementia and family carers<br><br>Why: in the diagnostic phase of the co-creation initiative, people living with dementia and family carers consistently reported a lack of timely, up-to-date information provided in a user-friendly format, which hinders their 'information work'. The intervention has been designed to provide a short, up-to-date, paper-based source of information that could be handed out and used by CPNs in their encounter with people with dementia and their family carers and support their 'information work' |



For peer review only - http://bmjopen.bmj.com/site/about/guidelines.xhtml

| INTERVENTION (i.e. the actual intervention being implemented) | | |
|---|---|---|
| **Item** | **Description and reference to methodological frameworks and reporting standards** | |
| | | What (materials): An A4 three-folded, two-sided leaflet printed in high contrast colour combination. Information are organized in four themes, for which relevant services, a short description and contact details are provided<br><br>What (procedures): CPNs offer the leaflet to patients and their family carers as part of their clinical encounters<br><br>Who: 27 qualified mental health nurses (CPNs) part of the organic cells working across four locality teams of the local CMHSOP who assess, diagnose, review medications and offer post-diagnostic support to people with dementia and signpost family carers to services available to them<br><br>How: the leaflet is offered in the context of the appointment attended by the person with dementia and their family carer. The CPN can offer the leaflet in response to a current specific need of the person with dementia or of the family carer (either explicitly reported or latent) or opportunistically, to make them aware of local services which could be of interest either at the present time or in the future<br><br>Where: the intervention takes place in the outpatient clinics of the CMHSOP services of the local mental health foundation trust<br><br>When and how much: CPNs can offer the leaflet at any point in the period when the patient is under the care of the CMHSOP (e.g. at initial assessment, at diagnosis, in the post diagnostic appointment, at review, upon transfer of care back to GP). It could be offered by the CPNs more than once at different points in time<br><br>Tailoring: CPNs are expected to use their clinical judgement in deciding whether, when, how and to whom to offer the leaflet based on<br>- patients or families' readiness to accept the leaflet (e.g. some patients do not temporarily - or will never - accept a diagnosis of dementia)<br>- length of the appointments (e.g. initial assessment can take up to 90 minutes and patients and families may be overloaded with information)<br>- type of appointments (e.g. titration appointments may cause disappointment in patients) |

For peer review only

| INTERVENTION (i.e. the actual intervention being implemented) | | |
|---|---|---|
| **Item** | **Description and reference to methodological frameworks and reporting standards** | |
| 10. Sub-groups | Any subgroups recruited for additional research tasks, and/or nested studies | Not applicable to this study |
| **Evaluation** | | |
| 11. Outcomes | Primary and other outcomes of the intervention | For people with dementia and family carers (primary outcomes)<br>- their satisfaction with the availability of information on local services (e.g. in terms of timing and quality of information)<br>- their awareness of local services<br>- in the longer term, their access and use of local services<br><br>For implementers of the intervention (proximal outcomes)<br>- CPN, team and service managers' awareness of local services and support available to people diagnosed with dementia and to their family carers<br>- CPN, team and service managers' confidence to hold conversations around using services available to those living with dementia and their family carers<br><br>For local service providers (distal outcomes)<br>- volume of referrals into their services<br>- appropriateness of the use of services among those living with dementia and family carers (e.g. with respect to the type and level of need) |
| 12. Process evaluation objectives and outcomes related to the mechanism(s) through which the strategy is expected to work | Objectives and outcomes related to the mechanism by which the strategy is expected to work, using MRC framework for complex interventions [29] | As per implementation strategy |
| 13. Data collection methods | | People with dementia and family carers<br>- Longitudinal semi-structured in-depth interviews: interview 1 carried out 4-6 weeks after the appointment with the CPN when they receive the leaflet; interview 2 (follow-up) scheduled about 6 months after interview 1<br><br>Implementers of the intervention<br>- Semi-structured in-depth interviews with CPNs, team managers and service manager of CMHSOP (scheduled about 6 months from the start of the implementation of the intervention) |



For peer review only - http://bmjopen.bmj.com/site/about/guidelines.xhtml

| INTERVENTION (i.e. the actual intervention being implemented) | | |
|---|---|---|
| **Item** | **Description and reference to methodological frameworks and reporting standards** | |
| | | Local service providers<br>- Activity log consisting of a table recording the monthly number of referrals received by the provider in total, by user group (people with dementia or family carer), by referral source (i.e. self-referral, CMHSOP, social services, other), by reason of the referral (e.g. health and safety assessment, information, support for carer, support for people with dementia), over a 24-month period (i.e. in the 12 months preceding the implementation of the intervention and during the 12-month implementation period)<br>- Semi-structured in-depth interviews with managers (scheduled about 10 months from the start of the implementation of the intervention)<br><br>Local working group<br>- focus group with about 12 participants (including commissioners, statutory service providers, staff from CMHSOP, managers from third sector organizations, a family carer) (scheduled about 12 months from the start of the implementation of the intervention) |
| 14. Sample size | | People living with dementia and family carers<br>- Up to 30 people living with dementia (any type of dementia, diagnosed at any time, willing and able to consent) and family carers (of somebody with any type of dementia, any degree of severity, diagnosed at any time)<br><br>Implementers of the intervention<br>- Up to 12 CPNs from four locality teams<br>- Four team managers<br>- Service manager<br><br>Local service providers<br>- at least three local organisations out of the seven listed on the leaflet as service providers which in preliminary conversations committed to supply data to inform the evaluation<br><br>Local working group<br>- about 12 participants attending the focus group (including commissioners, statutory service providers, staff from CMHSOP, managers from third sector organizations, a person with dementia and a family carer) |



For peer review only - http://bmjopen.bmj.com/site/about/guidelines.xhtml

| INTERVENTION (i.e. the actual intervention being implemented) | | |
|---|---|---|
| **Item** | **Description and reference to methodological frameworks and reporting standards** | |
| 15. Methods of analysis | | Thematic content analysis of interview and focus group data<br>Descriptive statistics for referral data provided by local service providers, triangulated with interview data |

For peer review only

## METHODS AND ANALYSIS

### *Evaluation framework*

The study adopts an effectiveness-implementation hybrid type 2 design which allows simultaneous evaluation of the intervention and its implementation strategy,[31] as reflected in the evaluation framework (Figure 3).

PLEASE INSERT FIGURE 3 ABOUT HERE

The evaluation framework maps the phases of the study (design – implementation – evaluation) on to the key components of the intervention and shows the relations among them. In the design phase, the intervention and the implementation strategy (i.e. the bundle of professional and material interventions intended to sustain the adoption and use of the leaflet among CPNs) were articulated with the theory of change focus group. Following the implementation, the evaluation will

1. assess the unfolding of the implementation strategy and its outcomes
2. assess the outcomes of the intervention as perceived by people with dementia and family carers, the implementers of the intervention, as well as the providers of local services available to people with dementia and their families
3. identify the mechanisms of action, i.e. the "active ingredients" that ignited the implementation strategy and the intervention and, hence, contributed to the observed outcomes
4. reflect on the contribution of context (e.g. the social, economic, policy and organisational factors surrounding the intervention, including, but not limited to, the co-creation initiative, as described in Table 1, item 7) to the design and implementation of the intervention and its implementation strategy and relative outcomes

Outcomes of the implementation strategy

The implementation strategy is evaluated with respect to both its outcomes and its process dimension, as per the StaRI framework. Following Proctor et al.'s taxonomy[28], the following outcomes will be assessed with respect to the implementation strategy (Table 1, item 11):

- Acceptability of the intervention by CPNs: perception among CPNs that the intervention is agreeable, palatable, or satisfactory
- Adoption of the intervention by CPNs: CPNs' intention, initial decision, or action of trying out the intervention
- Appropriateness of the intervention implemented as judged by CPNs: perceived fit, relevance, or compatibility of the intervention for the setting of the CMHSOP and perceived fit of the intervention to address the issue
- Cost of the intervention for the implementers
- Feasibility of the implementation of the intervention: the extent to which the intervention can be successfully used or carried out within the setting of the CMHSOP



For peer review only - http://bmjopen.bmj.com/site/about/guidelines.xhtml

- Penetration of the intervention: the extent to which the intervention is integrated into the practice of the CMHSOP
- Sustainability of the intervention: extent to which the intervention is maintained or embedded within the CMHSOP

Outcomes of the intervention

The theory of change exercise run in January 2019 highlighted that the intervention was expected to have an impact on (Table 2, item 11):

- Its main target population, i.e. people with dementia and family carers (direct outcomes)
- The implementers of the intervention, i.e. CPNs, team and service managers of the CMHSOP delivering the intervention (proximal outcomes)
- Local providers of care and support to people with dementia and their families, such as local third sector organisations (distal outcomes)

With respect to people with dementia and family carers, the intervention was expected to improve three outcomes directly associated with the intervention: (i) their satisfaction with the availability of information on local services (e.g. in terms of timing and quality of information), (ii) their awareness of local services and (iii) in the longer term, their access and use of local services.

With respect to the implementers, the intervention was expected to influence two proximal outcomes : (i) CPN, team and service manager's awareness of local services and support available to people diagnosed with dementia and to their family carers and (ii) CPN, team and service manager's confidence to hold conversations with people living with dementia and their family carers about seeking support and using local services.

With respect to local service providers, the intervention was expected to impact on two distal outcomes: (i) volume of referrals into their services and (ii) appropriateness of the use of services among those living with dementia and family carers (e.g. with respect to the type and level of need).

Process evaluation

The MRC framework for process evaluation[29] is used to investigate (Table 1 and Table 2, item 12)

- the actual implementation strategy, in terms of implementation fidelity (or, conversely, adaptation), reach and dose,
- the mechanisms of action of the implementation strategy and of the intervention which brought about change

The process evaluation will highlight how the implementation process has unfolded in practice and any discrepancies between the actual implementation process and how it was articulated in the theory of change. Moreover, it will help to surface how and why the intervention and its implementation strategy have contributed to achieve these outcomes.



For peer review only - http://bmjopen.bmj.com/site/about/guidelines.xhtml

### *Analytical framework*

The framework underpinning the realist evaluation will be adopted as analytical tool. Realist evaluation acknowledges that interventions work (have successful outcomes) in so far as they introduce appropriate ideas and opportunities (mechanisms) to groups of stakeholders in the appropriate social and cultural conditions (context), as condensed in the following formula [32]

$$\text{mechanism} + \text{context} = \text{outcome}$$

According to the realist framework, mechanisms are the underlying processes or hidden causal levers of intervention activities that make them work[33] and are represented by the psychological or social explanations of behaviour of intervention participants and their ideas and reasoning about how change is achieved.[34,35] Context refers to the conditions likely to enable or constrain the activation of interventions' mechanisms (e.g. the organisational setting or the broader policy and institutional environment in which an intervention is embedded). Outcome is the anticipated and unanticipated consequence that is brought about by the interaction of different interventions' mechanisms in different contexts.

The realist framework will be overlaid with Dervin's sense-making theory, grounded in the user-centred paradigm in information science research, which allows assessment of "how people make sense of their worlds and how they use information and other resources in the process".[36] The theory assumes that in the complexity of the real world in which people constantly face 'discontinuous' situations, breaks in an individual's knowledge exist, signalling information need, and may provoke the seeking and use of information to help create new knowledge ('sense').[19,36,37] The sense-making process is operationalized in terms of four elements: the *situation*, i.e. the time-space context in which the information needs arise and sense is constructed; the *gap*, i.e. a cognitive barrier that could be caused by the lack of relevant information or the uncertainty of a situation; the *bridge*, i.e. the strategy supporting the information seeking process that helps to close the gap; the *use*, i.e. the outcome of the information seeking process in terms of how the information obtained or constructed is used and what sense the information enables users to make.[18,19,36]

Dervin's concepts of situation, gap/bridge and use will be adopted to operationalize the realist concept of context, mechanisms and outcomes in the analytical framework.

### *Data collection methods and samples*

The mixed-method evaluation will build on data collected from four sets of stakeholders involved in the intervention (Table 1 and Table 2, items 13 and 14):

- people living with dementia and family carers who are at the immediate receiving end of the intervention
- CPNs, team and service managers based in the CMHSOP who are implementing the intervention



For peer review only - http://bmjopen.bmj.com/site/about/guidelines.xhtml

- local providers of care and support to people with dementia and their families, as the organisations expected to observe a change in their service access and use as a consequence of the intervention
- local working group, who contributed to the development of the intervention

Figure 4 provides an overview of the data collection timeline.

PLEASE INSERT FIGURE 4 ABOUT HERE

For peer review only

People with dementia and family carers

Semi-structured in-depth interviews with up to 30 people living with dementia (any type of dementia, diagnosed at any time, willing and able to consent) and family carers (of somebody with any type of dementia, any degree of severity, diagnosed at any time) will take place at two points in time: interview 1 will be carried out 4-6 weeks after the appointment with the CPN when they receive the leaflet and interview 2 (follow up) will be scheduled about 6 months after interview 1.

The two timepoints have been established to best capture participants' experiences of receiving (interview 1) and using (follow up interview) the leaflet (the interview guides are provided as Supplementary file). Accordingly, interview 1 will aim to understand (i) if, when and how they received the leaflet; (ii) whether and how they anticipate using the leaflet; (iii) what they think of the leaflet overall (e.g. whether it has changed their perceptions and awareness of the local services offering care and support to those living with dementia and their family carers). Interview 2 will aim to understand (i) whether, how, when and why they used (or not) the leaflet; (ii) whether and how the leaflet has changed their care seeking behaviour; (iii) their satisfaction with the leaflet.

Longitudinal interviews with people living with dementia and their family carers are a well-established research method.[38–42] The follow up period is appropriate for research purposes. Over a period of 6 months, personal and family circumstances can change and this may prompt those living with dementia and their family carers to seek help and support. Similarly, those recently diagnosed may not feel immediately ready to get in touch with local services but this could change after 6 months.

This purposeful sample will be recruited when they attend their first appointment with the CPN. The size of the sample is expected to allow to capture a wide range of perspectives (varying for roles, i.e. people with dementia vs family carers, types of dementia diagnosis, levels of cognitive impairment, age, gender and living arrangements) while achieving data saturation. The recruitment will be carried out on a rolling basis alongside the implementation of the intervention and will stop when the desired sample size is reached.

Community Mental Health Services for Older People (CMHSOP)

In-depth interviews with CPNs, team and service managers from the implementing CMHSOP will be carried out about 6 months from the start of dissemination of the leaflet. Up to 20 staff members will be recruited across the 4 localities in which the CMHSOP operate.



For peer review only - http://bmjopen.bmj.com/site/about/guidelines.xhtml

The interviews aim to understand (i) whether, when and how they used (or not) the leaflet in clinical practice; (ii) whether and how the leaflet has changed any aspect of their clinical practice; (iii) their views about the impact of adopting the principles of co-creation on the implementation and impact of the intervention. The interview guides are provided as Supplementary file.

Local service providers

Referral data from a minimum of three out of the seven local organisations listed on the leaflet which provide care and support to people living with dementia and their family carers will be collected for a period of 24 months (in the 12 months preceding the implementation and for the 12-month implementation period). Semi-structured in-depth interviews with service managers will be undertaken about 10 months from the start of the dissemination of the leaflet to (i) review the referral data and (ii) identify and interpret the patterns of referral into each service (e.g. over time, with respect to the referral source or the user group) and their appropriateness (e.g. with respect to the type and level of need). The interview guide is provided as Supplementary file.

Local working group

A focus group with the local working group (about 12 participants including commissioners, statutory service providers, staff from CMHSOP, managers from third sector organizations, a family carer) will be held towards the end of the implementation period, following preliminary analysis of the evaluation data (see the Supplementary file for the focus group guide). This will aim to

- discuss and validate the emerging findings about the evaluation of the implementation strategy
- discuss and validate the emerging findings around the impact of the intervention on people with dementia and their families, CPNs and mental health services and local service providers
- reflect on the impact of co-creation initiative on the implementation and impact of the intervention

***Informed consent***

Written and verbal versions of the Participant Information Sheet and Informed Consent Sheet will be presented to participants. The Participant Information Sheet details the nature of the study, what participation will involve, any risks involved. It will be clearly stated that the participant is free to withdraw at any time, for any reason without impact on future care or service access, and with no obligation to give the reason for withdrawal. Participants will be allowed as much time as they wish to consider the information (at least 48 hours), and will have the opportunity to ask questions to the Investigator, their GP and/or CPNs (for people with dementia and family carers), or other independent parties in order to decide whether they will participate in the study. Written informed consent will be obtained.

Maximum efforts will be devoted to recruit people living with dementia into this study. Historically, people with dementia have been excluded from much research in social and



For peer review only - http://bmjopen.bmj.com/site/about/guidelines.xhtml

health care on the basis that their declining cognitive abilities may affect capacity to consent.[43] However, the ability of many people with dementia to provide informed consent, based on their ability to understand information, evaluate risks and benefits, and communicate decisions effectively has been established.[44,45] Prior to the interview, an informal conversation about the research will allow the researchers to ascertain that the potential participant has capacity to consent. If the researcher believes that the person with dementia has capacity, she will take written informed consent and will start the interview.

### *Data analysis*

The referral data provided by each local provider will be collated, tabulated and described with simple metrics (e.g. number of referrals in total, by user group, by referral source, by reason for referral). The results will be triangulated with interview data collected from the participants living with dementia and family carers, the implementers and the managers of the local service providers.

The interviews will be transcribed, anonymised and analysed using thematic analysis (performed with NVivo12) (Table 1 and Table 2, item 15).[46] Following the realist approach, the initial codebook will be developed around the themes (codes) of context conditions, underlying mechanisms, observed outcomes matched on to implementation strategy and intervention. These will be then refined into sub-codes to capture specificities of data collected from different interviewee roles or to highlight different dimensions featuring in a main code. For example, for intervention strategy, the sub-codes of “fidelity”, “dose”, “adaptations”, “reach” may be developed or “mechanisms” may be clustered around the sub-code “ideas”

### *Patient and Public Involvement*

A person living with dementia and a family carer of somebody living with dementia are members of the advisory board of the co-creation initiative. In this capacity they are overseeing the development of the study, provided feedback in the design phase of the intervention, reviewed the interview guides and are expected to review the results.

## ETHICS AND DISSEMINATION

Ethical and research governance approvals have been obtained from the West Midlands - South Birmingham Research Ethics Committee [REC reference 16/WM/0397].

The study has been designed as part of a 5-year co-creation initiative by the local working group in collaboration with the research team. Regular updates about the implementation of the intervention are regularly shared with the working group and with the wider group of stakeholders involved in the co-creation initiative.

Findings of the work will be disseminated across professionals e.g. at national conferences and events for providers and commissioners of care and support for people with dementia, policy makers in the area of dementia, representatives of services users. Articles stemming from this work will be submitted for publication to peer-reviewed journals and to national and international scientific conferences in the field of applied health research.



For peer review only - http://bmjopen.bmj.com/site/about/guidelines.xhtml

## REFERENCES

1 Dalmer NK. 'Add info and stir': an institutional ethnographic scoping review of family care-givers' information work. *Ageing Soc* 2018;:1–27. doi:10.1017/S0144686X18001125

2 Harris R, Wathen N, Wyatt S. Working to be healthy: empowering consumers or citizens? In: Harris R, Wathen N, Wyatt S, eds. *Configuring Health Consumers: Health Work and the Imperative of Personal Responsibility*. New York, NY: : Palgrave Macmillan 2010. 211–24.

3 Dalmer N. Informing care: Mapping the social organization of families' information work in an aging in place climate. *Electron Thesis Diss Repos* 2018.

4 Washington KT, Meadows SE, Elliott SG, *et al.* Information needs of informal caregivers of older adults with chronic health conditions. *Patient Educ Couns* 2011;**83**:37–44. doi:10.1016/j.pec.2010.04.017

5 Mastel-Smith B, Stanley-Hermanns M. 'It's like we're grasping at anything': Caregivers' education needs and preferred learning methods. *Qual Health Res* 2012;**22**:1007–15. doi:10.1177/1049732312443739

6 Dunbrack J. The information needs of informal caregiversinvolved in providing support to a critically ill loved one. 2005.

7 Vaingankar JA, Subramaniam M, Picco L, *et al.* Perceived unmet needs of informal caregivers of people with dementia in Singapore. *Int Psychogeriatrics* 2013;**25**:1605–19. doi:10.1017/S1041610213001051

8 Prorok J, Horgan S, Seitz D. Health care experiences of people with dementia and their caregivers: a meta-ethnographic analysis of qualitative studies. *CMAJ* 2013;**185**:E669-80.

9 Greenwood N, Habibi R, Smith R, *et al.* Barriers to access and minority ethnic carers' satisfaction with social care services in the community: A systematic review of qualitative and quantitative literature. *Heal Soc Care Community* 2015;**23**:64–78. doi:10.1111/hsc.12116

10 Afram B, Verbeek H, Bleijlevens MHC, *et al.* Needs of informal caregivers during transition from home towards institutional care in dementia: A systematic review of qualitative studies. Int. Psychogeriatrics. 2015;**27**:891–902. doi:10.1017/S1041610214002154

11 Sutcliffe CL, Roe B, Jasper R, *et al.* People with dementia and carers' experiences of dementia care and services: Outcomes of a focus group study. *Dementia* 2015;**14**:769–87. doi:10.1177/1471301213511957

12 Downs M, Clibbens R, Rae C, *et al.* What Do General Practitioners Tell People with Dementia and their Families about the Condition? *Dementia* 2002;**1**:47–58. doi:10.1177/147130120200100106

13 Van Hout HP, Vernooij-Dassen MJ, Jansen DA, *et al.* Do general practitioners disclose correct information to their patients suspected of dementia and their caregivers? A prospective observational study. *Aging Ment Heal* 2006;**10**:151–5. doi:10.1080/13607860500310468

14 Allen F, Cain R, Meyer C. Seeking relational information sources in the digital age: A



For peer review only - http://bmjopen.bmj.com/site/about/guidelines.xhtml

study into information source preferences amongst family and friends of those with dementia. Dementia. 2018. doi:10.1177/1471301218786568

15 Wackerbarth SB, Johnson MMS. Essential information and support needs of family caregivers. *Patient Educ Couns* 2002;**47**:95–100. doi:10.1016/S0738-3991(01)00194-X

16 Koehn S. Negotiating candidacy: ethnic minority seniors' access to care. *Ageing Soc* 2009;**29**:585–608. doi:10.1017/S0144686X08007952

17 Kelly F, Innes A. Facilitating independence: The benefits of a post-diagnostic support project for people with dementia. *Dementia* 2016;**15**:162–80. doi:10.1177/1471301214520780

18 Wilson TD. Models in information behaviour research. *J Doc* 1999;**55**:249–70. doi:10.1108/EUM0000000007145

19 Harland JA, Bath PA. Understanding the information behaviours of carers of people with dementia: A critical review of models from information science. Aging Ment. Heal. 2008;**12**:467–77. doi:10.1080/13607860802224300

20 Pinnock H, Barwick M, Carpenter CR, *et al.* Standards for Reporting Implementation Studies (StaRI) Statement. *BMJ* 2017;**356**:i6795. doi:10.1136/bmj.i6795

21 Pinnock H, Barwick M, Carpenter CR, *et al.* Standards for Reporting Implementation Studies (StaRI): explanation and elaboration document. *BMJ Open* 2017;**7**:e013318. doi:10.1136/bmjopen-2016-013318

22 Susman G, Evered R. An assessment of the scientific merits of action research. *Adm Sci Q* 1978;**23**:582–603.

23 Sustersic M, Gauchet A, Foote A, *et al.* How best to use and evaluate Patient Information Leaflets given during a consultation: a systematic review of literature reviews. *Heal Expect* 2017;**20**:531–42. doi:10.1111/hex.12487

24 NHS Digital. QOF 2018/19. https://qof.digital.nhs.uk/ (accessed 6 Mar 2020).

25 Proctor EK, Powell BJ, McMillen JC. Implementation strategies: recommendations for specifying and reporting. *Implement Sci* 2013;**8**:139. doi:10.1186/1748-5908-8-139

26 Mazza D, Bairstow P, Buchan H, *et al.* Refining a taxonomy for guideline implementation: results of an exercise in abstract classification. *Implement Sci* 2013;**8**:32. doi:10.1186/1748-5908-8-32

27 Powell BJ, McMillen JC, Proctor EK, *et al.* A compilation of strategies for implementing clinical innovations in health and mental health. Med. Care Res. Rev. 2012;**69**:123–57. doi:10.1177/1077558711430690

28 Proctor E, Silmere H, Raghavan R, *et al.* Outcomes for implementation research: conceptual distinctions, measurement challenges, and research agenda. *Adm Policy Ment Health* 2011;**38**:65–76. doi:10.1007/s10488-010-0319-7

29 Moore GF, Audrey S, Barker M, *et al.* Process evaluation of complex interventions: Medical Research Council guidance. *BMJ* 2015;**350**:h1258. doi:10.1136/bmj.h1258

30 Hoffmann TC, Glasziou PP, Boutron I, *et al.* Better reporting of interventions: Template for intervention description and replication (TIDieR) checklist and guide. *BMJ* 2014;**348**. doi:10.1136/bmj.g1687

31 Curran GM, Bauer M, Mittman B, *et al.* Effectiveness-implementation hybrid designs:



For peer review only - http://bmjopen.bmj.com/site/about/guidelines.xhtml

combining elements of clinical effectiveness and implementation research to enhance public health impact. *Med Care* 2012;**50**:217–26. doi:10.1097/MLR.0b013e3182408812

32 Pawson R, Tilley N. *Realistic Evaluation*. Thousand Oaks, CA: : SAGE publications 1997.

33 Astbury B, Leeuw FL. Unpacking Black Boxes: Mechanisms and Theory Building in Evaluation. *Am J Eval* 2010;**31**:363–81. doi:10.1177/1098214010371972

34 Greenhalgh T, Humphrey C, Hughes J, *et al.* How do you modernize a health service? A realist evaluation of whole-scale transformation in london. *Milbank Q* 2009;**87**:391–416. doi:10.1111/j.1468-0009.2009.00562.x

35 Dalkin SM, Greenhalgh J, Jones D, *et al.* What's in a mechanism? Development of a key concept in realist evaluation. *Implement Sci* 2015;**10**:49. doi:10.1186/s13012-015-0237-x

36 Dervin B, Nilan M. Information needs and uses. *Annu Rev Inf Sci Technol* 1986;**21**:3–33.

37 Savolainen R. The sense-making theory: Reviewing the interests of a user-centered approach to information seeking and use. *Inf Process Manag* 1993;**29**:13–28. doi:10.1016/0306-4573(93)90020-E

38 Johannessen A, Engedal K, Haugen PK, *et al.* "To be, or not to be": experiencing deterioration among people with young-onset dementia living alone. *Int J Qual Stud Health Well-being* 2018;**13**:1490620. doi:10.1080/17482631.2018.1490620

39 Samsi K, Manthorpe J. Everyday decision-making in dementia: findings from a longitudinal interview study of people with dementia and family carers. *Int Psychogeriatrics* 2013;**25**:949–61. doi:10.1017/S1041610213000306

40 Windle G, Joling KJ, Howson-Griffiths T, *et al.* The impact of a visual arts program on quality of life, communication, and well-being of people living with dementia: a mixed-methods longitudinal investigation. *Int Psychogeriatrics* 2018;**30**:409–23. doi:10.1017/S1041610217002162

41 Keller HH, Martin LS, Dupuis S, *et al.* Strategies to support engagement and continuity of activity during mealtimes for families living with dementia; a qualitative study. *BMC Geriatr* 2015;**15**:119. doi:10.1186/s12877-015-0120-2

42 Barnes M, Henwood F, Smith N. Information and care: A relational approach. *Dementia* 2016;**15**:510–25. doi:10.1177/1471301214527750

43 Moye J, Marson DC. Assessment of decision-making capacity in older adults: an emerging area of practice and research. *J Gerontol B Psychol Sci Soc Sci* 2007;**62**:P3–11.

44 Karel MJ, Gurrera RJ, Hicken B, *et al.* Reasoning in the capacity to make medical decisions: the consideration of values. *J Clin Ethics* 2010;**21**:58–71.

45 Karlawish J. Measuring Decision-Making Capacity in Cognitively Impaired Individuals. *Neurosignals* 2008;**16**:91–8. doi:10.1159/000109763

46 QSR International Pty Ltd. NVivo qualitative data analysis software. 2018.



For peer review only - http://bmjopen.bmj.com/site/about/guidelines.xhtml

## AUTHORS' CONTRIBUTIONS

CDP was involved in the design of the intervention and of the implementation strategy, conceived the evaluation framework, drafted the first version of the protocol and revised it following discussions with the other Authors. JO and MA oversaw the design of the intervention and of the implementation strategy, contributed to developing the evaluation framework and revised the manuscript. CB was involved in the design of the intervention and of the implementation strategy, supported local engagement and revised the manuscript. RG was involved in the design of the intervention and of the implementation strategy, supported local engagement and revised the manuscript. All authors have read and approved the final manuscript.

## FUNDING STATEMENT

CDP, JO, RG and MA were supported by The Health Foundation, grant number 1274233. CB received no specific grant from any funding agency in the public, commercial or not-for-profit sectors.

## COMPETING INTERESTS

None to declare

## FIGURES

Figure 1 - The phases of the co-creation initiative

Figure 2 – Timeline of the development of the intervention

Figure 3 – The evaluation framework of the intervention

Figure 4 – Data collection timeline

For peer review only

**Figure 1 - The phases of the co-creation initiative**

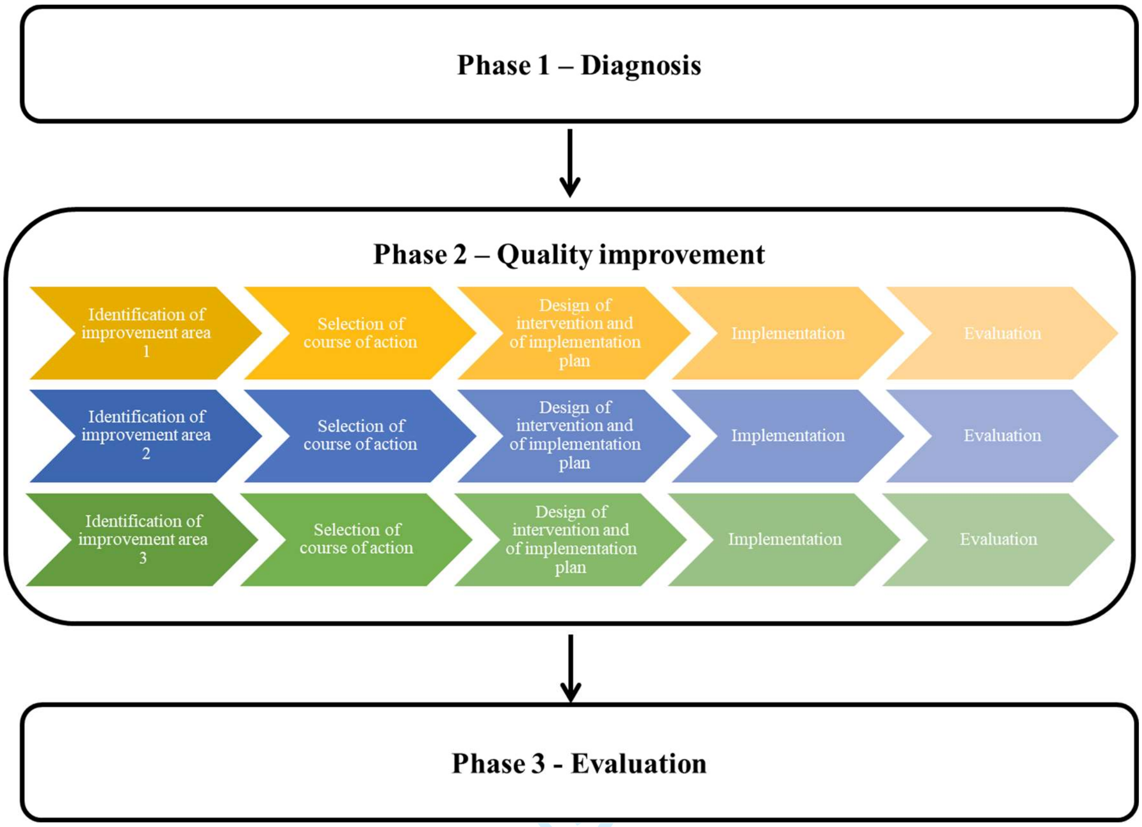


For peer review only - http://bmjopen.bmj.com/site/about/guidelines.xhtml

**Figure 2 – Timeline of the development of the intervention**

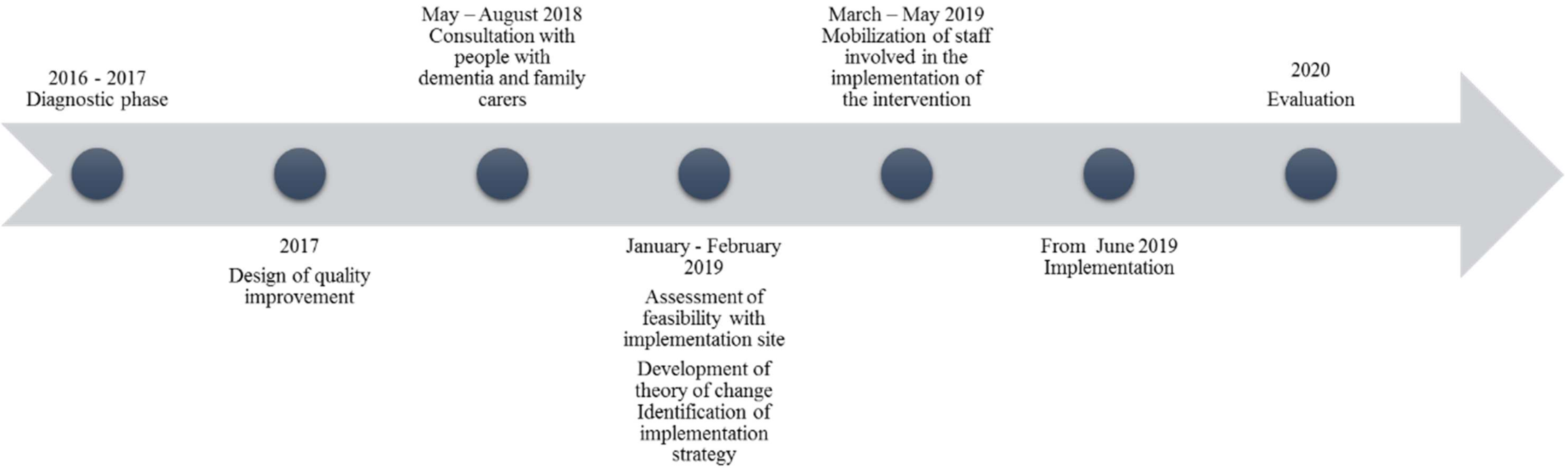

**Figure 3 – The evaluation framework of the intervention**

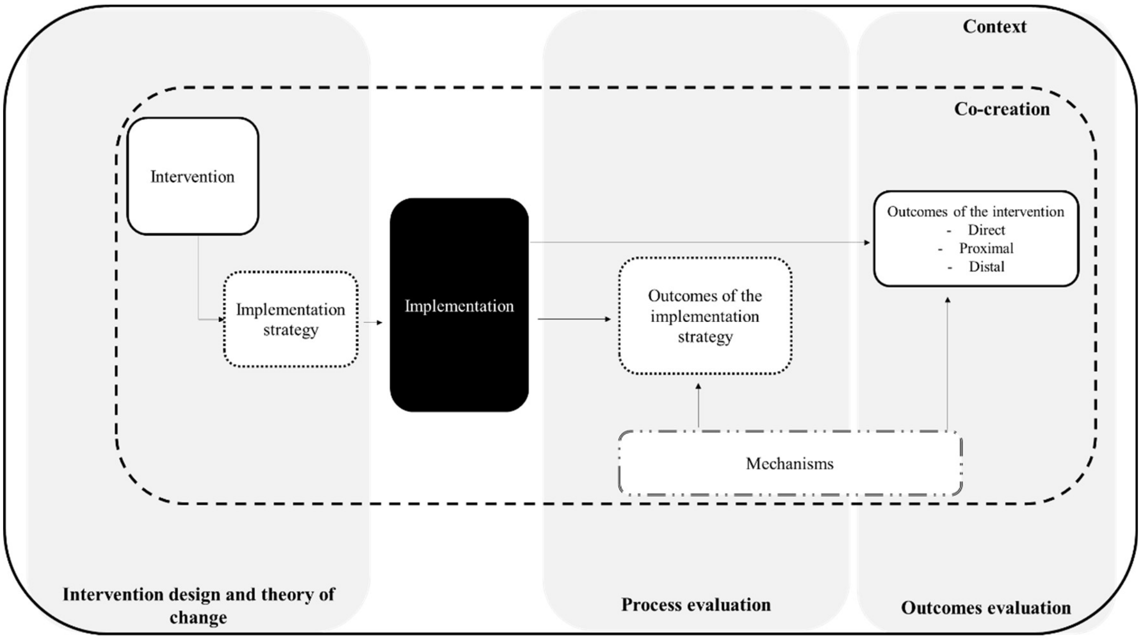


For peer review only

**Figure 4 – Data collection timeline**

| Month | | 1 | 2 | 3 | 4 | 5 | 6 | 7 | 8 | 9 | 10 | 11 | 12 |
|---|---|---|---|---|---|---|---|---|---|---|---|---|---|
| **Implementation** | | *Ongoing implementation* | | | | | | | | | | | |
| **People with dementia and family carers** | Recruitment | *Ongoing recruitment* | | | | | | | | | | | |
| | Data collection | | Interview 1 | | | | | | Interview 2 | | | | |
| **Implementers (CPNs, team and service managers)** | Data collection | | | | | | Interviews | | | | | | |
| **Local service providers** | Data collection | Activity log | | | | | | | | | | | |
| | | | | | | | | | | | Interviews | | |
| **Local working group** | Data collection | | | | | | | | | | | | Focus group |

**Supplementary material**

For peer review only

## Interview guide – People living with dementia (interview 1)

| | |
|---|---|
| Preamble | I'd like to ask you to tell me more about your experience of using this leaflet that I believe you recently received… |
| Receiving the leaflet | 1. Do you remember receiving this leaflet? When? Where? From whom? |
| | 2. When you were given the leaflet, did the supplier explained what information were available and how to use it? |
| Information seeking process before the leaflet was offered or alternative to the leaflet | 3. [If not recently diagnosed] – Before you were given the leaflet, how did you go about accessing information on dementia or local support available for yourself? How did you find this process? |
| | 4. Are you aware of similar sources of information? How have you found out about them? |
| | 5. What do you think of these other information materials available? |
| | 6. If you are aware of similar resources, have you used them? If not, why? |
| Expected or initial use | 7. What are your impressions of the new leaflet in terms of its usefulness? |
| | 8. Have you used it already? If yes, when did you use it? If not, do you think you will use it in the future? |
| | 9. If you used it, what particular information were you looking for? Could you find the information you were looking for? |
| | 10. Have you acted upon the information you located – e.g. have you already contacted/accessed a service? |
| | 11. Were there any particular aspects you think may be useful to you over the coming months? |
| Satisfaction | 12. Is there anything in particular you like or do not like about the leaflet? |
| | 13. Are there aspects of the leaflet you might want to change in order to make it better for your purposes? |
| Conclusions | 14. Is there anything else that you'd like to add? |
| Agreeing follow up interview | |
| Thank you | |

For peer review only

# Interview guide – People living with dementia (follow up)

| | |
|---|---|
| Preamble | I'd like to ask you to tell me more about your experience of using this leaflet that you received some months ago… |
| Use | 1. Since you have been given a copy by your CPN, have you used the leaflet? |
| | 2. If not, why? Do you plan to use it in the future? |
| | 3. If yes, when did you use it? What information were you looking for? Could you find what you were looking for? |
| | 4. Have you acted upon the information you found, e.g. have you contacted/accessed a service? |
| Barriers and facilitators | 5. What helped you to use the leaflet e.g. you received a printed copy or a trusted person gave it to you? |
| | 6. What prevented you from using the leaflet, e.g. format or content? |
| Information seeking process before the leaflet was offered or alternative to the leaflet | 7. Since we last spoke, have you found out about other information resources for people living with dementia? How have you found them? |
| | 8. If you are aware of similar resources, have you used them? If you haven't used them, why not? |
| | 9. What do you think of these other information resources? |
| Impact | 10. Has the leaflet changed the way you look for information about services available locally? If yes, in what way? If not, why? |
| | 11. Has the leaflet changed the care and support you receive? If yes, in what way? If not, why? |
| | 12. Since receiving the leaflet, are you in touch with organizations you previously did not know? |
| Satisfaction | 13. What do you like most/ least about the leaflet? |
| | 14. Is there anything you would change? |
| Future use | 15. Would you expect to receive a new copy of the leaflet if it is updated in the future? How often? From whom? |
| | 16. Would you keep using the leaflet in the future? |
| | 17. Is there anything else that you'd like to add? |
| Thank you | |



For peer review only - http://bmjopen.bmj.com/site/about/guidelines.xhtml

# Interview guide – Family carers (interview 1)

| | |
|---|---|
| Preamble | I'd like to ask you to tell me more about your experience of using this leaflet that I believe you recently received… |
| Receiving the leaflet | 1. Do you remember receiving this leaflet? When? Where? From whom? |
| | 2. When you were given the leaflet, did the supplier explained what information were available and how to use it? |
| Information seeking process before the leaflet was offered or alternative to the leaflet | 3. [If person with dementia not recently diagnosed] – Before you were given the leaflet, how did you go about accessing information on dementia or local support available for yourself or for <name of the person living with dementia>? How did you find this process? |
| | 4. Are you aware of similar sources of information for yourself or for <name of person living with dementia>? How have you found out about them? |
| | 5. What do you think of these other information materials available? |
| | 6. If you are aware of similar resources, have you used them? If not, why? |
| Expected or initial use | 7. What are your impressions of the new leaflet in terms of its usefulness? |
| | 8. Have you used it already? If yes, when did you use it? If not, do you think you will use it in the future? |
| | 9. If you used it, what particular information were you looking for? Could you find the information you were looking for? |
| | 10. Have you acted upon the information you located – e.g. have you already contacted/accessed a service for yourself or for <name of the person living with dementia>? |
| | 11. Were there any particular aspects you think may be useful to you over the coming months? |
| Satisfaction | 12. Is there anything in particular that you like or do not like about the leaflet? |
| | 13. Are there aspects of the leaflet you might want to change in order to make it better for your purposes? |
| Conclusions | 14. Is there anything else that you'd like to add? |
| Agreeing follow up interview | |
| Thank you | |

For peer review only

**Interview guide – Family carers (follow up)**

| | |
|---|---|
| Preamble | I'd like to ask you to tell me more about your experience of using the leaflet that you received some months ago… |
| Use | 1. Since you have been given a copy by your CPN, have you used the leaflet? |
| | 2. If not, why? Do you plan to use it in the future? |
| | 3. If yes, when did you use it? What information were you looking for? Could you find what you were looking for? |
| | 4. Have you acted upon the information you found, e.g. have you contacted/accessed a service for yourself or for <name of the person living with dementia>? |
| Barriers and facilitators | 5. What helped you to use the leaflet e.g. you received a printed copy or a trusted person gave it to you? |
| | 6. What prevented you from using the leaflet, e.g. format or content? |
| Information seeking process before the leaflet was offered or alternative to the leaflet | 7. Since we last spoke, have you found out about other information resources for carers of somebody living with dementia? How have you found them? |
| | 8. If you are aware of similar resources, have you used them? If you haven't used them, why not? |
| | 9. What do you think of these other information resources? |
| Impact | 10. Has the leaflet changed the way you look for information about local services for yourself or for <name of the person living with dementia>? If yes, in what way? If not, why? |
| | 11. Has the leaflet changed the care and support you or <name of the person living with dementia> receive? If yes, in what way? If not, why? |
| | 12. Since receiving the leaflet, are you in touch with organizations you previously did not know? |
| Satisfaction | 13. What do you like most/least about the leaflet? |
| | 14. Is there anything you would change? |
| Future use | 15. Would you expect to receive a new copy of the leaflet if it is updated in the future? How often? From whom? |
| | 16. Would you keep using the leaflet in the future? |
| | 17. Is there anything else that you'd like to add? |
| Thank you | |



For peer review only - http://bmjopen.bmj.com/site/about/guidelines.xhtml

## Interview guide – CPNs from Community Mental Health Services for Older People

| | |
|---|---|
| Preamble | I'd like to ask you about your experience of using the leaflet in clinical practice… |
| Implementation strategy | 1. How were you made aware that the leaflet would have been available to you to use in their clinical work? |
| | 2. Were you given any guidance about when and how to hand the leaflet out to patients? |
| | 3. Were you made aware of the type of patients who should have been given the leaflet? |
| | 4. How was the leaflet made available to you? E.g. was it stored somewhere on your IT system and you had to print it out? |
| | 5. When have you started using it? For how long will you use it in your clinical practice? |
| Actual use | 6. How are you using the leaflet with your patients, e.g. for signposting patients or families to local services? |
| | 7. Once you started using the leaflet in clinical practice, have you changed something in how you are using it? If yes, what have you changed? Why did you change it? |
| | 8. Do you think you reached the expected group(s) of users? |
| | 9. What are the factors that influenced the actual use of the leaflet in your clinical practice? |
| Impact | 10. Has the leaflet changed how your appointments with the patients and families take place? If yes, what has changed? Why has it changed? |
| | 11. Do you think that the leaflet has changed patients' and family carers' perceptions about local care and support available to them? |
| | 12. Do you think that the leaflet has changed how people with dementia and family carers access and use local services? |
| Future use | 13. Do you plan to keep using it? Do you think it should be updated regularly? |
| | 14. Do you think we should change anything about the leaflet (e.g. thinking about its content or its design) or about how it is used in clinical practice? |
| Other strategies | 15. Are you aware of the current strategies or priorities in the area of dementia (e.g. these could be national, local or identified by the organization you work for)? Do you think the leaflet fits with the current strategies or priorities? |
| Co-creation – Process | 16. Are you aware that the leaflet was designed and developed with inputs from both people living with dementia, their family carers, and a wide range of local organizations providing or commissioning care and support for them? If yes, did you contribute to the design and development of the leaflet? How? |



For peer review only - http://bmjopen.bmj.com/site/about/guidelines.xhtml

| | |
|---|---|
| Co-creation – Impact | 17. In your opinion did this approach influence the use of the leaflet in clinical practice? In what ways? |
| Conclusion | 18. Do you have any final comment on the leaflet? Or on your experience of using it with your patients and their families? Or on your experience of using it in clinical practice? |
| | 19. Is there anything else that you'd like to add? |
| Thank you | |

For peer review only

**Interview guide - Team and service managers from Community Mental Health Services for Older People**

| | |
|---|---|
| Preamble | I'd like to ask you about your experience of using the leaflet in your organization… |
| Implementation strategy | 1. Can you please describe how you organized the dissemination of the leaflet in practice among your staff? How did you inform the staff that the leaflet would have been made available to them to use in their clinical work? How you given your staff any guidance about when and how to hand the leaflet out to patients? |
| | 2. How you identified the type of patients who should have been given the leaflet? Was this made clear to your staff? |
| | 3. How was the leaflet embedded in the clinical practice of CPNs? |
| | 4. How is it made available to them e.g. was it stored somewhere on your IT system and CPNs can print it out? |
| | 5. When have you started using it? For how long have you planned to use it in practice? |
| | 6. Once you started using the leaflet in clinical practice, have you changed how it was originally planned to be used? If yes, what have you changed and why? |
| Actual use | 7. Are you aware of how the CPNs have been using the leaflet with their patients, e.g. for signposting users to local services available? |
| | 8. Do you think you reached the group(s) of users that you were expecting? |
| | 9. Are you aware of any specific factors that influenced the actual use of the leaflet by the CPNs in clinical practice? |
| Barriers and facilitators | 10. In your opinion, what facilitated and hindered the use of the leaflet in clinical practice? |
| Impact of the leaflet | 11. Has the leaflet changed how the appointments of the CPNs with their patients and families take place? Is this what you were expecting from the dissemination of the leaflet? |
| | 12. Do you think that the leaflet has changed patients' and family carers' perceptions about local care and support available? |
| | 13. Do you think that the leaflet has changed how people with dementia and family carers access and use local services? |
| Future use | 14. Do you plan to keep using the leaflet within your organization? Do you think it should be updated regularly? |
| | 15. Do you think we should change anything about the leaflet (e.g. thinking about its content or its design) or about how it is used in clinical practice? |
| Other strategies | 16. Are you aware of the current strategies or priorities in the area of dementia (e.g. these could be national, local or identified by the organization you work for)? Do you think the leaflet fits the current strategies or priorities? |



For peer review only - http://bmjopen.bmj.com/site/about/guidelines.xhtml

| | |
|---|---|
| Co-creation – Process | 17. Are you aware that the leaflet was developed with inputs from people living with dementia, their family carers, and several local organizations providing/commissioning dementia care? Did you contribute to the design and development of the leaflet? How? |
| Co-creation – Impact | 18. In your opinion did this approach influence the use of the leaflet in clinical practice? In what way? |
| Conclusion | 19. Do you have any final comment on the leaflet? Or on your experience of using it with your team/service? |
| | 20. Is there anything else that you'd like to add? |
| Thank you | |

For peer review only



For peer review only - http://bmjopen.bmj.com/site/about/guidelines.xhtml

**Interview guide – Managers from local service providers**

| | |
|---|---|
| Preamble | I'd like to review with you the data you have collected and I will need your help to make sense of them… |
| Review the activity log | 1. Can we have a look at the data covering the period in which the leaflet was used? How do these compare with the previous year? |
| Pattern of referrals | 2. Have you seen a change in the referrals that you received since the dissemination of the leaflet, e.g. in terms of<br>- who the referrers are (e.g. self referral or professionals)<br>- for what services the referrals are made<br>- for whom the referrals are made (i.e. person with dementia or family carers)<br>- when a referral is made (e.g. at a crisis point) |
| | 3. Do you think that the leaflet has changed the perceptions of people with dementia and family carers' about local care and support available? |
| | 4. Do you think that the leaflet has changed how people with dementia and family carers access and use local services? |
| Future use | 5. Do you think we should keep offering the leaflet to people with dementia and family carers in the future? |
| | 6. Do you think we should change anything in how/when/to whom the leaflet is offered? |
| Other strategies | 7. Are you aware of current strategies or priorities in the area of dementia (e.g. these could be national, local or identified by the organization you work for)? Do you think the leaflet meets the priorities of current strategies or priorities? |
| Co-creation – Process | 8. Are you aware that the leaflet was developed with input from both people living with dementia, family carers, and a wide range of local organizations providing or commissioning care and support? If yes, did you contribute to the development of the leaflet? How? |
| Co-creation – Impact | 9. In your opinion did this approach influence whether and how people with dementia and family carers used the leaflet? In what ways? |
| Conclusion | 10. Do you have any final comment on the leaflet? Or on your organisation's experience of using it with people with dementia and their families? Or on your organisation's experience of using it in clinical practice? |
| | 11. Is there anything else that you'd like to add? |
| Thank you | |



For peer review only - http://bmjopen.bmj.com/site/about/guidelines.xhtml

## Focus group guide – Leaflet working group

| | |
|---|---|
| Preamble | I'd like to share with you the feedback about the leaflet that we collected from different perspectives (users, implementers, local providers) and invite you to discuss them … |
| Feedback from people living with dementia and family carers | 1. These are some extracts from interviews carried out with a group of people living with dementia and family carers…. |
| Feedback from implementers (CPNs, team and service managers) | 2. These are some extracts from interviews carried out with a group of CPNs and with the team and service managers of the CMHSOP… |
| Feedback from third sector organizations | 3. These are some data about the trends in referrals into these organizations… |
| | 4. These are some extracts from the interviews carried out with the local providers… |
| Co-creation – Impact | 5. As you are aware, the leaflet was developed collaboratively, with input from different stakeholders. In your opinion did this approach influence whether and how people with dementia and family carers used the leaflet? In what ways? |
| | 6. Similarly, do you think that this approach has had an impact on the use of the leaflet by CPNs? |
| Updating the leaflet | 7. This is the feedback we collected about the leaflet, with some suggestions for improvement… |
| | 8. Do you have any other suggestions for improvement? |
| | 9. Do you have any suggestions for improving the process of dissemination of the leaflet? |
| | 10. Do you intend to update the leaflet and make it available to people living with dementia and family carers? How do you plan to go about this? |
| | 11. Is there anything else you would like to add? |
| Thank you | |



For peer review only - http://bmjopen.bmj.com/site/about/guidelines.xhtml

# Research checklist

For peer review only

**Overview of the implementation strategy reported in accordance with the StaRI checklist** [1]

| **IMPLEMENTATION STRATEGY (i.e. how the intervention was implemented)** | | |
|---|---|---|
| **Item** | **Description and reference to methodological frameworks and reporting standards** | |
| 1. Title | | Effectiveness-implementation hybrid type 2 study evaluating an intervention to support 'information work' in dementia care |
| 2. Abstract | | See Abstract of the protocol |
| 3. Introduction | To identify the problem | See Introduction of the protocol |
| 4. Introduction | Rationale for the implementation strategy and the intervention | See Intervention development of the protocol |
| 5. Aims and objectives | | 1. To assess the unfolding of the implementation strategy and its outcomes<br>2. To reflect on the extent to which, how and why the co-creation initiative has influenced the design of the implementation strategy and its unfolding and outcomes |
| 6. The design and key features of the evaluation | | The evaluation of the implementation strategy aims at understanding the feasibility of the intervention by investigating how the actual implementation process has unfolded in practice and identifying any discrepancies between the planned and the actual implementation process |
| 7. Context | Context in which the intervention is implemented | Community mental health services for older people (CMHSOP) within a mental health foundation trust in the North East of England (UK)<br>- North East of England is an under-researched area compared to other parts of England<br>- Local health economy with average recorded diagnosed dementia prevalence (for 2018-2019, local recorded prevalence rate 0.9% vs national recorded prevalence rate 0.8%) and average recorded annual review rate (for 2018-2019, local annual review rate 79.2% vs national recorded prevalence rate 77.9%)[2]<br>- Mental health foundation trust, established in 2006 providing a range of mental health, learning disability and eating disorder services for around two million people across a large and diverse geographical area<br>- CMHSOP providing community-based services for older people experiencing cognitive impairment and mental health problems. Organized around four localities, each locality team includes community psychiatric nurses (CPNs), occupational therapists, physiotherapists, psychologists, support workers to provide multi-disciplinary support<br>Intervention was developed in the context of a 5-year action research co-creation initiative funded by an English charity, involving researchers from three English universities and a |

For peer review only - http://bmjopen.bmj.com/site/about/guidelines.xhtml

<table>
<tr><th colspan="3">IMPLEMENTATION STRATEGY (i.e. how the intervention was implemented)</th></tr>
<tr><th>Item</th><th>Description and reference to methodological frameworks and reporting standards</th><th></th></tr>
<tr><td></td><td></td><td>research facilitator from the local commissioning support unit alongside a constellation of local stakeholders</td></tr>
<tr><td>8. Target sites</td><td></td><td>Four locality teams, staffing levels and caseload are reported in the table below
<table>
<tr><th>Team</th><th>Total staff</th><th>CPNs</th><th>Caseload</th></tr>
<tr><td>Team 1</td><td>48</td><td>9</td><td>1,368</td></tr>
<tr><td>Team 2</td><td>43</td><td>8</td><td>1,377</td></tr>
<tr><td>Team 3</td><td>27</td><td>5</td><td>787</td></tr>
<tr><td>Team 4</td><td>26</td><td>5</td><td>791</td></tr>
</table></td></tr>
<tr><td>9. Implementation strategy</td><td>Description of the implementation strategy in accordance with Proctor et al.'s framework for reporting implementation strategies[3]</td><td>Name and definition of the implementation strategy: bundle of professional and material interventions aiming at the development, adoption and use of the dementia leaflet among CPNs (based on framework by [4,5]<br><br>Specification of the implementation strategy<br>- Actors: Community Psychiatric Nurses (CPNs),team managers and service manager across four CMHSOP teams<br>- Actions: Described in accordance with relevant phase of the Plan-Do-Study-Act (PDSA) framework<br>  - Plan:<br>    Professional interventions<br>    o Established a local working group in charge of overseeing the development the leaflet and its implementation strategy<br>    o Iteratively developed a dementia leaflet, embedding feedback from people with dementia, family carers, professionals from local organisations providing care and support to people with dementia and family carers<br>    o Identified the implementation strategy via CPNs based in CMHSOP, identified local champion (service manager), identified implementation sites<br>    o Organised mobilisation activities with implementation sites, including information sessions at team meetings and email reminders<br>    Material intervention</td></tr>
</table>

For peer review only

For peer review only - http://bmjopen.bmj.com/site/about/guidelines.xhtml

| IMPLEMENTATION STRATEGY (i.e. how the intervention was implemented) | | |
|---|---|---|
| **Item** | **Description and reference to methodological frameworks and reporting standards** | |
| | | ○ Printed copies of the leaflet made available to implementers<br>- Do: CPNs offer a copy of the leaflet to people with dementia or their family carers attending an appointment (any type)<br>- *Action targets*: People living with dementia (any type of dementia, diagnosed at any time) and their family carers attending an appointment with their CPNs. CPNs were expected to use their clinical judgment in identifying patients or family members deemed to be ready to receive the leaflet<br>- *Temporality*: The leaflet can be offered at any point during the period in which the patient was under the care of the CMHSOP (at initial assessment, at post diagnosis, at reviews, at discharge)<br>- *Dose:* Depending on the individual readiness to receive the leaflet and individual and family circumstance, CPNs decide whether to offer or not the leaflet as part of their appointment with the patient. The leaflet could be offered more than once at different points in time |
| 10. Sub-groups | Any subgroups recruited for additional research tasks, and/or nested studies | Not applicable to this study |
| **Evaluation** | | |
| 11. Outcomes | Using Proctor et al.'s taxonomy of outcomes for implementation research [6] | - Acceptability of the intervention by CPNs: perception among CPNs that the intervention is agreeable, palatable, or satisfactory<br>- Adoption of the intervention by CPNs: CPNs' intention, initial decision, or action to try the intervention<br>- Appropriateness of the intervention implemented as judged by CPNs: perceived fit, relevance, or compatibility of the intervention for the setting of CMHSOP and perceived fit of the intervention to address the issue<br>- Cost of the intervention for the implementers<br>- Feasibility of the implementation of the intervention: the extent to which the intervention can be successfully used or carried out within the setting of the CMHSOP<br>- Penetration of the intervention: integration of the intervention within the CMHSOP<br>- Sustainability of the intervention: extent to which the intervention is maintained or institutionalised within the CMHSOP |
| 12. Process evaluation objectives and outcomes related to the mechanism(s) through which the strategy is expected to work | Objectives and outcomes related to the mechanism by which the strategy is expected to work, using MRC | - Fidelity of the intervention: adherence to the intervention protocol<br>- Adaptations of the intervention<br>- Dose: the quantity of intervention implemented |

For peer review only

| IMPLEMENTATION STRATEGY (i.e. how the intervention was implemented) | | |
|---|---|---|
| **Item** | **Description and reference to methodological frameworks and reporting standards** | |
| | framework for complex interventions [7] | - Reach: whether the intended audience comes into contact with the intervention, and how |
| 13. Data collection methods | | In-depth interviews with CPNs, team managers and service manager of CMHSOP |
| 14. Sample size | | - Up to 12 CPNs from four locality teams<br>- Four team managers<br>- One service manager |
| 15. Methods of analysis | | Thematic content analysis of interview data |

For peer review only

**Overview of the intervention reported in accordance with the StaRI checklist** [1]

| INTERVENTION (i.e. the actual intervention being implemented) | | |
|---|---|---|
| **Item** | **Description and reference to methodological frameworks and reporting standards** | |
| 1. Title | | Effectiveness-implementation hybrid type 2 study evaluating an intervention to support 'information work' in dementia care |
| 2. Abstract | | See Abstract of the protocol |
| 3. Introduction | To identify the problem | See Introduction of the protocol |
| 4. Introduction | Rationale for the implementation strategy and the intervention | See Intervention development of the protocol |
| 5. Aims and objectives | | 1. To assess the outcomes of the intervention as perceived by people living with dementia and their family carers, staff implementing the intervention and local organizations and to explain how and why the intervention contributed to achieve these outcomes<br>2. To reflect on the extent to which, how and why the co-creation initiative has influenced the design, implementation and impact of the intervention |
| 6. The design and key features of the evaluation | | The evaluation of the intervention aims at assessing the outcomes of the intervention with respect to people with dementia and family carers (primary outcomes), to the implementers of the intervention (proximal outcomes) and to local service providers (distal outcomes) |
| 7. Context | Context in which the intervention was implemented | As per implementation strategy described above |
| 8. Target population | Characteristics of the population targeted by the intervention and eligibility criteria | People living with dementia (any type of dementia, diagnosed at any time) and their family carers attending an appointment with their CPNs. CPNs were expected to use their clinical judgment in identifying patients or family members deemed to be ready to receive the leaflet |
| 9. Intervention | Description of the intervention, provided in accordance with the TIDieR checklist [8] | Name: Leaflet about local care and support available to people living with dementia and family carers<br><br>Why: in the diagnostic phase of the co-creation initiative, people living with dementia and family carers consistently reported a lack of timely, up-to-date information provided in a user-friendly format, which hinders their 'information work'. The intervention has been designed to provide a short, up-to-date, paper-based source of information that could be handed out and used by CPNs in their encounter with people with dementia and their family carers and support their 'information work' |

For peer review only

| INTERVENTION (i.e. the actual intervention being implemented) | | |
|---|---|---|
| **Item** | **Description and reference to methodological frameworks and reporting standards** | |
| | | What (materials): An A4 three-folded, two-sided leaflet printed in high contrast colour combination. Information are organized in four themes, for which relevant services, a short description and contact details are provided<br><br>What (procedures): CPNs offer the leaflet to patients and their family carers as part of their clinical encounters<br><br>Who: 27 qualified mental health nurses (CPNs) part of the organic cells working across four locality teams of the local CMHSOP who assess, diagnose, review medications and offer post-diagnostic support to people with dementia and signpost family carers to services available to them<br><br>How: the leaflet is offered in the context of the appointment attended by the person with dementia and their family carer. The CPN can offer the leaflet in response to a current specific need of the person with dementia or of the family carer (either explicitly reported or latent) or opportunistically, to make them aware of local services which could be of interest either at the present time or in the future<br><br>Where: the intervention takes place in the outpatient clinics of the CMHSOP services of the local mental health foundation trust<br><br>When and how much: CPNs can offer the leaflet at any point in the period when the patient is under the care of the CMHSOP (e.g. at initial assessment, at diagnosis, in the post diagnostic appointment, at review, upon transfer of care back to GP). It could be offered by the CPNs more than once at different points in time<br><br>Tailoring: CPNs are expected to use their clinical judgement in deciding whether, when, how and to whom to offer the leaflet based on<br>- patients or families' readiness to accept the leaflet (e.g. some patients do not temporarily - or will never - accept a diagnosis of dementia)<br>- length of the appointments (e.g. initial assessment can take up to 90 minutes and patients and families may be overloaded with information)<br>- type of appointments (e.g. titration appointments may cause disappointment in patients) |

For peer review only

| INTERVENTION (i.e. the actual intervention being implemented) | | |
|---|---|---|
| **Item** | **Description and reference to methodological frameworks and reporting standards** | |
| 10. Sub-groups | Any subgroups recruited for additional research tasks, and/or nested studies | Not applicable to this study |
| **Evaluation** | | |
| 11. Outcomes | Primary and other outcomes of the intervention | For people with dementia and family carers (primary outcomes)<br>- their satisfaction with the availability of information on local services (e.g. in terms of timing and quality of information)<br>- their awareness of local services<br>- in the longer term, their access and use of local services<br><br>For implementers of the intervention (proximal outcomes)<br>- CPN, team and service managers' awareness of local services and support available to people diagnosed with dementia and to their family carers<br>- CPN, team and service managers' confidence to hold conversations around using services available to those living with dementia and their family carers<br><br>For local service providers (distal outcomes)<br>- volume of referrals into their services<br>- appropriateness of the use of services among those living with dementia and family carers (e.g. with respect to the type and level of need) |
| 12. Process evaluation objectives and outcomes related to the mechanism(s) through which the strategy is expected to work | Objectives and outcomes related to the mechanism by which the strategy is expected to work, using MRC framework for complex interventions [7] | As per implementation strategy |
| 13. Data collection methods | | People with dementia and family carers<br>- Longitudinal semi-structured in-depth interviews: interview 1 carried out 4-6 weeks after the appointment with the CPN when they receive the leaflet; interview 2 (follow-up) scheduled about 6 months after interview 1<br><br>Implementers of the intervention<br>- Semi-structured in-depth interviews with CPNs, team managers and service manager of CMHSOP (scheduled about 6 months from the start of the implementation of the intervention) |

For peer review only

| INTERVENTION (i.e. the actual intervention being implemented) | | |
|---|---|---|
| **Item** | **Description and reference to methodological frameworks and reporting standards** | |
| | | Local service providers<br>- Activity log consisting of a table recording the monthly number of referrals received by the provider in total, by user group (people with dementia or family carer), by referral source (i.e. self-referral, CMHSOP, social services, other), by reason of the referral (e.g. health and safety assessment, information, support for carer, support for people with dementia), over a 24-month period (i.e. in the 12 months preceding the implementation of the intervention and during the 12-month implementation period)<br>- Semi-structured in-depth interviews with managers (scheduled about 10 months from the start of the implementation of the intervention)<br><br>Local working group<br>- focus group with about 12 participants (including commissioners, statutory service providers, staff from CMHSOP, managers from third sector organizations, a family carer) (scheduled about 12 months from the start of the implementation of the intervention) |
| 14. Sample size | | People living with dementia and family carers<br>- Up to 30 people living with dementia (any type of dementia, diagnosed at any time, willing and able to consent) and family carers (of somebody with any type of dementia, any degree of severity, diagnosed at any time)<br><br>Implementers of the intervention<br>- Up to 12 CPNs from four locality teams<br>- Four team managers<br>- Service manager<br><br>Local service providers<br>- at least three local organisations out of the seven listed on the leaflet as service providers which in preliminary conversations committed to supply data to inform the evaluation<br><br>Local working group<br>- about 12 participants attending the focus group (including commissioners, statutory service providers, staff from CMHSOP, managers from third sector organizations, a person with dementia and a family carer) |

For peer review only

| INTERVENTION (i.e. the actual intervention being implemented) | | |
|---|---|---|
| **Item** | **Description and reference to methodological frameworks and reporting standards** | |
| 15. Methods of analysis | | Thematic content analysis of interview and focus group data<br>Descriptive statistics for referral data provided by local service providers, triangulated with interview data |

For peer review only

## REFERECES

1 Pinnock H, Barwick M, Carpenter CR, *et al.* Standards for Reporting Implementation Studies (StaRI) Statement. *BMJ* 2017;**356**. doi:10.1136/bmj.i6795

2 NHS Digital. QOF 2018/19. https://qof.digital.nhs.uk/ (accessed 6 Mar 2020).

3 Proctor EK, Powell BJ, McMillen JC. Implementation strategies: recommendations for specifying and reporting. *Implement Sci* 2013;**8**:139. doi:10.1186/1748-5908-8-139

4 Mazza D, Bairstow P, Buchan H, *et al.* Refining a taxonomy for guideline implementation: results of an exercise in abstract classification. *Implement Sci* 2013;**8**:32. doi:10.1186/1748-5908-8-32

5 Powell BJ, McMillen JC, Proctor EK, *et al.* A compilation of strategies for implementing clinical innovations in health and mental health. Med. Care Res. Rev. 2012;**69**:123–57. doi:10.1177/1077558711430690

6 Proctor E, Silmere H, Raghavan R, *et al.* Outcomes for implementation research: conceptual distinctions, measurement challenges, and research agenda. *Adm Policy Ment Health* 2011;**38**:65–76. doi:10.1007/s10488-010-0319-7

7 Moore GF, Audrey S, Barker M, *et al.* Process evaluation of complex interventions: Medical Research Council guidance. *BMJ* 2015;**350**:h1258. doi:10.1136/bmj.h1258

8 Hoffmann TC, Glasziou PP, Boutron I, *et al.* Better reporting of interventions: Template for intervention description and replication (TIDieR) checklist and guide. *BMJ* 2014;**348**. doi:10.1136/bmj.g1687

For peer review only
